# Temporal dichotomy of neutrophil function in acute liver injury and repair

**DOI:** 10.1016/j.jhepr.2025.101417

**Published:** 2025-04-11

**Authors:** Jennifer A. Cartwright, Philippe M.D. Potey, Eilidh Livingstone, Lara Campana, Philip J. Starkey Lewis, Magdalena E.M. Oremek, Naomi N. Gachanja, Giulia Rinaldi, Rhona E. Aird, Tak Yung Man, Anuruddika J. Fernando, Joanna P. Simpson, Natalie Z.M. Homer, Nicole Barth, Melisande Addison, Candice Ashmore-Harris, Maria Elena Candela, Alastair M. Kilpatrick, Matthieu Vermeren, Calum T. Robb, David A. Dorward, Christopher D. Lucas, Stuart J. Forbes, Adriano G. Rossi

**Affiliations:** 1 Centre for Inflammation Research, Institute for Regeneration and Repair, University of Edinburgh, Edinburgh, UK; 2 Centre for Regenerative Medicine, Institute for Regeneration and Repair, University of Edinburgh, Edinburgh, UK; 3 The Royal (Dick) School of Veterinary Studies and the Roslin Institute, University of Edinburgh, Edinburgh, UK; 4 Mass Spectrometry Core, Edinburgh Clinical Research Facility, Centre for Cardiovascular Sciences, Queen’s Medical Research Institute, University of Edinburgh, Edinburgh, UK; 5 IRR Imaging Facility, Institute for Regeneration and Repair, University of Edinburgh, Edinburgh, UK

**Keywords:** Hepatic, Paracetamol, Acetaminophen, Formylated-peptide-receptor 1, Monocyte, Macrophage, Inflammation, Extracellular matrix remodeling

## Abstract

**Background & aims:**

Acetaminophen (APAP)-induced acute liver injury (APAP-ALI) is the leading cause of acute liver failure-induced death, with host innate immune responses driving outcomes. Neutrophils are activated and increased in APAP-ALI and reported to contribute to liver damage. However, neutrophil dysfunction in patients with acute liver failure is associated with non-survival, and recent reports highlight their importance in hepatic repair. Neutrophil-targeted therapies for APAP-ALI are hampered by this controversy and a lack of time-dependent investigation.

**Methods:**

Hepatic neutrophils were depleted at different times in a wild-type mouse model of APAP-ALI. *Fpr1*^*-/-*^ mice, with reduced neutrophil activation, were also used. The impact of neutrophil depletion was interrogated during hepatic injury and repair after APAP-ALI, using serum biochemistry, liver and blood flow cytometry, liver histopathology, immunohistochemistry, ELISA, and NanoString analysis.

**Results:**

Neutrophils contributed both to hepatic damage and repair after APAP-ALI. Early liver necrosis was reduced by neutrophil depletion (34% to 23%, *p* = 0.0018, n ≥10) and by reducing neutrophil functions (39% to 29%, *p* = 0.0279, n ≥11). By contrast, late neutrophil depletion resulted in markedly reduced liver repair (persistent necrosis 17% to 30%, *p* = 0.016, and higher serum alanine aminotransferase [1,221 to 3,725 IU/l, *p* = 0.0007, n ≥10]) and hepatocyte proliferation (decreased minichromosomal maintenance 2+ hepatocytes, 3% to 1%, *p* = 0.025, n = 10). Late neutrophil depletion reduced proliferation, growth factors, and angiogenesis transcripts (Mik6 fold change [FC] -6.322, *p* = 0.002; Socs2 FC -2.91, *p* = 0.01; vascular endothelial growth factor A FC -1.48, *p* = 0.01; n = 3). Similar transcript changes were identified when preventing formylated peptide receptor 1-mediated neutrophil activation, along with reduced extracellular matrix remodeling (Col12a1, FC -1.99, *p* = 0.0001; n ≥5). Finally, depleting neutrophils resulted in a hepatic proinflammatory monocyte/macrophage phenotype during repair stages, with increased proinflammatory-related transcripts and reduced reparative transcripts.

**Conclusion:**

Recruited neutrophils contribute not only to hepatic damage early in APAP-ALI, but also to hepatic repair through a variety of pathways, including extracellular matrix remodeling, angiogenesis, hepatocyte proliferation, and promotion of an anti-inflammatory monocyte/macrophage phenotype.

**Impact and implications:**

Novel therapies are required for APAP-ALI to improve patient outcomes. Neutrophil products and functions are potential targets for future therapies, but current literature controversy and a lack of time-dependent studies hinder progression. This study resolves the literature controversy, showing that neutrophils have time-dependent dichotomous roles in APAP-ALI. These insights highlight that early neutrophil-targeted interventions to reduce liver damage could be detrimental to subsequent patient recovery. Therefore, future research should aim to either elucidate isolated damaging functions or harness reparative functions of neutrophils for late-stage novel therapies for APAP-ALI.

## Highlights

• Neutrophils are recruited and activated after APAP-ALI and remain so during tissue repair.
• Neutrophils contribute to both early hepatic injury and late hepatic repair in APAP-ALI.
• AT7519 selectively and robustly reduces hepatic neutrophils after APAP-ALI initiation.
• Hepatic proliferation, ECM remodelling, growth factors and angiogensis are reduced following neutrophil depletion.

## Graphical abstract

**Figure:**
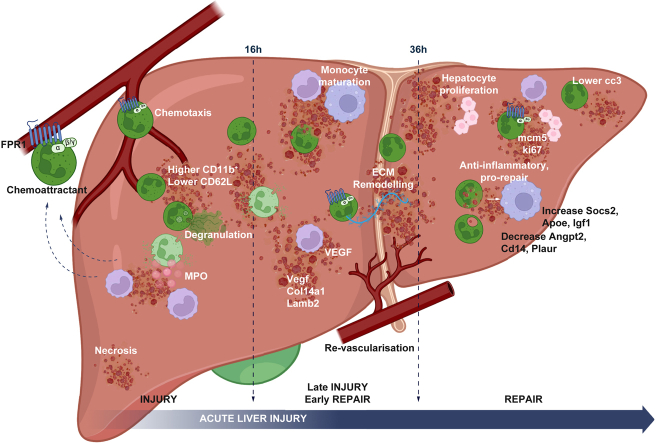

## Introduction

Acetaminophen (paracetamol; APAP)-induced acute liver injury (ALI), the leading cause of acute liver failure (ALF) in the Western world,[1], [2], [3] has no effective treatments for late presentations, and patients progressing to ALF have a worse prognosis compared with those with other causes of ALF.^4^ Patients with APAP-ALI have pronounced increases in circulating activated neutrophils,^5^^,^^6^ and hepatic necrosis is characterized by marked inflammation and early infiltration of neutrophils.^7^ Patients with APAP-ALI progressing to ALF have reduced neutrophil functions^8^^,^^9^ and such patients are at risk of fatal bacterial infections,^3^ indicating a potential crucial role of neutrophils in recovery from APAP-ALI.

Neutrophils, the most abundant human circulating leukocyte, provide crucial host defenses against invading pathogens. They are enigmatic cells with myriad immunological processes, functional plasticity, and heterogeneity.^10^^,^^11^ Numerous important neutrophil functions continue to emerge, such as during tumor metastasis, and in autoimmunity, chronic diseases,^12^ and, increasingly, tissue restoration.^13^ Although their uncontrolled activation can be detrimental,^14^ neutrophils are also known to orchestrate inflammation resolution and subsequent tissue repair.^15^^,^^16^

In APAP-ALI, neutrophils are recruited in response to numerous signals, including tissue damage-associated molecular patterns (DAMPs), particularly mitochondrial formylated peptides,^7^ which bind to formylated peptide receptor 1 (FPR1), resulting in receptor upregulation, and neutrophil activation^17^^,^^18^ and migration.^19^^,^^20^

APAP-ALI is difficult to investigate in patients because of many factors; therefore, mouse model studies are frequently used as an alternative.^21^ Cytopenias are a known negative risk factor for APAP-ALI^22^ and significant neutrophil function abnormalities have been related to outcomes in patients with ALF.^9^^,^^23^ The role of neutrophils in mouse models of APAP-ALI remains controversial, with several reports indicating that they potentiate hepatic tissue damage,^19^^,^^21^^,^[24], [25], [26] while others contest this, reporting their importance in hepatic repair.[27], [28], [29], [30]

Neutrophils can be modulated to resolve inflammation, and neutrophil products, such as membrane-derived nanovesicles and extracellular vesicles, have been considered as treatments for inflammatory conditions.^31^^,^^32^ However, the unresolved functions of neutrophils in APAP-ALI and their role in ALF and prevention of sepsis, impede discovery of potential neutrophil-targeted therapeutic strategies.

All previous studies of neutrophil function have assessed this in either injury or repair and none have evaluated their functions over time. To elucidate the neutrophil role over the course of APAP-ALI, we pharmacologically depleted neutrophils at different times in a wild-type (WT) mouse model, as well as using *Fpr1*^-/-^ mice, with reduced neutrophil activation. We assessed blood and tissue neutrophil activity alongside hepatic damage and tissue regeneration markers during APAP-ALI. Through these techniques, we show that circulating and hepatic neutrophils are activated rapidly after APAP and remain so during repair. We reveal a time-dependent dual role of neutrophils in APAP-ALI, with early depletion resulting in decreased hepatic necrosis during injury, and late depletion resulting in reduced repair. These findings are reinforced in *Fpr1*^*-/-*^ mice, demonstrating that neutrophils contribute to both hepatic tissue injury and repair through FPR1-mediated functions. Findings in both models highlight alterations in hepatic angiogenesis and extracellular matrix (ECM) remodeling during repair following neutrophil modulation, alongside an altered monocyte/macrophage phenotype. Our findings address the longstanding and continued controversy surrounding the role of neutrophils in APAP-ALI, and highlight the importance of considering neutrophil plasticity and time-dependent tissue context for therapeutic targeting.

## Materials and methods

All methods are additionally detailed in^33^ and the supplementary CTAT Table.

### Mice

C57BL/6 male mice were purchased from Charles River (Edinburgh, UK). *Fpr1*^-/-^ mice^34^ and human myeloid cell leukemia factor 1 (*hMcl1*) transgenic mice^35^ were propagated at the University of Edinburgh, both with WT colony controls. Mice housed in groups in ventilated cage systems were acclimatized for 1 week before experiments and synchronized to a 10–14 h dark/light cycle with access to food and water *ad libitum*. All experiments had local ethical approval, were conducted under UK Home Office legislation, and conformed to Animal Research: Reporting of In Vivo Experiments (ARRIVE) guidelines. Genotyping was carried out using PCR by TransnetYX.

### APAP model

Male mice (8–14-weeks old) were fasted for 12 h before receiving a 350 mg/kg i.p. injection of either APAP in sterile saline or sterile saline (control). Standard chow and mash were returned to mice 20 min after injection and all mice were maintained in warming cabinets (28 °C). Vehicle (sterile saline) or 30 mg/kg AT7519 (Astex Pharmaceuticals, Cambrdge, UK), a selective CDKI^36^ in sterile saline, were given i.p. at 4 h or 16 h post APAP. At predetermined times post APAP, mice were humanely culled according to local ethical guidelines and whole blood was collected. During APAP experiments, mice were monitored for six behavioral phenotypes of clinical severity (scoring 0–3); hunching, piloerection, neurological symptoms, responsiveness to touch, skin pallor, and breathing effort.

### Nonparenchymal cell labeling and flow cytometry analysis

Isolation of the hepatic nonparenchymal cell (NPC) fraction was achieved as detailed elsewhere^37^ with minor alterations. Briefly, mouse livers were perfused *in situ* with 10 ml 0.9% NaCl through the inferior vena cava. Livers were harvested and weighed, and 0.4 g of tissue was homogenized and digested in RPMI 1640 containing collagenase V (0.8 mg/ml; Sigma-Aldrich, Glasgow, UK), collagenase D (0.625 mg/ml; Roche, London, UK), dispase (1 mg/ml; Life Technologies, Cambridge, UK), collagenase D (1.6 mg/ml; Roche), and DNase I (100 mg/ml; Roche) for 25 min at 37 °C in a shaking incubator. Cold RPMI 1640 with 10% fetal calf serum (FCS) was added to 70 μm strained digests to deactivate enzymes. Immune cell-containing fractions were harvested by two wash centrifugations (300 g, 4 °C, 5 min), followed by red blood cell (RBC) lysis using 3 ml RBC lysis buffer (Sigma-Aldrich) for 3 min at room temperature (RT). Cells were counted, and stained with a Fixable Viability Dye eFlour™ 780 (Invitrogen 65-0865) for 30 min. Non-specific binding was blocked with 10% mouse serum (4 °C, 5 min), followed by incubation with primary antibodies (Table S1–S4), (40 min, 4 °C). Samples were then processed on a BD LSR Fortessa 4 laser and analyzed using FCSExpress software (DeNovo, Pasadena, USA). Absolute cell numbers were quantified per gram of liver through a proportion of counted liver digest NPCs, factoring for liver weight.

### Plasma chemistry evaluation

Serum analysis was performed by the Specialist Assay Service at the MRC Centre for Reproductive Health (University of Edinburgh, Edinburgh, UK), using a commercial kit (Alpha Laboratories, Hampshire, UK) for alanine aminotransferase (ALT) and albumin, on the Cobas Fara centrifugal analyzer (Roche Diagnostics Ltd, UK). Aspartate aminotransferase (AST), alkaline phosphatase (ALP), and glutamate dehydrogenase (GLDH) were quantified using commercial kits (Alpha Laboratories) on the Mira analyzer (Roche Diagnostics, UK).

### Multiplex Meso Scale Discovery ELISA assessments

Serum and liver cytokine concentrations were quantified using a Meso Scale Discovery (MSD) multiplex ELISA system. Liver was lysed with MSD lysis buffer (150 mM NaCl, 20 mM Tris, 1 mM EDTA, 1 mM ethylene glycol-bis(β-aminoethyl ether)-N,N,N′,N′-tetraacetic acid) (EGTA), 1% TritonX-100, 2x protease inhibitor cocktail, [Sigma Aldrich]) and homogenized in a Precelly’s tissue homogenizing tube (P000918-LYSK0-A) (two cycles of 2,000 g, 30 s). The liver lysis supernatant and serum protein were quantified with a Pierce™ BCA protein microplate assay and measured using a MESO QuickPlex SQ 120 plate reader (562 nm absorbance). Samples were assessed on MSD® mouse proinflammatory panel 1 V-PLEX™ plates containing ten multiplexed cytokines (IFNγ, IL10, IL12p70, IL1β, IL2, IL4, IL5, IL6, CXCL1, and TNFα) using a QuickPlex SQ 120 analyzer (MSD).

### Mouse serum liquid chromatography-mass spectrometry

AT7519 and a deuterated standard ^2H^_8_-AT16043M (d8-AT7519) were gifted by Astex Pharmaceuticals. APAP was purchased from Apollo (Denton, UK) and ^2H^_4_-APAP (d4-APAP) from Cerilliant® (Merck, Watford, UK). Following a previously described method,^38^ samples were injected into a Waters Acquity UPLC BEH C18 column (2.1x100 mm, 1.7 μm; Waters, Wilmslow, UK) maintained at 45 °C. Mass analysis was performed on a QTrap 5500 triple quadrupole mass spectrometer (AB Sciex, Warrington, UK) in positive ion mode. Liquid chromatography (LC)-mass spectrometry (MS)/MS data were collected using Analyst® 1.7.1 software.

### RNA isolation and NanoString

An AllPrep® DNA/RNA FFPE kit (Qiagen, Manchester, UK) was used for purification of RNA from formalin-fixed paraffin-embedded (FFPE) tissues for use with the nCounter® Mouse Myeloid Innate Immunity V2 Panel plate (NanoString, Washington, USA). Briefly, RNA was extracted from 10 μ-thick sections using RNeasy MinElute spin columns (Qiagen) according to the manufacturer’s protocol. RNA was quantified using the Nanodrop Spectrophotometer (Thermo Fisher Scientific) and RNA quality was assessed using a LabChip® GX Touch/GXII Touch nucleic acid analyzer (PerkinElmer, Springfield, USA) (QMRI Biomolecular Core, University of Edinburgh). Samples were processed by the Host and Tumour Profiling Unit (University of Edinburgh, UK) following the manufacturer’s guidelines, using the nCounter® Prep station and nCounter® Analysis system (NanoString).

### NanoString data analysis

nSolver 4.0 Analysis Software (module 2.0.134, NanoString) was utilized for *Fpr1*^-/-^ *vs.* WT analysis, using the advanced analysis 2.0 plug-in. Background thresholding was increased to 50 probe counts and comparisons were set between genotypes. After removal of low raw counts and QC flags, a strict requirement was set for a log_2_ fold change with the threshold of >1.25 or <-1.25 and *p* <0.05.

ROSALIND® (https://rosalind.bio/; San Diego, CA, USA), was used to analyze AT7519 *vs.* vehicle data. nCounter® reporter code count data were imported and files were annotated. Datasets were analyzed with ROSALIND HyperScale architecture. GeNorm selected normalization housekeeping probes were used from the NormqPCR R library.^39^ Benjamini–Hochberg *p* value adjustments were performed and Partitioning Around Medoids was used for the final clustering of genes. This used the fpc R library^40^ considering the direction and type of all signals on a pathway, and the position, role and type of every gene. Analysis comparing groups was completed with a threshold fold change of ≥ 1.25 or ≤ -1.25 and a significance set at *p* <0.05.

### Immunohistochemistry

Liver tissue was harvested and fixed in 4% paraformaldehyde (PFA) followed by 70% ethanol before paraffin embedding. Heat-induced epitope retrieval of deparaffinized tissue sections was in 0.01 M sodium citrate buffer (pH 6), or TrisEDTA (pH 8) for 15 min, or Proteinase K for 10 min at 37 °C depending on the antibody. For major basic protein (MBP), Digest-All™ 3 pepsin was used for 10 min and the wash buffer was 0.05 M Tris-HCl, 0.15 M NaCl, 0.05% Tween 20, pH 7.6. For minichromosomal maintenance (MCM)-2, sections were permeabilized in PBS 0.1% Tween 20 (PBST) for 5 min, followed by wash steps including PBST. Sections visualized with 3,3′-diaminobenzidine (DAB) were sequentially blocked at RT with Bloxall (Vector, Newark, CA, USA), Avidin, and Biotin (Invitrogen, Cambridge, UK) for 15 min each, followed by Protein Block (Spring Bio). Sections for immunofluorescence were blocked for 30 min with Protein Block. DAB and immunofluorescent sections were incubated overnight at 4 °C with primary antibodies (Table S5 and supplementary CTAT Table). For DAB stains, sections were incubated (1 h at RT) with a biotinylated secondary antibody (Table S6), followed by 30 min avidin-based peroxidase reagent R.T.U. VECTASTAIN Elite ABC reagent (Vector) and DAB (DAKO,Glostrup, Denmark) and Harris’ hematoxylin counterstain. Immunofluorescence labeling was completed with secondary conjugated antibodies (Invitrogen 1:200) and DAPI (1:1,000).

### Microscopy and imaging analysis

Fluorescent and bright field images were acquired on a Nikon Eclipse e600 microscope with a Retiga 200R camera (Q-imaging, Image Pro premier software), DMi8 (Leica Microsystems, Milton Keynes, UK), Zeiss Axioskop microscope, or EVOS M7000 Imaging System (Thermo Fisher Scientific). Bright field images were also acquired on a Vectra® Polaris™ multi spectral slide scanner (PerkinElmer), with fluorescent images collected on an Operetta CLS High Content Analysis System (PerkinElmer). DAB-positive cell quantification was completed with inForm 2.4 (PerkinElmer) and inForm spectral unmixing tissue segmentation was utilized to quantify necrosis. Numbers and percentage of immunofluorescent positive cells were analyzed using Columbus™ software (PerkinElmer). Hepatic cytochrome P450 2E1 (Cyp2e1) quantification was analyzed using Fiji ImageJ (ImageJ Software, National Institute of Health, Bethesda, MD, USA: http://rsb.info.nih.gov/ij/)). FPR1-labeled sections were imaged with a ZIESS observer 7 and adapted for publication with ImageJ.

### Isolation of mouse bone marrow neutrophils

Mouse long bone marrow was flushed into a 50 ml conical tube with Hank’s buffered salt solution (HBSS)-prep (Ca–Mg-free HBSS, 20 mM Na-HEPES, 0.5% FCS, 1% penicillin and streptomycin) using a 25-g needle. Bone marrow was disaggregated and cells were pelleted by centrifugation (400 **g**, 5 min). The pellet was resuspended in 5 ml 0.2% NaCl for 45 s to lyse RBCs and osmolarity was restored with 5 ml of 4 °C 1.6% NaCl. Cell strained (40 μm) suspensions were centrifuge (400 **g**, 5 min) and re-suspended in 5 ml HBSS-prep, then layered on 5 ml of 62% Percoll in a 15-ml falcon tube. This was centrifuged at 1,000 **g** for 30 min (acceleration 5, 0 brake). Pelleted cells and immature granulocytes were resuspended, counted, and then centrifuged and resuspended in HBSS-prep to 10x10^6^/ml.

### *In vitro* neutrophil activation assay

After separation, granulocytes were diluted to 20x10^6^/ml in HBSS^+/+^ and 180 μl was added to a 2-ml Eppendorf/reaction tube with 20 μl of activation reagent (formylated peptide, platelet activating factor [PAF], or control PBS). This was agitated for 30 min at 37 °C. For shape change analysis, cells were fixed with 4% PFA (15 min). For assessment of activation markers, cells were transferred to fluorescence-activated cell sorting (FACS) tubes with conjugated antibodies (Ly6G, CD11b, and CD62L, all at 1:100) and incubated at RT for 30 min before washing and fixing. Cells were then analyzed with a BD LSR Fortessa 4 laser, or BD Accuri C6.

### Study design

Statistical power analysis, to identify mouse sample size estimation for AT7519 treatment experiments, was performed based on *in vivo* data from a published study^41^ where n = 6, which compared untreated and cyclin-dependent kinase inhibitor (CDKI)-treated mice with acute pulmonary inflammation using a significance of *p* <0.05. Depending on the translatable measured variable, the effect size (ES) in this study was 1.16–3.2, considered to be large to very large using Cohen's criteria.^42^ With an alpha = 0.05 and power = 0.80, the projected sample size needed, with a median ES of 1.5, was n = 9 for a between-group comparison. A similar sample size estimation of n = 8 for the comparison of WT and FPR1^-/-^ mice was identified using published data with a model of acute pulmonary inflammation at *p* <0.05.^43^ Unpublished necrosis comparison data from APAP-treated mice with and without interventions (Forbes *et al.*, unpublished data, 2025), gave an ES of 1.96. Statistical power analysis for a one-way ANOVA gave a required n = 9. All tests were performed using commercially available software, G∗Power.^44^ Therefore, the proposed sample size was set as a minimum of nine for APAP-ALI studies for all genotypes and interventions. This was determined to be adequate for the main objective of this study, allowing for expected attrition and controlling for possible mediating and moderating factors and subgroup analysis.

### Data analysis and statistics

Unless otherwise stated, statistical analysis was performed in Prism 9.4 (GraphPad software). All data are presented as individual scatter plots and show each experimental data point (*e.g.* individual mice) unless otherwise stated. Any data with a small n (<6) were treated as nonparametric. Gaussian distribution was otherwise assessed by a Shapiro–Wilk normality test. When normally distributed, the mean is expressed on all graphs, whereas medians are shown for nonparametric data. To test two groups, an unpaired 2-way *t* test with or without Welch’s correction for different group variance, or a Mann-Whitney (MW) *U* test or Kolmogorov–Smirnov (KS) test for cumulative distribution was performed on parametric and nonparametric datasets, respectively. To test two or more parametric groups, a one-way ANOVA, two-way ANOVA (with Dunnett's multiple comparison test), or mixed-effects model (with Sidak's multiple comparison's test) was performed. To test two or more nonparametric groups, a Kruskal–Wallis test (with Dunn's multiple comparison test) was performed. *p* <0.05 was considered statistically significant.

## Results

### Depleting neutrophils in APAP-ALI, reduces hepatic injury and repair

**Fig. 1:**
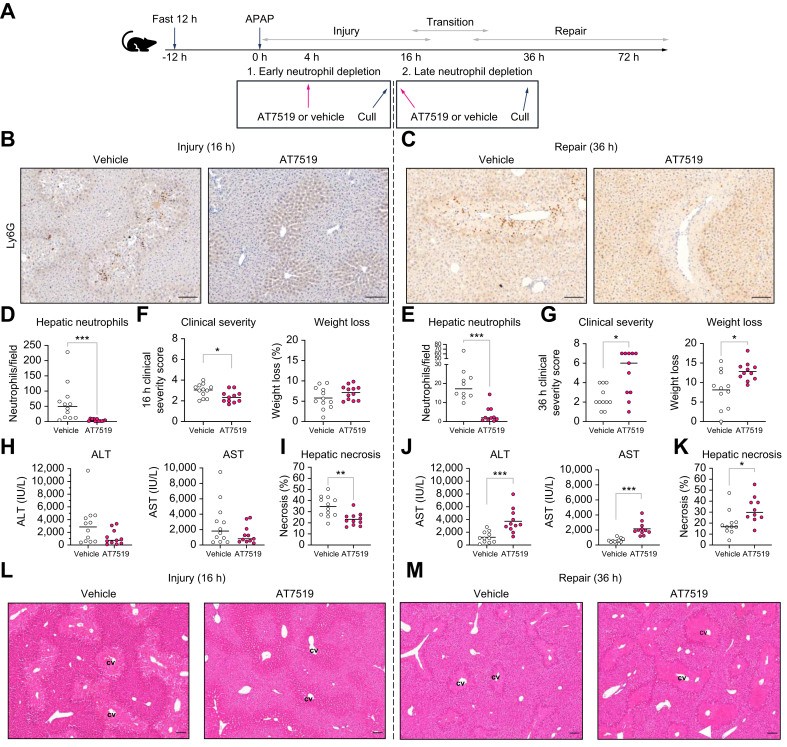
AT7519-mediated depletion of neutrophils reduces hepatic injury and repair. (A) Model schematic. (B,C) Representative Ly6G-labeled hepatic sections, displaying AT7519-reduced neutrophils, analyzed with KS (D) *p* = 0.0001 and (E) *p* = 0.0006. (F) Early neutrophil depletion improved clinical severity (*t* test, *p* = 0.0254). (G) Clinical severity (MW, *p* = 0.012) and weight loss (*t* test, WC, *p* = 0.014) worsened during repair. (H) ALT and AST during injury. (J) Elevated ALT (*t* test *p* = 0.0007) and AST (*t* test, WC, *p* = 0.0001) during repair. (I) Early neutrophil depletion reduced necrosis (*t* test, *p* = 0.0018). (K) Necrosis increased during repair (MW, *p* = 0.016). (L,M) Representative H&E hepatic sections showing necrosis around CVs. N ≥10 in all cases; scale bars: 100 μm. ALT, alanine aminotransferase; AST, aspartate aminotransferase; CV, central veins; KS, Kolmogorov–Smirnov; WC, Welch’s correction; MW, Mann-Whitney. In all instances: ∗*p* <0.05, ∗∗*p* <0.005, ∗∗∗*p* <0.0005, ∗∗∗∗*p* <0.0001.

To interrogate the controversial role of neutrophils during APAP-ALI over time, neutrophils were depleted during early (4 h, before peak necrosis) or late (16 h, after peak necrosis) injury in a mouse model of APAP-ALI, using a single i.p. injection of AT7519, a CDKI shown to deplete neutrophils.^45^^,^^46^ Pharmacological depletion was used given the known limitations of anti-Ly6G or anti-Gr-1,^47^ which was in agreement with our findings that neutrophils were ineffectively depleted by intravenous Ly6G after established APAP-ALI. Assessments were completed during both injury and repair phases after establishing these timings in the WT mouse model (Fig. 1A; Fig. S1A–D). AT7519, given either during early injury or at peak hepatic neutrophil recruitment as assessed by immunohistochemistry (Fig. S1E and F) and flow cytometry (Figs S1G,H and S2), dramatically reduced hepatic neutrophil numbers (Fig. 1B–E). Early hepatic neutrophil depletion was associated with a significant early reduction in hepatic damage, with reduced mouse clinical severity score and hepatic necrosis. When depletion was performed late, at peak neutrophil infiltration (16 h) with assessment during hepatic repair, mice showed more weight loss and higher clinical severity. Serum biomarkers of hepatic injury (ALT, AST, and GLDH) were also higher along with persistent hepatic necrosis (Fig. 1E–M). This depletion dichotomy illustrated the time-dependent role of neutrophils in APAP-ALI.

### AT7519-mediated neutrophil depletion is neutrophil specific

The inflammation resolution mediated by early AT7519 neutrophil depletion shown here is commensurate with published studies of AT7519 improving return of tissue homeostasis and animal recovery.^45^^,^^46^^,^^48^ However, AT7519-mediated neutrophil depletion has not been previously associated with reduced tissue repair. Therefore, further assessments investigated the neutrophil specificity of this reduced repair phenotype. AT7519 30 mg/kg had no effect on hepatic injury biomarkers in APAP control-treated (healthy) mice and caused no hepatic necrosis or increased hepatic cleaved caspase 3 (CC3) expression. There was also no reduction in hepatic proliferation (Fig. S3A–G). A lower AT7519 dose of 10 mg/kg was insufficient to deplete hepatic neutrophils in APAP-ALI (Fig. S3H) and there was also no alteration in hepatic repair without neutrophil depletion (Fig. S3I–M). Hepatic Cyp2e1 expression, the main enzyme that metabolizes APAP into its toxic metabolite *N*-acetyl-p-benzoquinoneimine, was unaltered after AT7519 in healthy or APAP-ALI mice (Fig. S3N). Mass spectrometry analysis of serum APAP and AT7519 identified no correlation between AT7519 and APAP concentrations, although AT7519 was higher in animals with APAP toxicity (Fig. S4A–E), indicating reduced metabolism of this drug following APAP-ALI. There was no correlation of serum AT7519 concentration with measures of hepatic damage, hepatic necrosis, and hepatic CC3, or with hepatic proliferation (Fig. S4F–I).

### Hepatic and circulating neutrophils are activated following APAP-ALI

**Fig. 2:**
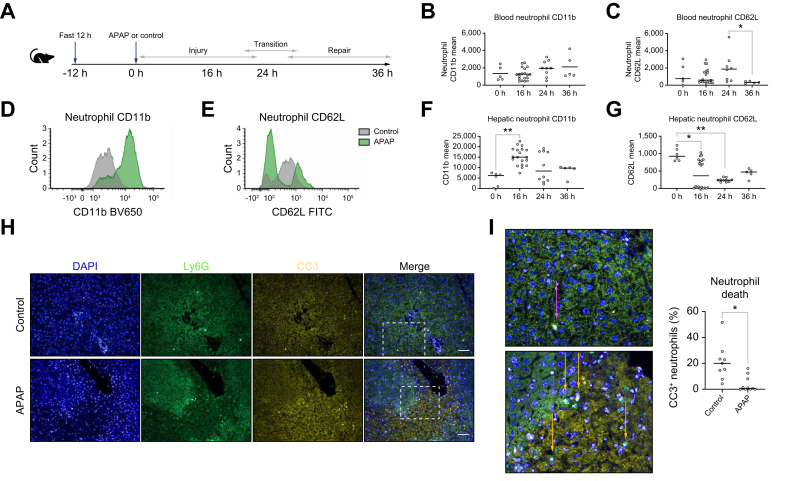
Circulating and hepatic neutrophils are activated following APAP with prolonged survival. (A) Model schematic. (B) Blood neutrophil CD11b expression (KW, *p* = 0.1899). (C) Decreased blood neutrophil CD62L expression, following APAP (KW, *p* = 0.0456, 24 h *vs.* 36 h, *p* = 0.0344). (D,E) Representative hepatic neutrophil flow cytometry. (F) Increased neutrophil CD11b following APAP (KW, *p* = 0.00008; 0 *vs.* 16, *p* = 0.0015) and (G) decreased CD62L expression (KW, *p* = 0.00088; 0 h *vs.* 16 h, *p* = 0.0126; 0h *vs.* 24 h, *p* = 0.0086. (H) Representative Ly6G and CC3 hepatic sections at 36 h post APAP administration and in controls. (I) Magnifications showing CC3+ (pink arrows) and CC3- neutrophils (orange arrows). (J) Decreased percentage of CC3+ neutrophils (KS, *p* = 0.0193). Scale bars: 50 μm; n ≥5. APAP, acetaminophen (paracetamol); KS, Kolmogorov-Smirnov; KW, Kruskal–Wallis. In all instances: ∗*p* <0.05, ∗∗*p* <0.005.

To further investigate the dichotomous time-dependent role of neutrophils in APAP-ALI, the WT neutrophil response was additionally assessed. Hepatic neutrophil recruitment occurred early in our mouse model and, along with activation, peaked at 16 h post APAP-ALI. Neutrophils were activated following APAP-ALI, with higher CD11b expression and increased CD62L shedding following APAP-ALI (Fig. 2A–G), with this activation continuing for longer than previously observed.^49^ We identified persistently elevated hepatic numbers during repair (Fig. S1E), along with lower circulating neutrophil CD62L (Fig. 2C). Hepatic neutrophils also had lower expression of CC3 following APAP-ALI (Fig. 2H–J), suggesting reduced neutrophil apoptosis resulting from increased neutrophil activation and prolonged survival following APAP-ALI, as seen in other inflammatory conditions.^50^^,^^51^

### AT7519 depletes hepatic neutrophils through CC3-induced apoptosis

APAP-ALI neutrophil survival cues were overcome by AT7519, as shown by marked blood and hepatic neutrophil reduction (Fig. S5A–D). AT7519 is a selective CDK9 inhibitor that downregulates the neutrophil-required survival protein MCL1, which, in turn, results in CC3-mediated apoptosis.^41^^,^^52^ Hepatic CDK9 expression was not affected by AT7519 during APAP-ALI (Fig. S5E), but AT7519 increased the percentage hepatic neutrophil CC3 expression (Fig. S5F and G), a result previously not shown *in vivo*.

### Overexpressing MCL1 does not increase neutrophils in APAP-ALI

Overexpressing MCL1 increases tissue granulocyte numbers by increasing their survival.^41^ Mice expressing *hMcl1*, which decreases neutrophil apoptosis, were used to assess the impact of increased neutrophil numbers on APAP-ALI. Interestingly, there was no difference in hepatic neutrophil number in *hMcl1* mice compared with WT during APAP-ALI (Fig. S5H and I), indicating the MCL1 neutrophil survival pathway is already saturated. Without a difference in neutrophil number, there were no alterations in the hepatic damage phenotype, including no changes in weight loss, serum markers of hepatic damage, or hepatic necrosis (Fig. S5J–L).

### Preventing FPR1 ligation reduces hepatic neutrophil recruitment and activity

**Fig. 3:**
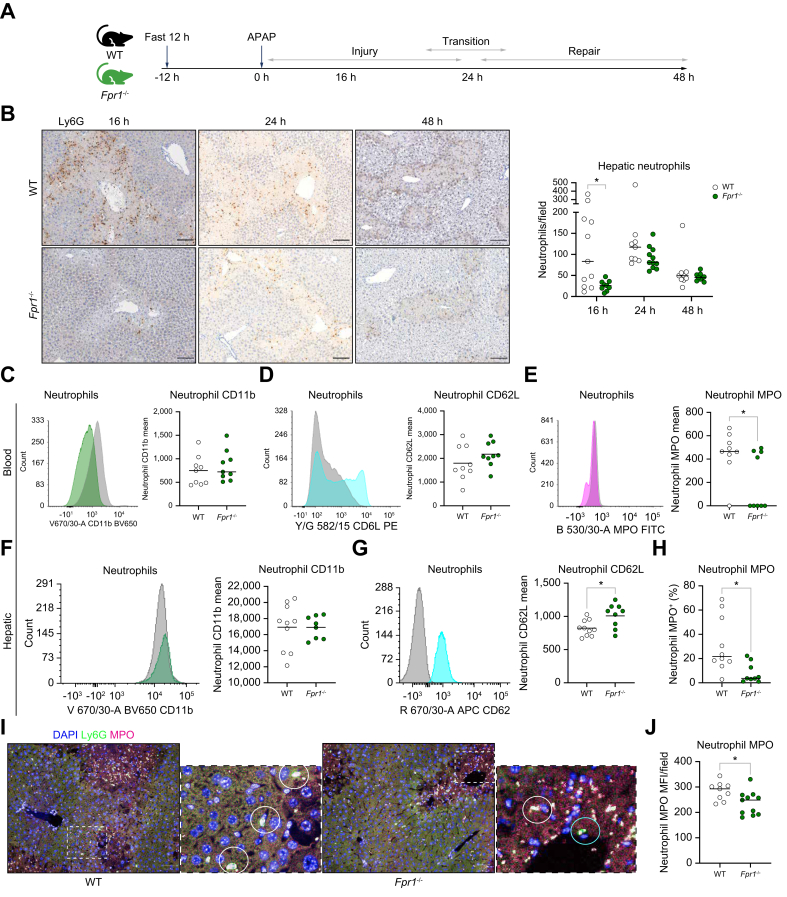
*Fpr1*^*-/-*^ neutrophils are less activated with delayed hepatic recruitment. (A) Model schematic. (B) Representative Ly6G-labeled hepatic sections, showing reduced neutrophil recruitment (KS, *p* = 0.036). (C–E) Representative 16 h blood neutrophil flow cytometry histograms of WT (gray) and *Fpr1*^*-/-*^ (colored) activation markers and quantification, with reduced *Fpr1*^*-/-*^ neutrophil surface MPO (*t* test, WC, *p* = 0.041). (F–H) Hepatic neutrophil activation flow cytometry: (F) CD11b and (G) reduced CD62L shedding (*t* test, *p* = 0.0134), and (H) decreased surface MPO (*t* test, *p* = 0.0258). (I) Representative 24 h Ly6G, and MPO-labeled hepatic sections, showing neutrophils with (white circles) and without (blue circle) surface MPO. (J) Decreased *Fpr1*^*-/-*^ neutrophil MPO (*t* test, *p* = 0.0214). Scale bars: 100 μm (IHC), 50 μm (IF). FPR1, formylated peptide receptor 1; IF, immunofluorescence; IHC, immunohistochemistry; KS, Kolmogorov–Smirnov; MPO, myeloperoxidase; WC, Welch’s correction. In all instances: ∗*p* <0.05.

To further evaluate the novel time-dependent role of neutrophils, neutrophil activation was reduced using a mouse genetic knock out of FPR1 (*Fpr1*^*-/-*^*)*, a key driver of neutrophil activation and migration.^53^^,^^54^ During APAP-ALI, DAMPs, ligands for FPR1, are released, resulting in neutrophil activation and hepatic recruitment.^19^ *Fpr1*^-/-^ mouse neutrophils were not stimulated by formylated peptides *in vitro*, showed no increase in CD11b expression, less shedding of CD62L and a lack of shape change activation compared with WT neutrophils (Fig. S6). Following APAP-ALI *in vivo*, there were fewer circulating *Fpr1*^-/-^ neutrophils and hepatic recruitment was delayed, with lower hepatic numbers during injury compared with WT mice (Fig. 3A,B; Fig. S7A–E). Neutrophil hepatic recruitment was delayed but not prevented, likely as a result of system redundancy and other factors, such as cytokine and DNA binding to neutrophil receptors.[76], [77], [78], [79] As well as being fewer in number, circulating and hepatic *Fpr1*^-/-^ neutrophil activation was reduced during APAP-ALI, evidenced by lower surface myeloperoxidase (MPO) and decreased CD62L shedding (Fig. 3C–J; Fig. S7D).

### Reducing neutrophil activation during APAP-ALI reduces hepatic injury and repair

**Fig. 4:**
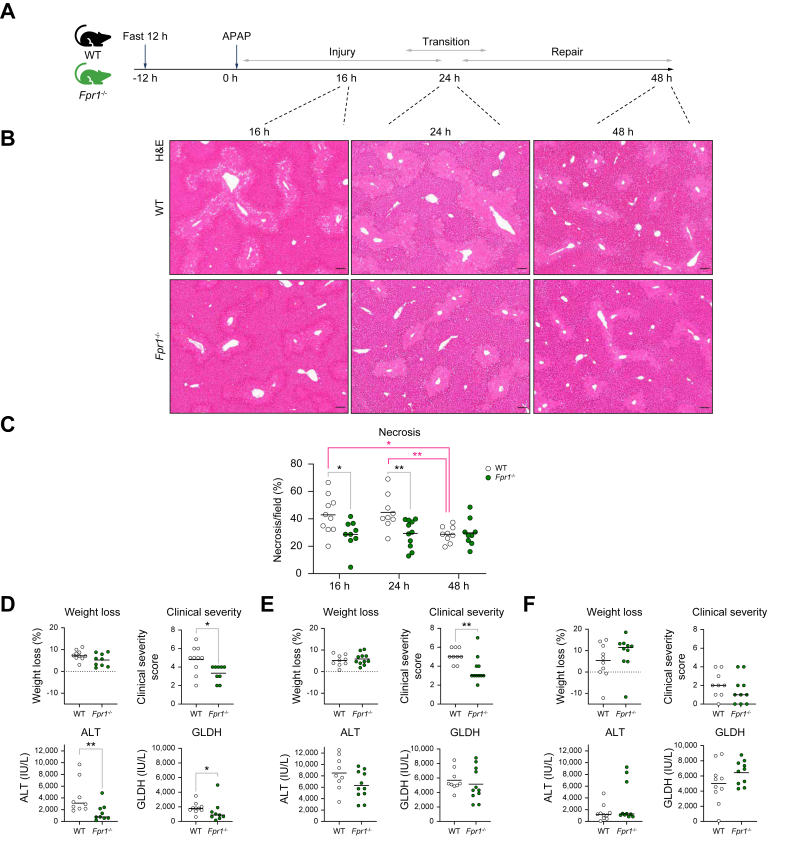
*Fpr1*^-/-^ activated neutrophils contribute to both hepatic injury and repair. (A) Model schematic. (B) Representative H&E hepatic sections showing necrosis. (C) Reduced necrosis in *Fpr1*^*-/-*^ mice (at 16 h; MW, *p* = 0.0279, and 24 h; *t* test, *p* = 0.0072) (black asterisks). Resolved WT hepatic necrosis at 16–48 h (ANOVA and Tukey's test; *p* = 0.0164) and 24–48 h (ANOVA and Tukey's test; *p* = 0.0078) (pink asterisks), not present in *Fpr1*^*-/-*^ mice. (D) Lower clinical severity (*t* test, *p* = 0.018), ALT (MW, *p* = 0.0061), and GLDH (KS, *p* = 0.0258) in *Fpr1*^*-/-*^ mice during injury. (E) Lower 24-h clinical severity (MW, *p* = 0.0052). (F) Weight loss (MW, *p* = 0.2475) and GLDH (*t* test, *p* = 0.1594) at 48 h, during repair; n ≥9. Scale bars: 100 μm. ALT, alanine aminotransferase; FPR1, formylated peptide receptor 1; GLDH, glutamate dehydrogenase; KS, Kolmogorov–Smirnov; MW, Mann-Whitney; WT, wild type. In all instances: ∗*p* <0.05, ∗∗*p* <0.005.

*Fpr1*^-/-^-reduced neutrophil activation was associated with decreased hepatic damage early in APAP-ALI. At 16 h post APAP-ALI, weight loss and clinical severity were lower, along with serum biomarkers of hepatic injury (ALT and GLDH), and hepatic necrosis (Fig. 4A–D). Hepatic necrosis and clinical severity remained decreased 24 h post APAP-ALI (Fig. 4C–E), a time of transition from injury resolution to repair in the WT APAP-ALI model (Fig. S1A–E).

Importantly, there were no differences in hepatic parameters in APAP control-treated (healthy) *Fpr1*^-/-^ and WT mice, including liver:body weight, and hepatic serum markers (ALT, AST, ALP, and GLDH) (Fig. S8A and B). There was also no significant difference in hepatic Cyp2e1 or in baseline neutrophil numbers (Fig. S8D and E).

Hepatic repair was also affected in mice that lacked FPR1-mediated neutrophil activation and recruitment. There was a lack of necrosis area improvement between 24 h and 48 h in *Fpr1*^*-/-*^ mice compared with a 40% reduction in WT mice (Fig. 4B–D). Therefore, both AT7519-mediated neutrophil depletion and preventing FPR1 neutrophil activation were associated with reduced hepatic repair in APAP-ALI. Neutrophil-damaging effects are well documented,^12^^,^^51^ but no studies have yet assessed the effects of neutrophil modulation during both injury and repair. There are several mechanisms by which neutrophils have been implicated in repair,^13^^,^^15^^,^^55^ although this information is limited in APAP-ALI.

### Late neutrophil depletion reduces hepatic proliferation, growth factors, and angiogenesis

All previous studies using neutrophil depletion in APAP-ALI were either initiated before tissue injury or during early injury (≤6 h), even those assessing repair time points.^21^ Inducing neutrophil depletion after the onset of injury allows the time-isolated role of neutrophils in repair to be investigated with greater confidence.

**Fig. 5:**
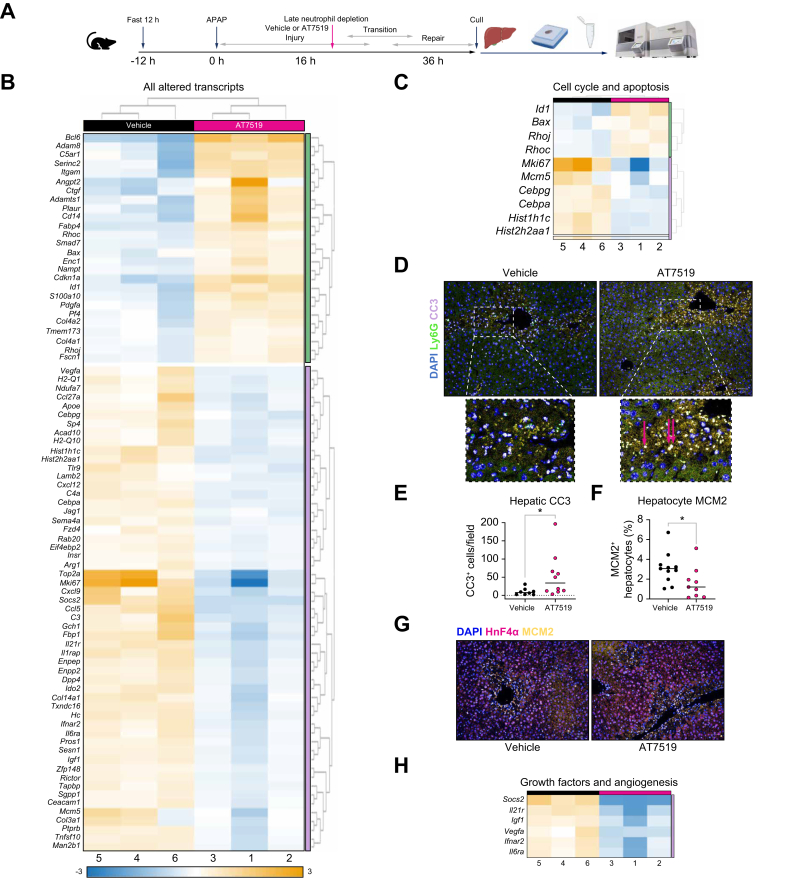
Depleting hepatic neutrophils reduces hepatic proliferation, growth factors and angiogenesis. (A) Experimental schematic. (B) ROSALIND heatmap of 81 transcripts altered between vehicle and AT7519 neutrophil-depleted livers (FC ≥1.25 or ≤-1.25). (C) Cell cycle-related genes showing upregulated cell death transcripts and downregulated proliferation transcripts. (D) Representative CC3 and Ly6G-labeled hepatic sections and magnifications showing increased CC3 following neutrophil depletion (pink arrows), quantified in (E) (MW, *p* = 0.015). (F) Decreased percentage of MCM2+ hepatocytes (MW, *p* = 0.025). (G) Representative MCM2-labeled hepatic sections; n = 10. (H) Reduced hepatic growth factor and angiogenesis-related transcripts after neutrophil depletion; n = 3. Scale bars: 50 μm. FC, fold change; MCM2, minichromosomal maintenance 2; MW, Mann-Whitney. In all instances: ∗*p* <0.05.

Reduced hepatic repair following late neutrophil depletion was further illustrated by elevated hepatic transcripts associated with cell cycle arrest and cell death: (Id1 fold change [FC] 2.139, *p* = 0.0002; Bax FC 1.35, *p* = 0.03) and downregulated cellular proliferation transcripts (Mcm5 FC -1.6, *p* = 0.043; Mik6 FC -6.322, *p* = 0.002) (Fig. 5A–D). This altered transcript expression was complemented by altered protein expression, with increased hepatic CC3 and reduced hepatocyte MCM2 expression, a cellular pre-replication marker (Fig. 5E–G). In addition, following neutrophil depletion, there were decreased transcripts of the hepatic growth factors Socs2 (FC -2.91, *p* = 0.01), ll21r (FC -1.83, *p* = 0.002), and Ifnar2 (FC -1.58, *p* = 0.004), along with Igf1 (FC -1.53, *p* = 0.0016). Transcripts of angiogenic factors, including vascular endothelial growth factor (VEGF) (vegfa FC -1.48, *p* = 0.01) and IL6 (il6ra FC -1.54, *p* = 0.03), associated with tissue repair after APAP-ALI,^56^ were also decreased (Fig. 5H), indicating that neutrophils are involved in hepatic angiogenesis, as seen in other conditions.^16^

### Reduced neutrophil activation is associated with reduced ECM remodeling during repair initiation

**Fig. 6:**
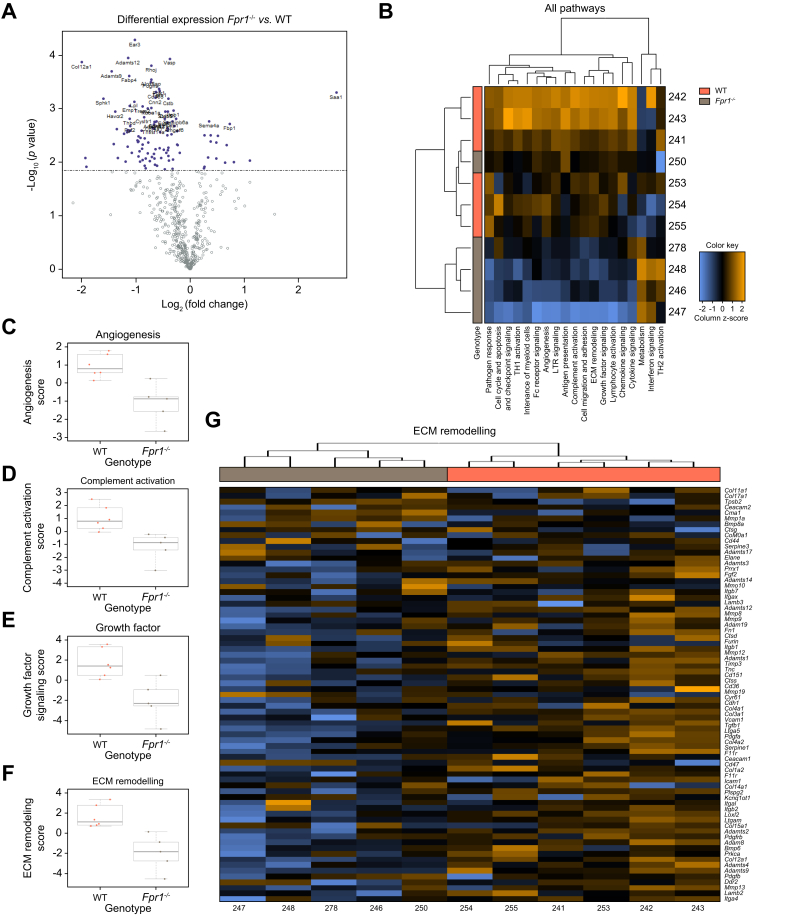
Preventing FPR1 neutrophil activity reduces ECM remodeling. Graphical representations (nSolver) of hepatic NanoString mouse myeloid panel analysis from WT and *Fpr1*^*-/-*^ mice 24 h after APAP administration. (A) Volcano plot highlighting *p* <0.05 results between the two groups (purple data points). (B) Heat map of NanoString nSolver pathway analysis. (C–F) nSolver pathway scores from PCA of each covariate: (C) angiogenesis, (D) complement activation, (E) growth factor signaling, and (F) ECM remodeling. (G) Heat map of ECM-related probes: WT (orange), *Fpr1*^*-/-*^ (gray), and mRNA FC column z score (blue, low; orange, high). Right-hand side shows individual mouse IDs; n ≥5/group. APAP, acetaminophen (paracetamol); ECM, extracellular matrix; FPR1, formylated peptide receptor 1; PCA, principal component analysis; WT, wild type.

At 24 h post APAP-ALI, a transitional time from injury to repair, livers from *Fpr1*^*-/-*^ mice, with fewer and less-activated neutrophils, also had several altered gene transcripts compared with WT controls (Fig. 6A,B). The main reductions were seen in angiogenesis, complement signaling, growth factor signaling, and ECM remodeling (Fig. 6C–F). Some of highest FC parameters were growth factor-related transcripts Fosb (FC -1.91, *p* = 0.012) and Il1r2 (FC -1.93, *p* = 0.0008), which were reduced along with ECM remodeling-related transcripts (Col12a1, FC -1.99 *p* = 0.0001; Adamts9, FC -1.44, *p* = 0.0002; Serpine1 -1.43, *p* = 0.015). Neutrophils contribute to ECM remodeling-promoting repair in other conditions,^57^^,^^58^ but this has not been identified in APAP-ALI. Sphk1 (FC -1.59, *p* = 0.0007), a transcript involved in VEGF signaling,^59^ was similarly reduced (Fig. 6G), demonstrating that analogous repair pathways are affected by both reducing neutrophil activation and depletion in APAP-ALI.

### Reducing neutrophil number and activity alter immune signaling and monocyte phenotype

Neutrophils orchestrate inflammation and subsequent tissue repair through a variety of pathways, including actively communicating with other immune cells, particularly to coordinate recruitment during the initial response to tissue injury.^13^^,^^16^^,^^60^^,^^61^

### Preventing neutrophil FPR1-mediated recruitment reduces monocyte activation during injury

To investigate the impact of reduced neutrophil activation on local inflammation, hepatic NPC populations were assessed by flow cytometry. At 16 h post APAP-ALI, circulating CD45+ cells were not affected by preventing FPR1 neutrophil activation, but hepatic numbers were reduced, as a result, in large part, of reduced neutrophils (Fig. S7A–D). When reducing neutrophil activity, blood and hepatic-infiltrating monocytes were unchanged in number, but hepatic monocytes were less inflammatory, as indicated by higher percentage Ly6C^lo^^38^ (Fig. S9A–F). *Fpr1*^-/-^ mice had higher hepatic f4/80+ macrophages (Fig. S9G), which might be secondary to lower necrosis, given that hepatic macrophage depletion occurs during early APAP-ALI,^62^ and that this difference was resolved at 24 h post APAP-ALI (Fig. S9H). *FPR1* mRNA is highly expressed in neutrophils, specifically hepatic neutrophils following APAP-ALI, supported by analysis of available single cell sequencing data.^63^ We qualified this further and identified protein expression within hepatic Ly6G+ cells following APAP-ALI (Fig. S10). However, both hepatic monocytes and macrophages expressed lower transcript levels (Fig. S10) and, thus, some direct effects on these cells cannot be excluded, despite not identifying protein expression.

### Depleting neutrophils promotes a proinflammatory monocyte phenotype during repair

**Fig. 7:**
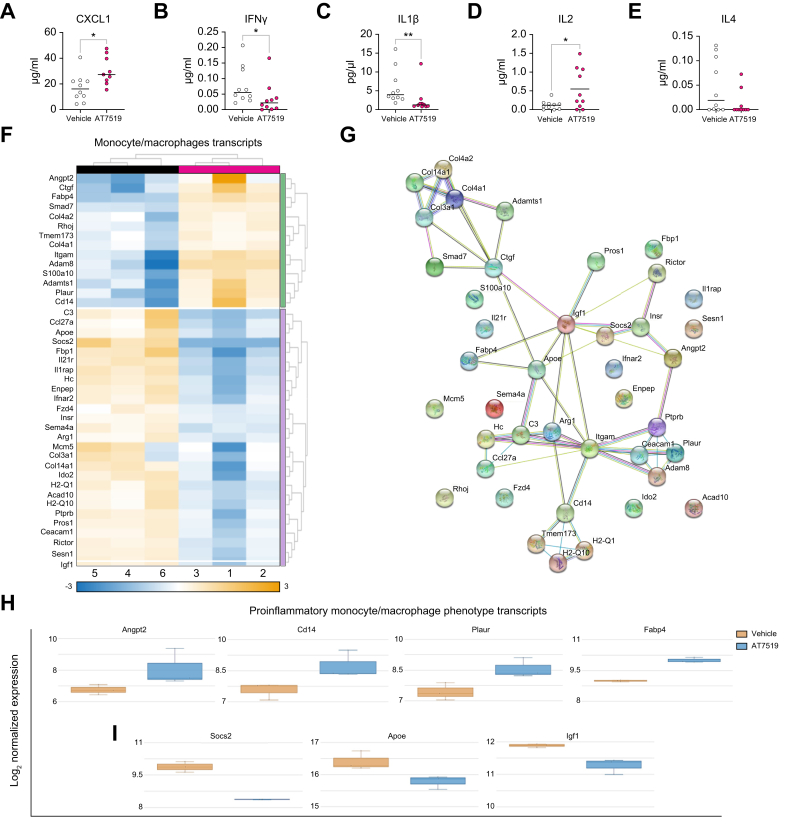
Depleting neutrophils results in a proinflammatory monocyte phenotype during repair. Results from 36-h APAP-ALI mice with AT7519-neutrophil depletion or vehicle control. (A–E) MSD® multiplexed ELISA hepatic tissue cytokine concentrations. Neutrophil depletion (A) increased KCGRO/CXCL1 (MW, *p* = 0.01), and decreased (B) IFNγ (MW, *p* = 0.03), (C) IL1β (KS, *p* = 0.003), (D) IL2 (WC, *p* = 0.034), and (E) IL4 (MW, *p* = 0.062); n = 10 in all instances. (F) ROSALIND heatmap of altered hepatic monocyte/macrophage-related transcripts. (G) NanoString protein known and predicted interactions (nodes represent proteins, edges represent interactions). (H) Upregulated proinflammatory-related transcripts: Angpt2, Cd14, Plaur, and Fabp4; n = 3. (I) Downregulated anti-inflammatory-related transcripts: Socs2, Apoe, and Igf1; n = 3. APAP-ALI, acetaminophen (paracetamol)-induced acute liver injury; CXCL1, C-X-C motif chemokine ligand 1; KS, Kolmogorov–Smirnov; MSD, Meso Scale Discovery; MW, Mann-Witney. In all instances: ∗*p* <0.05, ∗∗*p* <0.005.

Monocyte-derived macrophages can facilitate tissue repair^62^ and neutrophils have been shown to direct a reparative monocyte phenotype *in vitro*, in other models, and, most recently, in APAP-ALI.^64^ We assessed hepatic inflammatory cytokines and monocyte/macrophage phenotypes to investigate how late hepatic neutrophil depletion affected monocyte/macrophage populations and local inflammatory signals (Fig. 7). Depletion resulted in increased C-X-C motif chemokine ligand 1 (CXCL1), a macrophage/monocyte chemoattractant for neutrophils.^65^ There was also higher IL2, which is associated with reduced liver function and recovery in patients with ALI.^66^ IFNγ, a cytokine produced by neutrophils,^15^ was lower, along with IL1β (Fig. 7A–E), a neutrophil elastase-dependent monocyte-produced cytokine.^67^ AT7519 does not deplete monocytes^68^ and we confirmed *in vivo* selectivity with no reduction in blood and hepatic monocytes, hepatic eosinophils, and macrophages (Fig. S11). Following neutrophil depletion, 41 altered monocyte and macrophage-related gene transcripts were detected with NanoString myeloid analysis (Fig. 7F). The corresponding translated proteins showed several interconnections related to an inflammatory phenotype assessed using STRING (v11.0b) (Fig. 7G). Several increased transcripts (Angpt2 [FC 3.23, *p* = 0.008], Cd14 [FC 2.43, *p* = 0.009], and Plaur [FC 2.27, *p* = 0.04]) are associated with a proinflammatory monocyte and macrophage phenotype,^69^^,^^70^ and monocyte/macrophage anti-inflammatory and reparative-associated transcripts (Socs2, Igf1, and Apeo [FC -1.56, *p* = 0.03])^71^^,^^72^ were decreased. Macrophage-produced Fabp4 (FC 2.03, *p* = 0.003), which recruits neutrophils,^73^ was also increased following neutrophil depletion (Fig. 7F–I).

## Discussion

Neutrophil functions and their impact on local hepatic tissue and cellular communications are dependent on the timing of assessment in APAP-ALI. Our results show that they contribute to both hepatic damage in early stages and repair in late stages of this injury, clarifying previous, apparently contradictory, studies.^21^^,^^26^

Our finding of rapid hepatic neutrophil recruitment after APAP-ALI is consistent with other research^21^ and, similar to this report, we did not identify a second wave. There is also no second recruitment wave shown in limited publications detailing human circulating neutrophils over time.^9^^,^^49^ We evidenced persistently increased hepatic numbers and continued activation, consistent with one mouse study documenting elevated neutrophil CD11b at 48 h post injury^49^ and some available late human circulating neutrophil data.^9^^,^^49^ Our WT data highlight some neutrophil differences over time that could be consistent with subsets of these cells. Neutrophil subsets have been defined by individual time point single cell analysis in ALF^63^ and in an APAP autoprotection study.^74^ Neutrophil heterogeneity is clear in various contexts, but distinct subsets remain controversial.[75], [76], [77] We were unable to qualify whether there were discrete time-dependent neutrophil subsets in APAP-ALI or whether recruited neutrophils change *in situ* over time; thus, this requires further investigation, as well as defining its relevance for patients, such as, for example, whether discrete subsets can be targeted.

During injury stages of APAP-ALI, both depleting neutrophils and preventing FPR1-mediated neutrophil activation resulted in diminished hepatic injury. FPR1 ligation is a potent neutrophil activator^18^^,^^20^^,^^54^^,^^78^ and preventing this reduced cell shape change, early chemotaxis, and tissue migration. Preventing FPR1 ligation also reduced degranulation, including MPO, which damages tissue^79^ and contributes to non-alcohol-related steatohepatitis,^80^ but has not previously been associated with APAP-ALI. Hepatic monocytes were also less activated during injury stages, which might be the result of *in situ* neutrophil–monocyte communication or recruitment of an altered monocyte population, given that neutrophils have an essential role in monocyte recruitment and activation, and driving monocyte phenotypes.^61^^,^^81^ To our knowledge, a neutrophil role for monocyte/macrophage cooperation during APAP-ALI damage stages has not previously been demonstrated.

Inducing neutrophil apoptosis early promoted inflammation resolution and reduced tissue damage, as seen in other models.^45^^,^^46^^,^^48^ This was most likely as result of a lack of proinflammatory neutrophil actions, although might have also been secondary to increasing efferocytosis,^41^ given that this promotes an anti-inflammatory macrophage phenotype,^82^ which is important in APAP-ALI.^83^

Based on injury time point results alone, reducing neutrophil recruitment, activation, and subsequent hepatic damage would appear a potentially tractable therapeutic target in APAP-ALI, particularly with the horizon of more available specific FPR1 inhibitors and clinical trials in other conditions.^84^^,^^85^ Indeed, a positive effect has been seen during early APAP-ALI with pharmacological inhibition,^20^ and in other conditions.^17^^,^^18^^,^^43^^,^^53^ Patients with APAP-ALI have high circulating numbers of activated neutrophils,^5^^,^^6^ but the positive effect of reducing early neutrophil activity could have detrimental effects for tissue repair or altered sepsis risk during patient recovery.^8^^,^^9^ Given that *N*-acetylcysteine (NAC) is the standard of care in human medicine,^3^ the impact of reducing secondary inflammation with specific FPR1 inhibitors would first need to be assessed alongside NAC, including interrogation of the subsequent repair. Information from prospective longitudinal neutrophil functional studies in humans with APAP-ALI, alongside results of clinical trials in patients with inflammatory bowel disease (EudraCT Number: 2021-000035-31.23), would be important information to incorporate to propose an optimal therapeutic time point for patients. It is also more crucial to identify late presentation treatment options, given that these are currently limited, for example focusing on harnessing or recovering neutrophil reparative and protective functions.

Hepatic neutrophils were increased and remained activated during repair, for longer than previously identified.^45^^,^[66], [67], [68] A tissue requirement for neutrophils during APAP-ALI repair after their depletion is highlighted here by increased hepatic CXCL1 and Fabp4, which are both are secreted by macrophages to drive neutrophil recruitment.^65^^,^^73^^,^^86^ Hepatocytes also produce CXCL1 to recruit neutrophils, secondary to macrophage TNF signaling.^87^ These results are consistent with findings reported by Chuahan *et al.*,^88^ which ascertained that hepatic macrophage TNF secretion is important for neutrophil recruitment and subsequent hepatic repair in APAP-ALI. Interestingly, TNF also increases expression of FPR1 on human neutrophils.^88^

FPR1-mediated neutrophil actions are required for APAP-ALI repair, demonstrated by the lack of necrosis improvement at 48 h post injury with reduced neutrophil activity and reduced ECM-related transcripts. Neutrophil-mediated wound healing through ECM remodeling has been demonstrated in skin and peritoneum^57^^,^^58^ and FPR neutrophil activation has been shown to accelerate healing.^89^ Evidencing altered ECM-related transcripts both following neutrophil depletion, and when preventing FRPR1-mediated neutrophil functions, signifies their contribution to ECM remodeling in APAP-ALI and additional mechanistic investigations are warranted.

FPR1 expression is predominantly neutrophilic,^85^ which increases with activation^18^ and is a key regulator of hepatic recruitment during injury, as shown here and in other models.^53^^,^^90^ To our knowledge, hepatic cellular protein expression has not been interrogated and not specifically in APAP-ALI. We showed highest FPR1 expression and localization on hepatic neutrophils, with no parenchymal labeling. Human Protein Atlas datasets show a predominant expression on neutrophils (Tissue Cell Type – FPR1 – The Human Protein Atlas) and our analysis of a mouse APAP-ALF single cell sequencing dataset^63^ confirmed highest fold neutrophil expression, but also lower positivity in hepatic monocytes. We recognize that FPR1 is not limited to neutrophils, but, as in other inflamed tissues, the impact on other immune cells and, therefore, their phenotype in the context of APAP-ALI is considered to be small. Given that we also identified a striking effect on monocyte/macrophage phenotype with AT7519-mediated neutrophil depletion, we consider our comparable *Fpr1*^*-/-*^ results to be neutrophil mediated. Other FPRs were not investigated in our study, because the genetic modulation was FPR1 specific. FPR2 is reported to improve inflammation resolution and is recognized on neutrophils;^91^^,^^92^ furthermore, mitochondrial formyl peptides can bind FPR2 with similar affinity,^93^ and, therefore, binding might have contributed to restored hepatic neutrophil accumulation at 24 and 48 h post APAP. Neutrophil FPR2-mediated functions could be of interest in future studies of APAP-ALI.

Neutrophil-mediated angiogenesis is also important for tissue repair,^15^^,^^16^ and we identified reductions in angiogenesis transcripts with both methods of neutrophil modulation. Hepatic Sphk1, which regulates angiogenesis through reactive oxygen species (ROS)^59^ and is shown to be proinflammatory during early APAP-ALI,^94^ is reduced without FPR1-activated neutrophils. VEGF produced by neutrophils, and macrophages following neutrophil communication,^16^^,^^95^ was reduced without neutrophils, and is important for hepatic regeneration.^56^

We also revealed both damaging and reparative neutrophil functions in APAP-ALI, resolving apparent contradictory studies. Similar controversies once existed for monocytes and macrophages in APAP-ALI, but subpopulations are now accepted, along with divergent time-dependent roles of infiltrating monocyte-derived macrophages in APAP-ALI,^96^^,^^97^ and their reparative functions have since been harnessed to improve APAP-ALI repair.^98^ Elucidating and harnessing neutrophil repair functions to treat patients with APAP-ALI, particularly those found to have abnormal neutrophil functions,^9^ is an important future direction.

Late neutrophil depletion was also associated with proinflammatory monocyte/macrophage phenotype transcripts, indicating at least part of the neutrophil reparative function occurs through their positive communications with monocytes and macrophages. These findings complement those of Yang *et al.*,^28^ who identified a lack of monocyte/macrophage conversion to a pro-reparative Ly6C^lo^CX3CR1^hi^ phenotype without neutrophil ROS.

Given our results, the timing of neutrophil interventions and assessments is vital. The rapid, robust, and selective pharmacological depletion of hepatic neutrophils revealed in this study, even at peak inflammation, facilitated interrogation of their impact at different times. Importantly, without the effect of any proceeding modulations, such as those seen with nonspecific depletions^29^^,^^99^ or before injury modulations.^21^^,^^30^ AT7519 does not deplete other innate immune cells and does not reduce macrophage numbers,^41^^,^^68^^,^^100^ although reduced proinflammatory macrophage cytokine expression has been reported,^68^ which could have contributed to the reduced injury results.

We established that multiple reparative hepatic pathways are affected by reducing neutrophils or their functions; however, there are potentially further neutrophil roles that we have not yet explored. These include phagocytosis of necrotic cellular debris,^10^ miRNA production,^101^^,^^102^ and infection control, particularly given the reduced neutrophil functions of patients with APAP-ALI-induced ALF^8^^,^^9^ and the higher risk of fatal bacterial infections.^3^

There is limited information about neutrophil activity and function in patients with APAP-ALI. Unfortunately, given the severity of patient illness, and often coagulation complications, hepatic samples are frequently limited to post-transplanted livers, significantly biasing analysis to non-recovery groups. Therefore, circulating neutrophils are the next proxy of measurement in humans. Patients with APAP-ALI have increased circulating neutrophil numbers similar to our mouse model, which normalize by Day 4 of recovery, although no difference between surviving and non-surviving cohorts has been found.^6^ Circulating neutrophil CD64, the Fcγ receptor and a marker of neutrophil activation, is elevated in patients with APAP-ALI,^23^ but given that this also increases with sepsis, interpretation of functional significance is challenging. Williams *et al.* reported results from three patients recovering from APAP-ALI, detailing elevations in circulating neutrophil ROS, CD11b, and phagocytic capacity during recovery.^74^ Cytopenia is a negative risk factor in APAP-ALI^22^ and reduced neutrophil activity and phagocytic function were negatively corelated with survival in a small cohort of patients with APAP-ALI.^9^ The sparsity of information regarding neutrophil kinetics, maturity, granularity, transcriptional activity, and function in APAP-ALI likely highlights the difficulties associated with capturing patient samples and rapidly analyzing these terminally differentiated cells.

Depicting this duality and time-dependent function of neutrophils in APAP-ALI indicates that, rather than previous studies being contradictory, both lines of investigation are true; neutrophils contribute to both hepatic injury and later resolution and repair. Further investigations to determine pivotal neutrophil functions and specifically reparative subsets, and those promoting known reparative macrophage phenotypes^103^ in APAP-ALI are important avenues of continued investigation. Large prospective longitudinal studies with rapid on-site analysis are required to interrogate circulating neutrophil phenotypes and functions in patients. Such studies could establish further neutrophil prognostic biomarkers and potentially define patients requiring intervention, such reducing early neutrophil-damaging functions or rescue of late repair and phagocytic functions.

We do know that circulating neutrophils differ significantly from tissue neutrophils;^104^ thus, continued investigations in the mouse model, which provides information otherwise inaccessible in humans,^105^ are still required. For example, as mentioned, such work could involve trialing more specific FPR1 inhibitors alongside NAC, as well as further defining neutrophil monocyte cooperation.

Further investigation of this now known time-dependent role in APAP-ALI could not only facilitate development of much needed novel late presentation treatments for this condition, but could also facilitate treatments in other conditions.

## Abbreviations

ALF, acute liver failure; ALI, acute liver injury; ALP alkaline phosphatase; ALT, alanine aminotransferase; APAP-ALI, paracetamol-induced acute liver injury; APAP, acetaminophen (paracetamol); AST, aspartate aminotransferase; CC3, cleaved caspase 3; CDKI, cyclin-dependent kinase inhibitor; CV, central vein; CXCL, C-X-C motif chemokine ligand; Cyp2e1, cytochrome P450 2E1; DAB, 3,3′-diaminobenzidine; DAMP, damage-associated molecular pattern; ECM, extracellular matrix; EGTA, ethylene glycol-bis(β-aminoethyl ether)-N,N,N′,N′-tetraacetic acid); ES, effect size; FACS, fluorescence-activated cell sorting; FC, fold change; FCS, fetal calf serum; FFPE, formalin-fixed paraffin-embedded; FPR1, formylated peptide receptor 1; GLDH, glutamate dehydrogenase; HBSS, Hank’s buffered salt solution; KS, Kolmogorov–Smirnov; LC, liquid chromatography; MBP, major basic protein; MCL1, myeloid cell leukemia factor 1; MCM, minichromosomal maintenance; MPO, myeloperoxidase; MS, mass spectrometry; MSD, Meso Scale Discovery; MW, Mann-Whitney; NAC, N-acetylcysteine; NPC, nonparenchymal cells; PAF, platelet-activating factor; PBST, PBS 0.1% Tween 20; PCA, principal component analysis; PFA, paraformaldehyde; RBC, red blood cell; ROS, reactive oxygen species; RT, room temperature; RT, room temperature; VEGF, vascular endothelial growth factor; WC, Welch’s correction; WT, wild type.

## Financial support

This study received the following financial support: Wellcome Trust [108906/Z/15/Z] (JAC), 10.13039/501100000265Medical Research Council (MRC) UK grant MR/K013386/1 (AR), MRC Autologous Macrophage Therapy for liver cirrhosis (DPFS grant) MR/M007588/1 (RA), MRC Defining The Regenerative Capacity Of Ductular Cells From Non-transplantable Human Liver grant MR/P016839/1 (TYK), MRC Macrophage Therapy for Acute Liver Failure (MAIL) grant MR/T044802/1 (MA, MC), MRC UKRMP Exploiting In Silico Modelling to Address the Translational Bottleneck in Regenerative Medicine Safety grant MR/T015489/1 (CAH), and MRC Research Grant MR/X019314/1 (CDL).

## Authors’ contributions

Conceptualization: JAC, AGR, SJF. Methodology: JAC, AGR, SJF, LC, PSL (statistics; JAC, AGR). Investigation: JAC, PMDP, EL, NG, MO, GR, JPS, NZH, RA, TYM, MA, CAH, MC, AJF, CTR, AMK, MV. Visualization: JAC, EL, NZH, PSL, LC, CDL, DAD, AGF, SJF. Funding acquisition: JAC, AGR, SJF. Project administration: JAC. Supervision: AGR, SJF. Writing – original draft: JAC. Writing – review & editing: JAC, PMDP, LC, PSL, AGR, SJF. All authors have read and agreed to the published version of the manuscript.

## Data availability statement

All data, code, and materials used in the analysis are available upon request. The NanoString datasets generated and/or analyzed during the current study are available in the Edinburgh DataShare repository (https://doi.org/10.7488/ds/7835 and https://doi.org/10.7488/ds/7836), and mass spectrometry data are available at https://doi.org/10.7488/ds/7686. Additional datasets are also available from the corresponding author on reasonable request. Requests for materials should be sent to the corresponding author.

## Conflicts of interest

There was no competing interests at the time of the experiments; however, LC, PSL, and SJF are shareholders of Resolution Therapeutics Ltd. a macrophage cell therapy developer. SJF is a scientific adviser for, and LC is an employee of, Resolution Therapeutics. AMK is a consultant for Resolution Therapeutics.

Please refer to the accompanying ICMJE disclosure forms for further details.

## Supplementary data

[Multimedia component 1] Multimedia component 1

[Multimedia component 2] Multimedia component 2

[Multimedia component 3] Multimedia component 3

[Multimedia component 4] Multimedia component 4
