# Supplementary material for: Temporal dichotomy of neutrophil function in acute liver injury and repair
Source: JHEP Rep. 2025 Apr 11;7(7):101417. doi: 10.1016/j.jhepr.2025.101417 (PMC12213964; doi:10.1016/j.jhepr.2025.101417)
Supplement: [Multimedia component 1] [file mmc1.pdf]

Temporal dichotomy of neutrophil function in acute liver injury and repair

Jennifer A. Cartwright, Philippe M.D. Potey, Eilidh Livingstone, Lara Campana, Philip J. Starkey Lewis, Magdalena E.M. Oremek, Naomi N. Gachanja, Giulia Rinaldi, Rhona E. Aird, Tak Yung Man, Anuruddika J. Fernando, Joanna P. Simpson, Natalie Z.M. Homer, Nicole Barth, Melisande Addison, Candice Ashmore-Harris, Maria Elena Candela, Alastair M. Kilpatrick, Matthieu Vermeren, Calum T. Robb, David A. Dorward, Christopher D. Lucas, Stuart J. Forbes, Adriano G. Rossi

Table of contents

Supplementary figures……………………………………………………………….…....2

Supplementary methods…………………………………………………………….....18

Supplementary tables……………………………………………..…………………18

Supplementary references………………………….……………………………….23

Supplementary figures

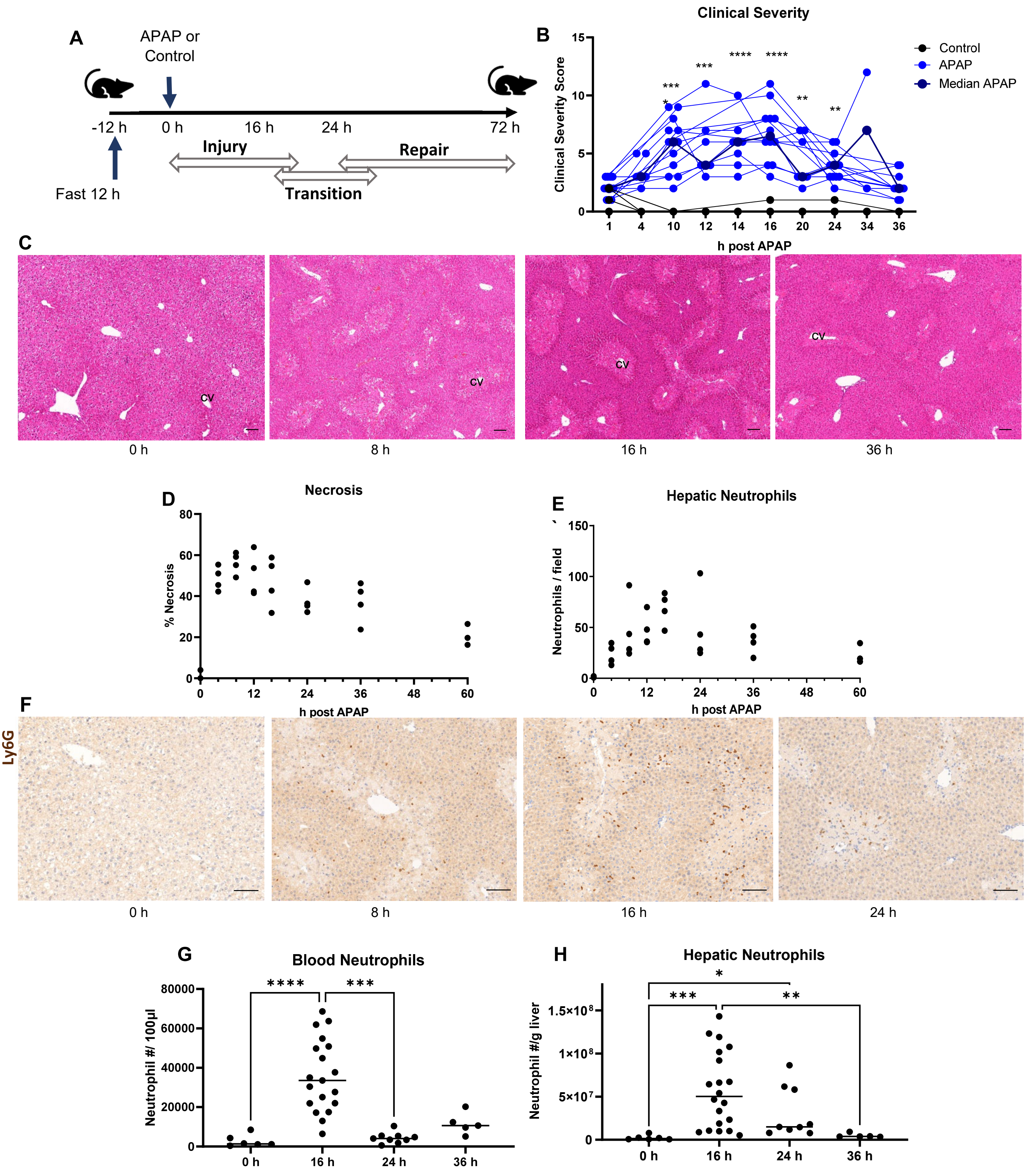

Fig. S1. Hepatic neutrophil accumulation peaks at 16 h in the WT mouse model. (A), Model schematic. **(B),** Total clinical severity score. **(C),** Representative HnE hepatic sections showing necrotic areas around central veins (cv). (**D),** Quantified average % hepatic necrosis. **I,** Average number of neutrophils/hepatic field, quantified from **(F),** Ly6G+ DAB labelled sections, representative images shown. **(G),** Blood neutrophils

significantly different over time, KW $p<0.0001$, Dunn's, 0 v 16, $p=0.0001$, 16 v 24, $p=0.0001$. **(H),** Hepatic neutrophils significantly different over time, KW $p<0.0001$, highest at 16 h post APAP Dunn's, 0 v 16, $p=0.0001$, 0 v 24, $p=0.0256$, 16 v 36, $p=0.0096$. Scale bars 100 µm. KW Kruskal Wallis. n ≥4/group.

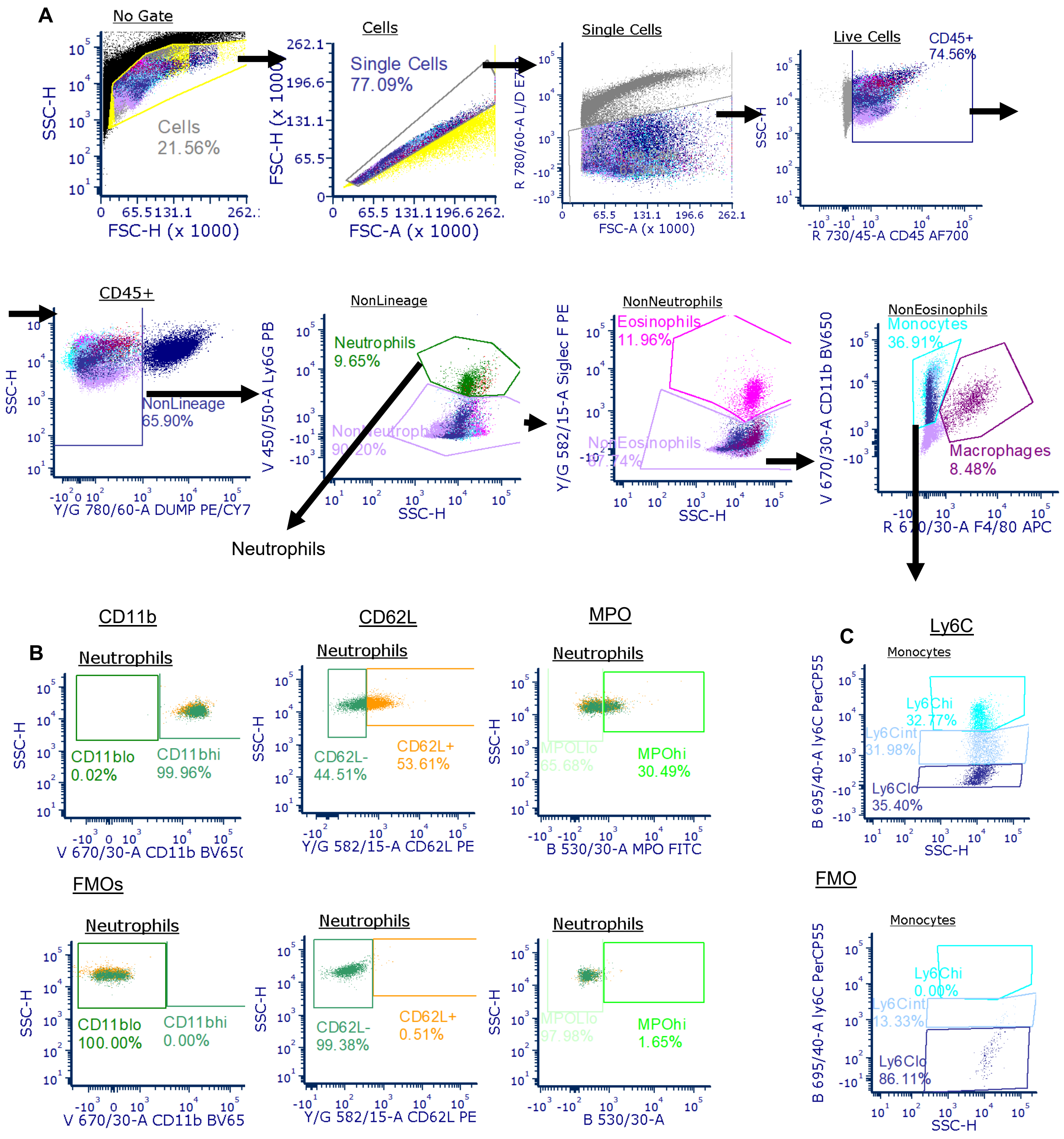

Fig. S2. Hepatic flow cytometry gating strategy for innate immune cells. (A), Representative flow plots of hepatic NPCs demonstrating gating strategy. Each plot is labelled with the parent gate and showing cell populations and gates of interest. Each dot is colored with final gate of interest., e.g. neutrophils are green and Ly6C^{hi} monocytes are cyan. (**B),** Example neutrophil activation markers and corresponding fluorescence minus one control (FMO) below. **(C),** Monocyte Ly6C expression gating and corresponding FMO below.

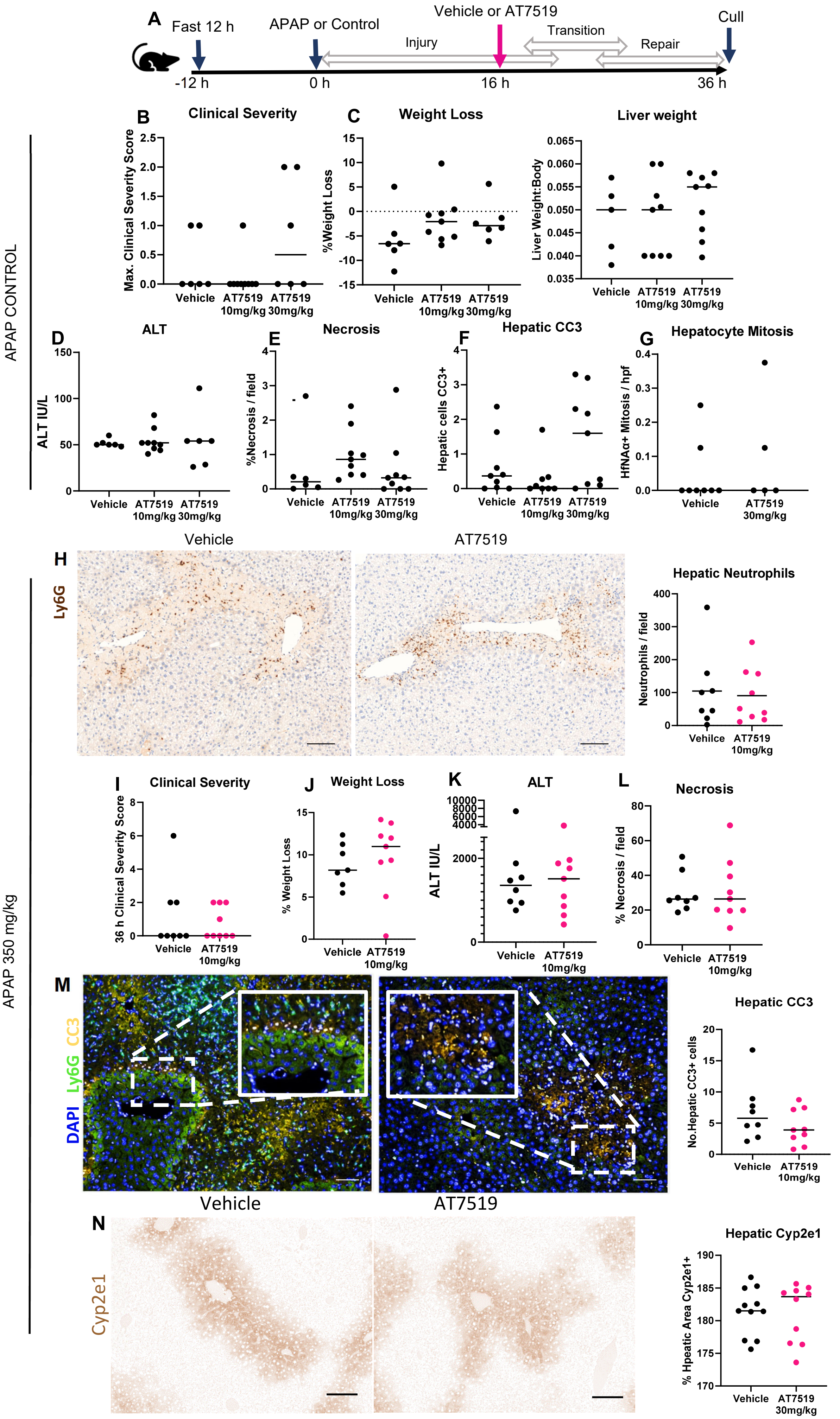
A
Fast 12 h
APAP or Control
Vehicle or AT7519
Cull
Injury
Transition
Repair
-12 h
0 h
16 h
36 h
APAP CONTROL
B
Clinical Severity
Max. Clinical Severity Score
C
Weight Loss
%Weight Loss
Liver weight
Liver Weight:Body
Vehicle
AT7519 10mg/kg
AT7519 30mg/kg
D
ALT
ALT IU/L
E
Necrosis
%Necrosis / field
F
Hepatic CC3
Hepatic cells CC3+
G
Hepatocyte Mitosis
HfNAα+ Mitosis / hpf
H
Vehicle
AT7519
Ly6G
Hepatic Neutrophils
Neutrophils / field
Vehilce
APAP 350 mg/kg
I
Clinical Severity
36 h Clinical Severity Score
J
Weight Loss
% Weight Loss
K
ALT
ALT IU/L
L
Necrosis
% Necrosis / field
M
DAPI
Ly6G
CC3
Hepatic CC3
No.Hepatic CC3+ cells
N
Cyp2e1
Hepatic Cyp2e1
% Hpeatic Area Cyp2e1+

Fig. S3. AT7519 does not affect healthy livers, and neutrophil depletion is dose dependent

(A), Model schematic. **(B-G),** APAP control treated mice, with no difference in **B,** clinical severity, **C,** % weight loss and liver: body weight following 10 mg/kg or 30 mg/kg AT7519. **D,** Serum ALT, **E,** % hepatic necrosis, **F,** % of hepatic cells positive for CC3 and **G,** Hepatocyte (DAPI+HNF4α+) mitotic events / field also unaltered. **(H-M),** APAP treated mice received vehicle or AT7519 10 mg/kg **H,** Neutrophil labelled hepatic sections and quantification showed no reduction. **I,** 36 h clinical severity, **J,** % weight loss, **K,** Serum ALT, and **L,** % hepatic necrosis unaltered without neutrophil depletion. **M,** Representative hepatic CC3 labelled sections and quantification showing no increase in hepatic CC3. **N,** Representative Cyp2e1 labelled hepatic sections from APAP treated mice with vehicle or 30 mg/kg AT7519, and quantification. IHC scale bars 100 μm. IF scale bars 50 μm. (n≥6).

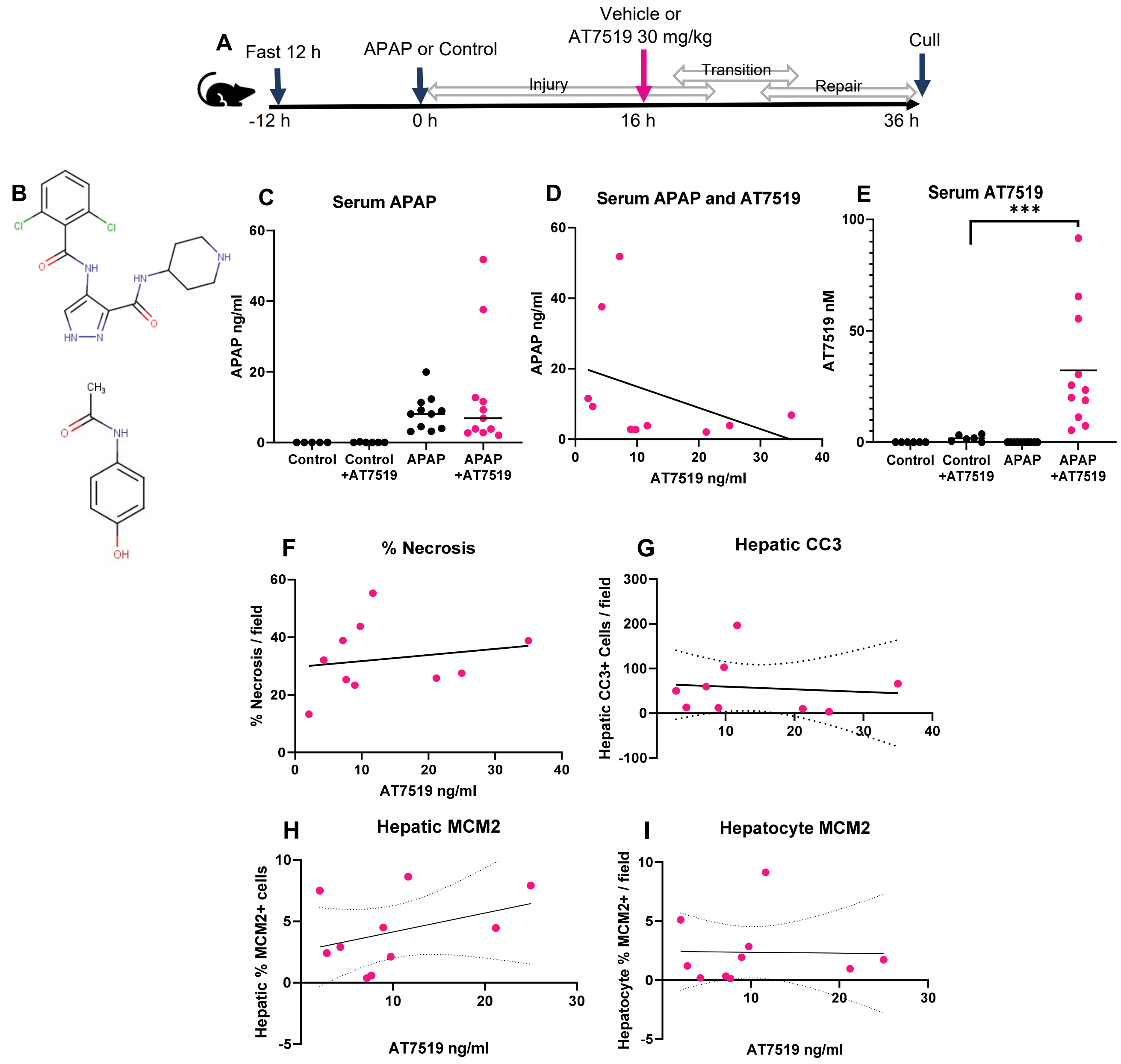

Fig. S4. Serum AT7519 concentrations do not correlate with injury or repair

(A), Model schematic. **(B),** Structure of AT7519 and APAP. **(C),** Serum APAP concentrations not increased with AT7519. **(D),** No correlation of serum AT7519 and APAP concentrations, R^2=0.007. **(E),** Serum AT7519 concentration increased following APAP-ALI, KS, p=0.0002. **(F-H),** Linear regression analysis of mouse serum AT7519 concentrations showed no correlation with; **F,** Average % necrosis R^2=0.2721, **G,** Average number of hepatic CC3+ cells R^2=0.1349, and **H,** Hepatic proliferation % MCM2 in all cells R^2=0.1508, and

I, % hepatocyte MCM2 expression R^2=0.00004. (n≥9/APAP groups). MCM2 minichromosome maintenance complex component 2, CC3 cleaved caspase 3. KS, Kolmogorov-Smirnov.

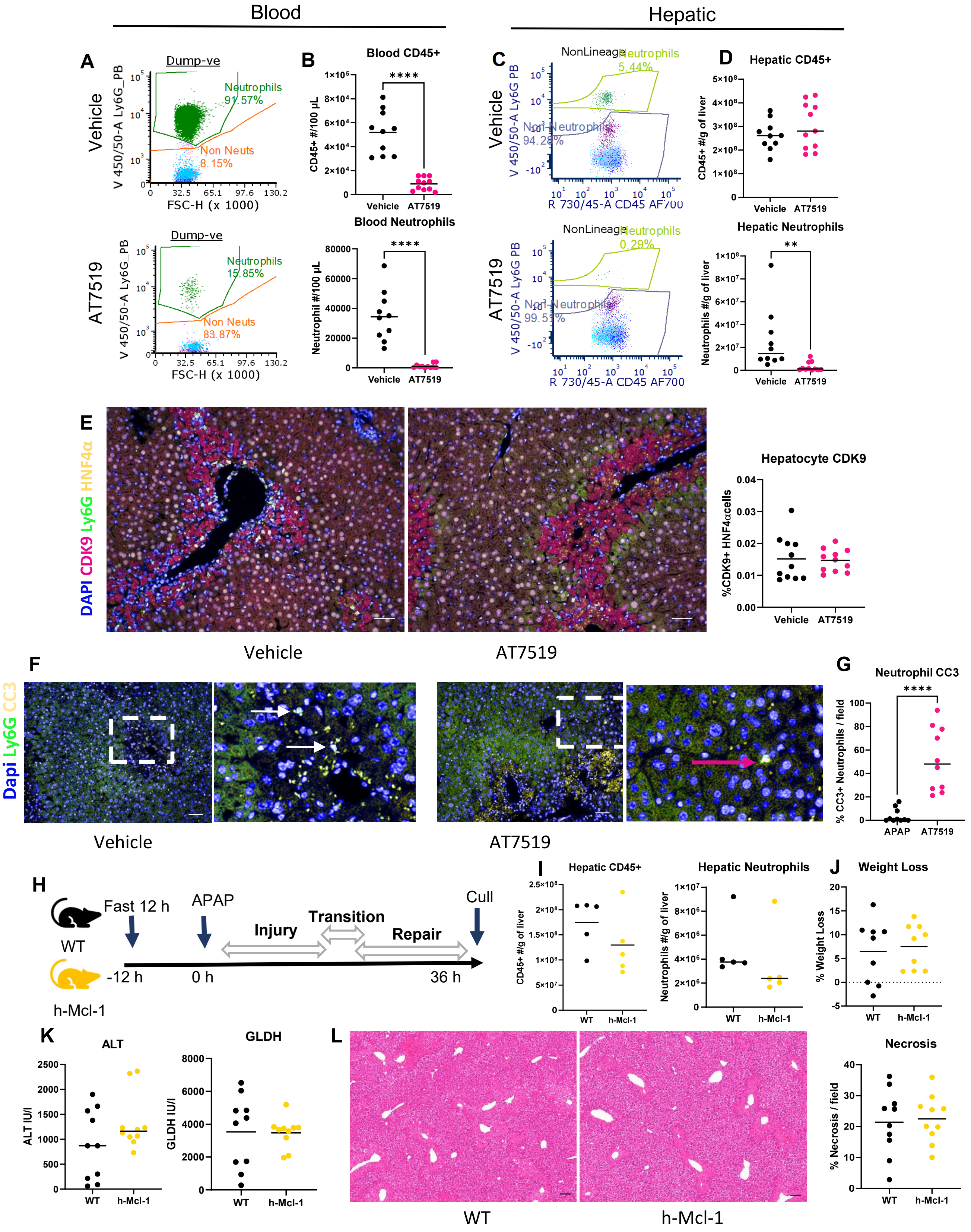

Fig. S5. AT7519 depletes hepatic neutrophils through CC3 and neutrophil survival is saturated

(A-G), 30 mg/kg AT7519 Neutrophil depletion ($n \geq 10$), assessed 16 h. **A,** Representative blood flow plots, **B,** AT7519 reduced circulating CD45+ cells, t-test, WC, $P<0.0001$, and neutrophils (CD45+Lin-Ly6G+) KS, $P<0.0001$. **C,** Representative hepatic flow plots **D,** CD45+ cells unaltered but reduced neutrophils KS, $p=0.0021$. **E,** Representative HNF4α, Ly6G and CDK9 labelled hepatic sections, and CDK9 quantification. **F,** Representative Ly6G and CC3 labelled hepatic sections. Magnified panels show increased CC3+ neutrophils (pink arrow) after AT7519. **G,** Percentage neutrophils CC3+, KS, $p<0.0001$. (**H**)**,** Schematic, 350 mg/kg APAP treated WT and h-Mcl-1 mice, assessed 36 h. **(I),** Hepatic NPC flow cytometry; CD45+ cells and neutrophils not increased. **(J),** % weight loss, and **(K),** unaltered ALT and GLDH **(L),** Representative hepatic sections showing unaltered hepatic necrosis. KS, Kolmogorov-Smirnov. IHC scale bars 100 μm. IF scale bars 50 μm.

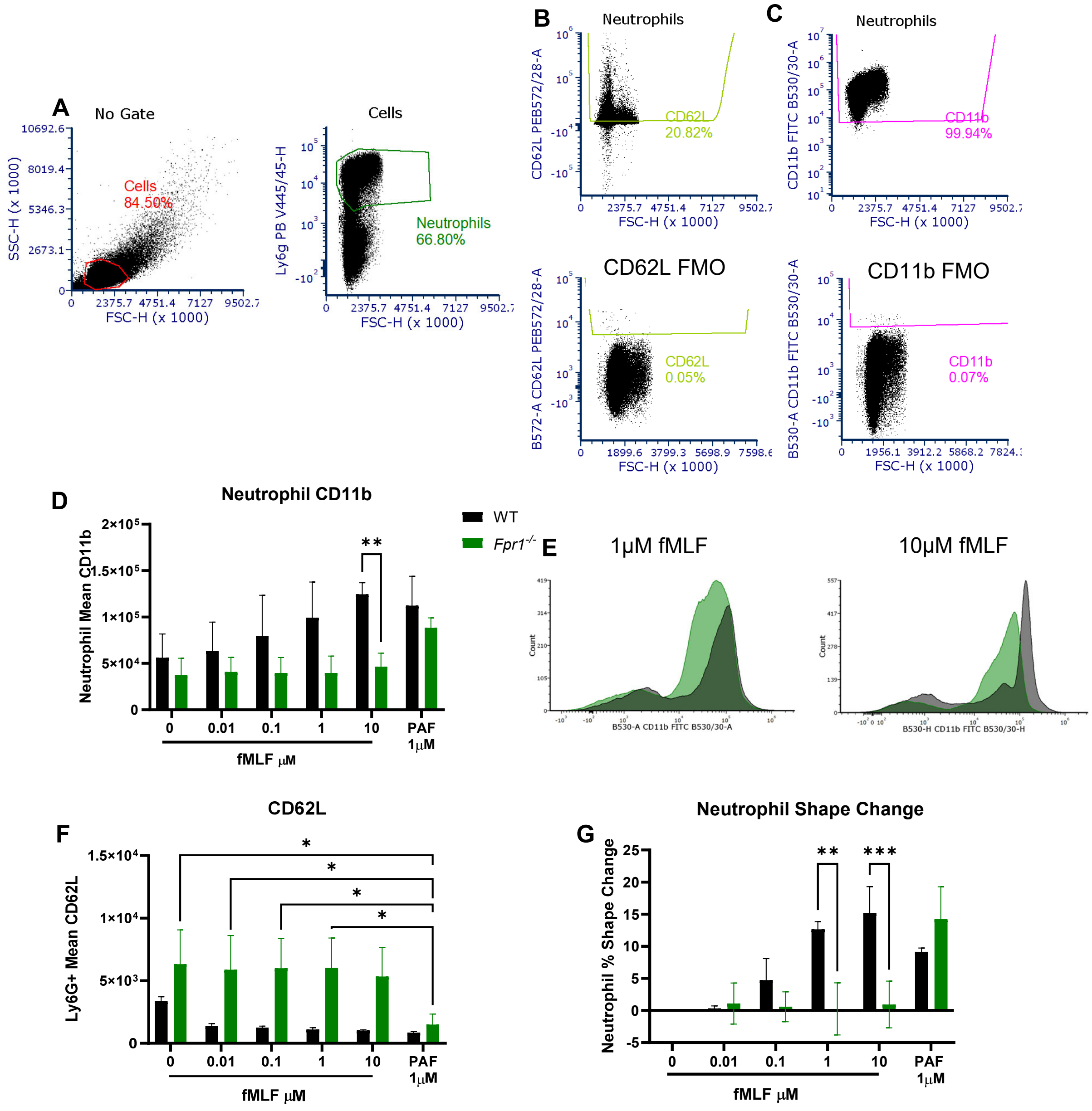

Fig. S6. ***Fpr1**$^{-/-}$* **neutrophils are not activated by formylated peptides**

(A), Bone marrow derived mouse neutrophil flow cytometry gating. **(B),** CD62L+ and FMO. (**C),** CD11b+ and FMO. **(D),** FMLF did not increase CD11b expression compared to WT, ANOVA, p=0.0335, Ší'ák's, p=0.0022 (10 μm FMLF). **(E),** Representative CD11b expression histogram (grey WT, green *Fpr1*$^{-/-}$). **(F),** *Fpr1*$^{-/-}$ neutrophils did not shed CD62L compared to positive control PAF, unlike WT neutrophils (0.01 Mm FMLF vs. PAF 1 μM, p= 0.0348, 0.1 Mm FMLF vs. PAF 1 μM, p= 0.0288, 1 Mm FMLF vs. PAF 1 μM, p= 0.0268, ANOVA with Tukeys). **(G)** *Fpr1*$^{-/-}$ neutrophils lower % shape change (FSC-H) than WT ANOVA, WT

vs KO p=0.0011, Ší'ák's, p=0.0014 (1 µm FMLF), p=0.0004 (10 µm FMLF). (n =3). PAF platelet activating factoR, FMLF N-Formylmethionyl-leucyl-phenylalanine.

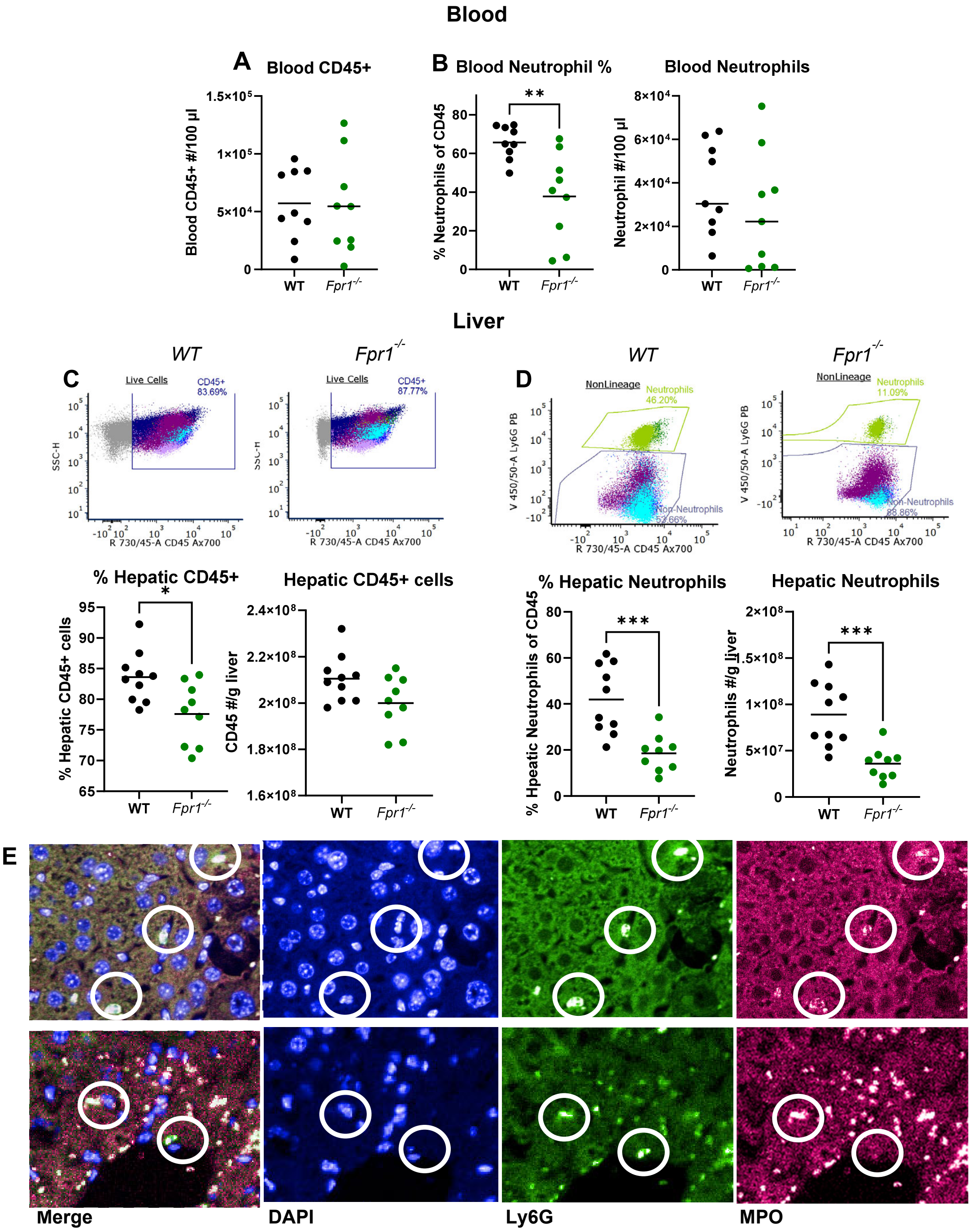

Fig. S7. Hepatic *Fpr1*$^{-/-}$ neutrophils are fewer and less activated following APAP-ALI

(A-D), Blood and hepatic NPC flow cytometry WT and *Fpr1*$^{-/-}$ mice 16 h after APAP. **A,** Circulating CD45+ unaltered. **B,** Circulating % neutrophils of CD45+ reduced (t-test, WC, p=0.006). **C,** Representative flow plots, hepatic CD45+% reduced significantly (t-test, p=0.01). **D,** Representative flow plots of hepatic neutrophils and quantification. Hepatic neutrophil % of CD45+ and absolute number reduced in *Fpr1*$^{-/-}$ mice (t-test,

p=0.0006). (**E),** Representative magnified hepatic sections individual channels from Figure 3, WT and *Fpr1*$^{-/-}$ mice 24 h after 350 mg/kg APAP i.p. Each data point represents an individual mouse (n≥9). WC Welch's correction. IF scale bars 50 µm.

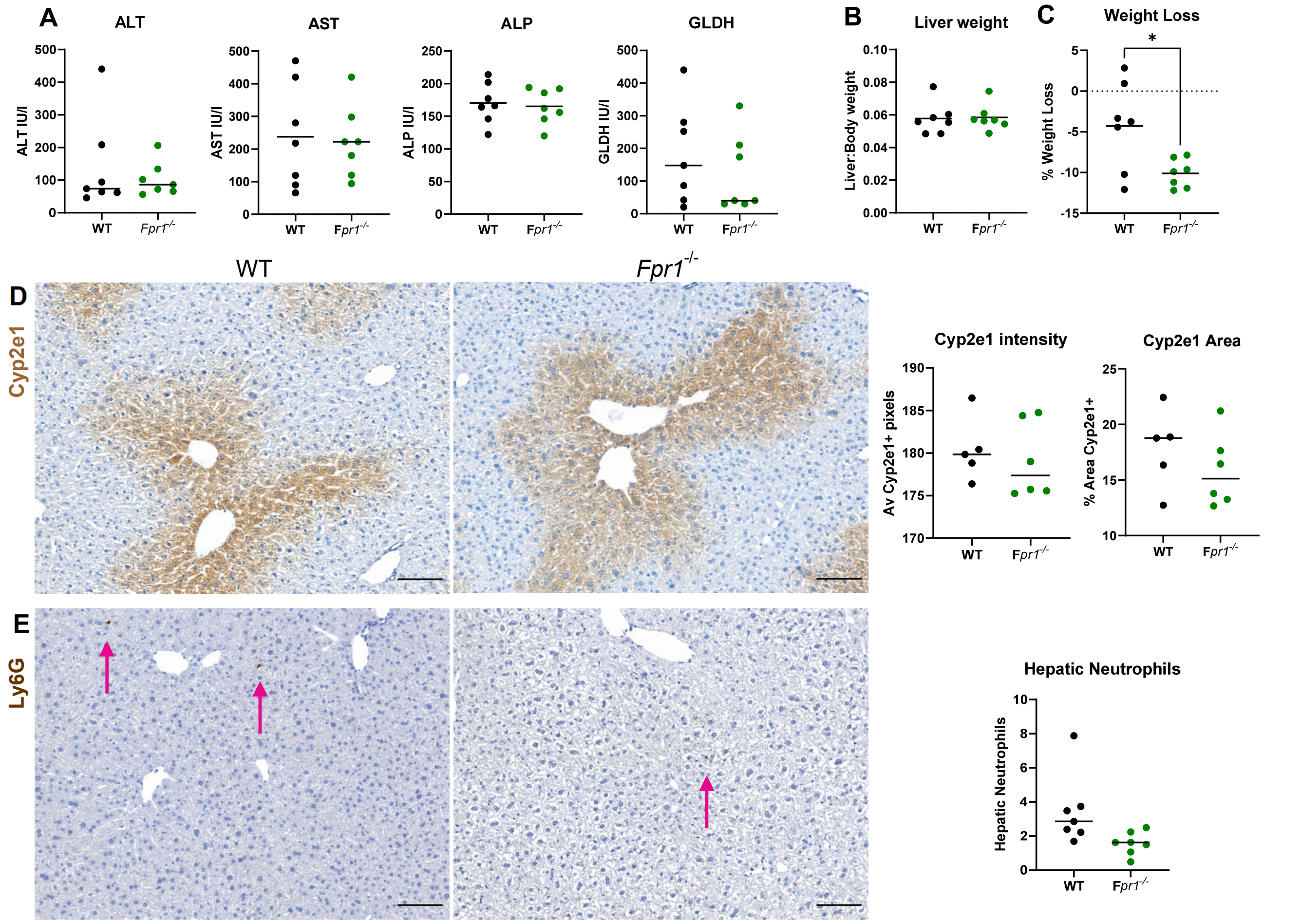

Fig. S8. Healthy *Fpr1*$^{-/-}$ mice hepatic parameters do not differ from WT

Results from WT and *Fpr1*$^{-/-}$ mice treated with APAP control. **(A),** Serum hepatic damage markers, ALT, AST, ALP and GLDH not different in *Fpr1*$^{-/-}$ mice. **(B),** Liver to body weight ratio not different to WT. **(C),** % weight loss was reduced in *Fpr1*$^{-/-}$ mice (t-test, WC, p=0.0291). **(D),** Representative Cyp2e1 labelled hepatic sections and quantification not in *Fpr1*$^{-/-}$ micl**(E),** Representative Ly6G labelled hepatic sections and neutrophil quantification showed no significant difference in *Fpr1*$^{-/-}$ mice. Each data point represents an individual mouse (n≥5). WC Welch's correction. IHC scale bars 100 µm.

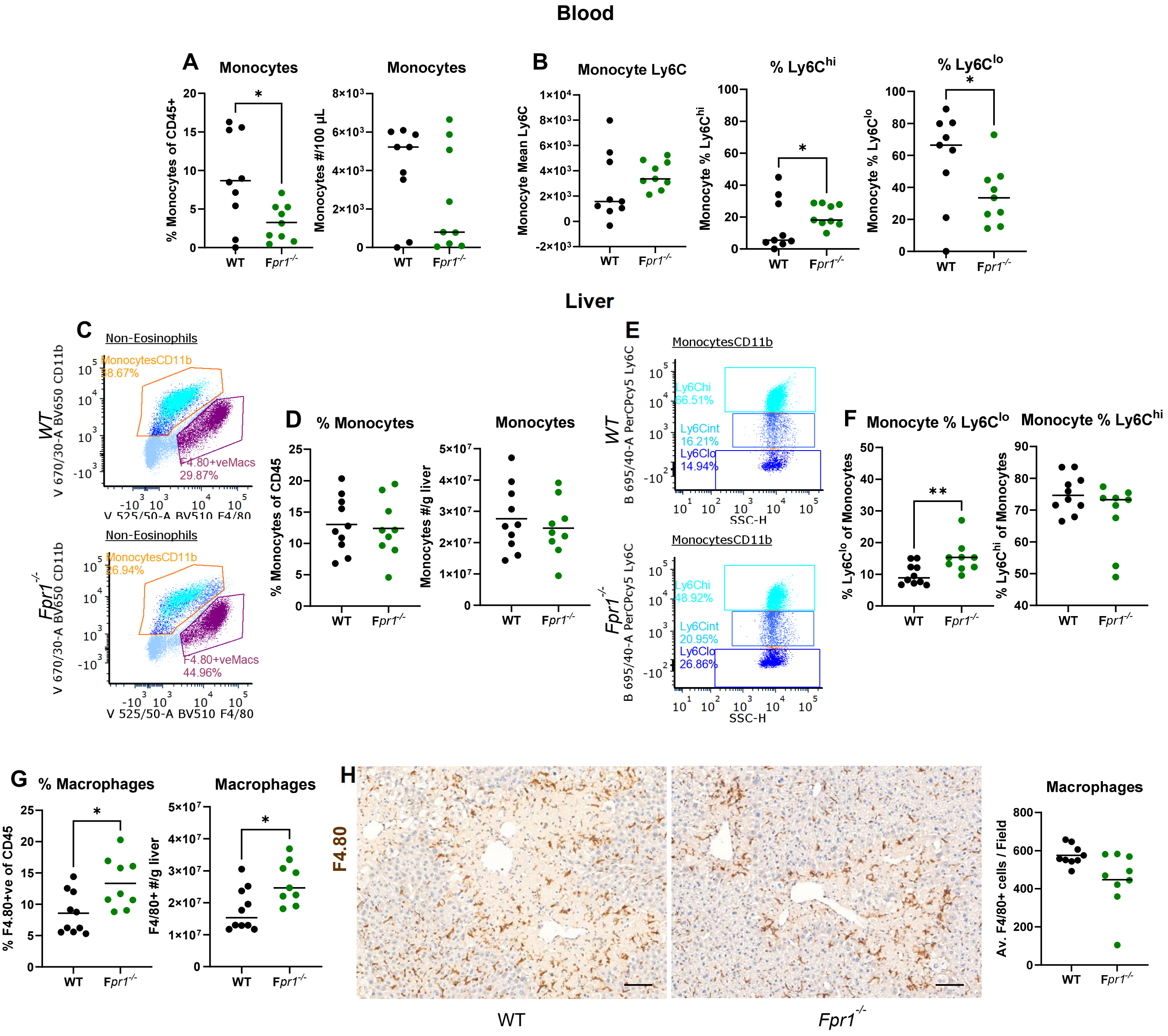

Fig. S9. Preventing FPR1-mediated neutrophil activation reduces monocyte activation

(A), Circulating monocyte % (t-test, WC, p=0.0312), reduced 16 h post APAP in *Fpr1*$^{-/-}$ mice. **(B),** Circulating monocyte higher % Ly6C^{hi} (KS, p=0.0336) and lower % Ly6C^{lo} (KS, p=0.0336) without FPR1 neutrophil activation. **(C),** Representative NPC flow plots of monocyte and macrophage populations. **(D),** Hepatic monocytes unaltered in *Fpr1*$^{-/-}$ Ie. **(E),** Representative plots of hepatic monocyte Ly6C. **(F),** Hepatic monocytes were less inflammatory with increased % Ly6C^{lo} (MW, p=0.0057). **(G),** *Fpr1*$^{-/-}$ mouse hepatic

F4/80+ macrophages % (t-test, p=0.012) and number (t-test, p=0.0132). (**H),** Representative F4/80-stained hepatic sections at 24 h post APAP show macrophage numbers stabilization. (n≥9). Representative flow plots are labelled with the parent gate and show cells/gates of interest. IHC scale bars 100 µm.

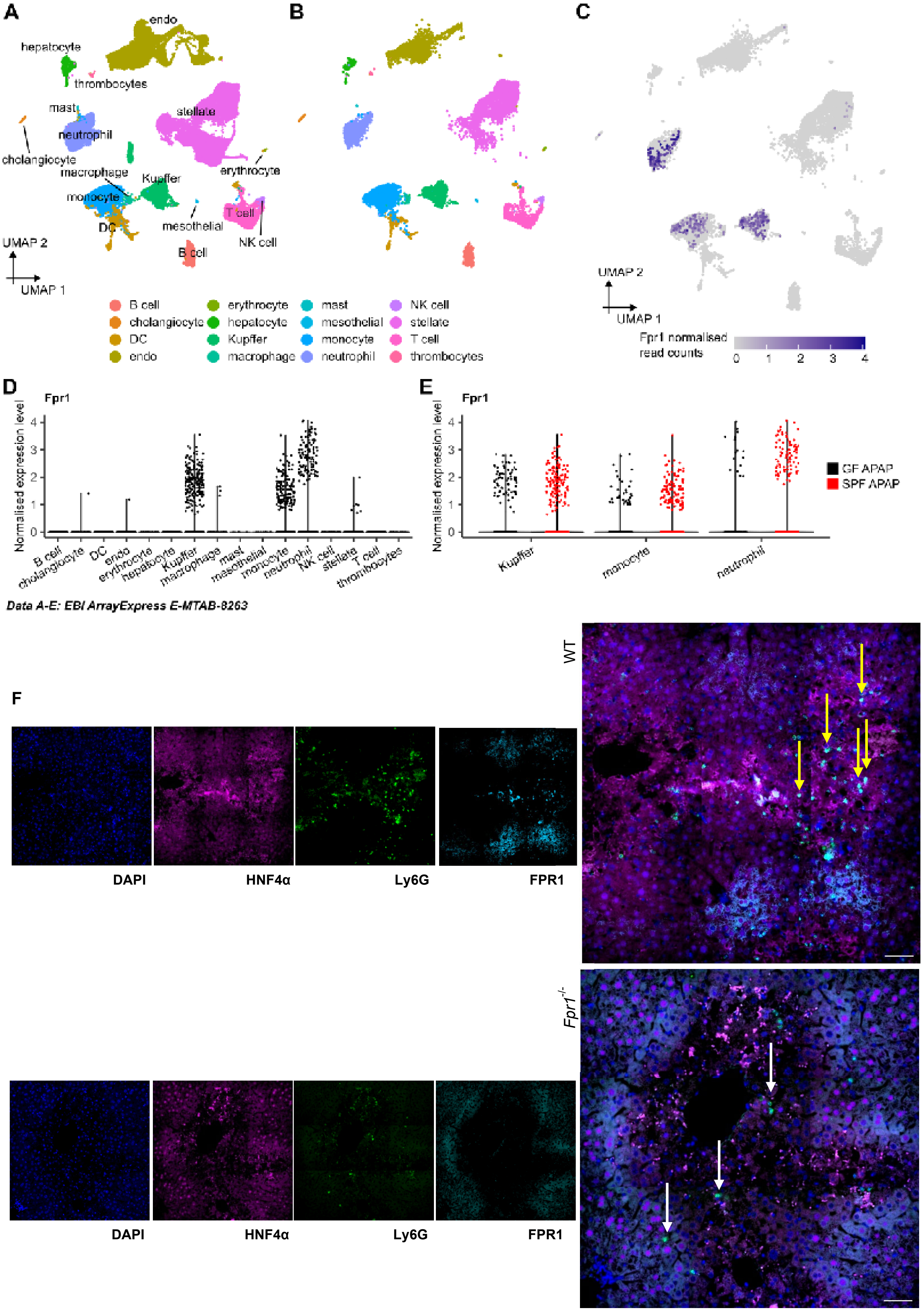
A
B
C
endo
hepatocyte
thrombocytes
mast
neutrophil
stellate
cholangiocyte
macrophage
Kupffer
erythrocyte
monocyte
T cell
DC
mesothelial
NK cell
B cell
UMAP 2
UMAP 1
B cell
cholangiocyte
DC
endo
erythrocyte
hepatocyte
Kupffer
macrophage
mast
mesothelial
monocyte
neutrophil
NK cell
stellate
T cell
thrombocytes
Fpr1 normalised read counts
0 1 2 3 4
D
E
Fpr1
Normalised expression level
GF APAP
SPF APAP
Kupffer
monocyte
neutrophil
Data A-E: EBI ArrayExpress E-MTAB-8263
F
WT
DAPI
HNF4α
Ly6G
FPR1
Fpr1-/-

Fig. S10. FPR1 is highly expressed on hepatic neutrophils following AALI

(A) UMAP of cell types in mouse liver scRNA-seq data (EBI ArrayExpress E-MTAB-8263). **(B)** UMAP of cells in APAP-treated specific pathogen-free (SPF) and germ free (GF) mice. **(C)** Fpr1 expression in SPF-APAP and GF-APAP conditions. **(D)** Violin plot of Fpr1 expression across cellypes. **(E)** Violin plot of Fpr1 expression in innate immune cells, split by experimental condition. **(F)** Representative HNF4α, Ly6G and FPR1 labelled hepatic sections, showing WT Ly6G+FPR1+ cells (yellow arrows) and Ly6G+FPR1- cells in *Fpr1*$^{-/-}$ mice (white arrows). Scale bars 50 μm

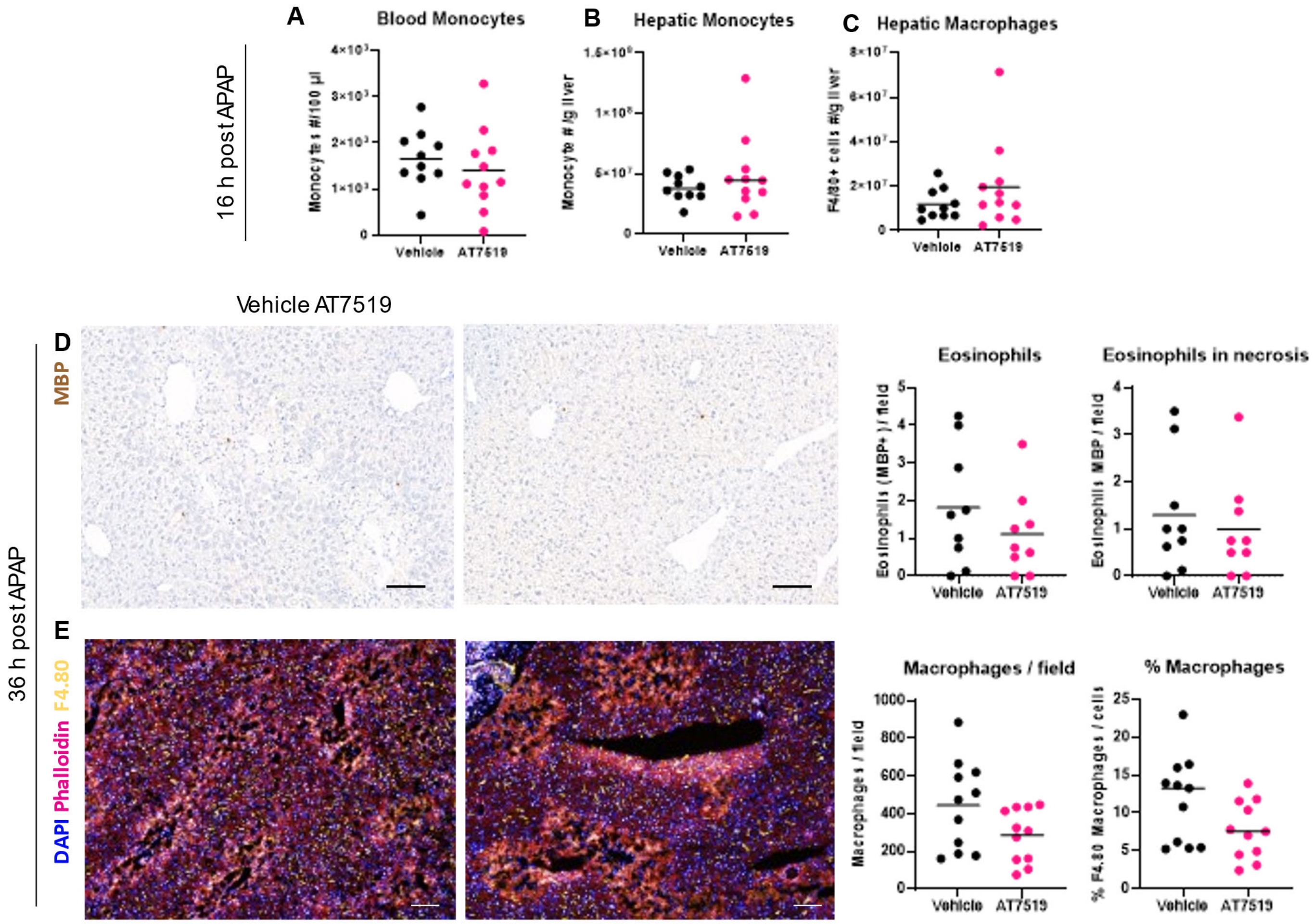

Fig. S11. AT7519-mediated neutrophil depletion is neutrophil specific

(A-C), Blood and Hepatic NPC flow cytometry 16 h after APAP, showed early 30 mg/kg AT7519 did not reduce **A,** blood monocytes, **B,** hepatic monocytes and **C,** hepatic macrophages. **(D-E),** Hepatic eosinophils and macrophages 36 h after 350 mg/kg APAP i.p. after 30 mg/kg AT7519 or vehicle at 16 h. **D,** Representative hepatic sections labelled with eosinophil major basic protein (MBP) (DAB) and quantification showed no reduction of all hepatic eosinophils or those within necrosis. **E,** Representative phalloidin and F4/80 labelled hepatic sections, demonstrated no reduction of F4/80+ macrophages / field or % positive cells. (n≥9). IHC scale bars 100 μm. IF scale bars 50 μm.

Supplementary methods

Single-cell RNA-seq analysis

We downloaded publicly-available pre-processed scRNA-seq data of mouse liver cells (n=56,527 cells) from EBI ArrayExpress (E-MTAB-8263) (Kolodziejczyk et al., 2020). Data were imported into R and subject to standard normalization and data scaling using the Seurat Bioconductor package (v4.4.0)(Hao et al., 2021). Dimensionality reduction was computed with PCA and visualised using UMAP (with the first 50 principal components, as in the original study). Data were subset to cells from paracetamol (APAP)-treated, specific pathogen-free (SPF) and germ free (GF) mice (total n=15,061 cells) for downstream analysis. Gene expression was visualised using Seurat functions.

Supplementary tables

Table S1. Flow cytometry panel for the hepatic innate immune cells

Marker	Clone	Fluorophore	Laser/Channel	Manufact	Man. Conc. mg/ml	Dilution
CD45	30-F11	AF700	R730/45	Biolegend	0.5	1:100
CD11b	M1/70	BV650	V675/30	eBio	0.2	1:100
Ly6G	1A8	PB	V445/45	BD Pharm	0.5	1:100
CD62L	MEL-14	FITC	B530/30	Biolegend	0.5	1:200
Siglec F	E50-2440	PE	YG582/15	Biolegend	0.2	1:400
F4/80	BM8	APC	R675/30	Biolegend	0.2	1:100
Ly6C	HK1.4	PerCP/Cy5.5	B675/30	Biolegend	0.2	1:100
CD3	145-2C11	PE/Cy7	YG780/60	Biolegend	0.2	1:200
CD19	6D5			Biolegend		
NK1.1	PK136			Biolegend		
L/D		E780	R780/60	eBio	-	1:1000

Table S2. Flow cytometry panel for hepatic innate immunity and neutrophil activation

<table>
<tr><th>Marker</th><th>Clone</th><th>Fluorophore</th><th>Laser/Channel</th><th>Manufact</th><th>Man. Conc. mg/ml</th><th>Dilution</th></tr>
<tr><td>CD45</td><td>30-F11</td><td>AF700</td><td>R730/45</td><td>Biolegend</td><td>0.5</td><td>1:100</td></tr>
<tr><td>CD11b</td><td>M1/70</td><td>BV650</td><td>V675/30</td><td>eBio</td><td>0.2</td><td>1:100</td></tr>
<tr><td>Ly6G</td><td>1A8</td><td>PB</td><td>V445/45</td><td>BD Pharm</td><td>0.5</td><td>1:100</td></tr>
<tr><td>MPO</td><td>2D4</td><td>FITC</td><td>B530/30</td><td>Abcam ab90812</td><td>0.1</td><td>1:100</td></tr>
<tr><td>CD62L</td><td>MEL-14</td><td>APC</td><td>R675/30</td><td>Biolegend 104412</td><td>0.2</td><td>1:100</td></tr>
<tr><td>Siglec F</td><td>E50-2440</td><td>PE</td><td>YG582/15</td><td>BD Pharm</td><td>0.2</td><td>1:400</td></tr>
<tr><td>F4/80</td><td>BM8</td><td>BV510</td><td>V525/50</td><td>Biolegend</td><td>0.2</td><td>1:100</td></tr>
<tr><td>Ly6C</td><td>HK1.4</td><td>PerCP/Cy 5.5</td><td>B675/30</td><td>Biolegend</td><td>0.2</td><td>1:100</td></tr>
<tr><td>CD3</td><td>145-2C11</td><td rowspan="3">PE/Cy7</td><td rowspan="3">Lineage Dump channel YG780/60</td><td>Biolegend</td><td rowspan="3">0.2</td><td>1:200</td></tr>
<tr><td>CD19</td><td>6D5</td><td>Biolegend</td><td>1:200</td></tr>
<tr><td>NK1.1</td><td>PK136</td><td>Biolegend</td><td>1:200</td></tr>
<tr><td>L/D</td><td></td><td>E780</td><td>R780/60</td><td>eBio</td><td></td><td>1:1000</td></tr>
</table>

Table S3. Flow cytometry panel for blood innate immunity

Marker	Clone	Fluorophore	Laser/Channel	Manufact	Man. Conc. mg/ml	Dilution
CD45	30-F11	AF700	R730/45	Biolegend	0.5	1:100
CD11b	M1/70	BV650	V675/30	eBio	0.2	1:100
Ly6G	1A8	PB	V445/45	BD Pharm	0.5	1:100
CD62L	MEL-14	FITC	B530/30	Biolegend	0.5	1:200
Siglec F	E50-2440	PE	YG582/15	Biolegend	0.2	1:400
F4/80	BM8	APC	R675/30	Biolegend	0.2	1:100
Ly6C	HK1.4	PerCP/Cy5.5	B675/30	Biolegend	0.2	1:100
CD3	145-2C11	PE/Cy7	YG780/60	Biolegend	0.2	1:200
CD19	6D5			Biolegend		
NK1.1	PK136			Biolegend		
L/D		E780	R780/60	eBio		1:1000

Table S4. Flow cytometry panel for blood innate immunity and neutrophil activation

Marker	Clone	Fluorophore	Laser/Channel	Manufact	Man. Conc. mg/ml	Dilution
CD45	30-F11	AF700	R730/45	Biolegend	0.5	1:100
CD11b	M1/70	BV650	V675/30	eBio	0.2	1:100
Ly6G	1A8	PB	V445/45	BD Pharm	0.5	1:100
MPO	2D4	FITC	B530/30	Abcam ab90812	0.1	1:100
CD62L	MEL-14	APC	R675/30	Biolegend 104412	0.2	1:100
Siglec F	E50-2440	PE	YG582/15	BD Pharm	0.2	1:400
CD115	AF598	BV510	R675/30	BD Pharm	0.2	1:100
Ly6C	HK1.4	PerCP/Cy5.5	B675/30	Biolegend	0.2	1:100
CD3	145-2C11	PE/Cy7	Lineage Dump channel YG780/60	Biolegend	0.2	1:200
CD19	6D5			Biolegend		1:200
NK1.1	PK136			Biolegend		1:200
L/D		E780	R780/60	eBio		1:1000

Table S5. Primary antibodies used for immunohistochemistry and immunofluorescence

Antibody	Clone	Dilution from Stock	Antigen retrieval	Manufacturer	Cat. Number	Host
Active Caspase 3	C92-605	1:200	TE	BD Pharmingen	BD559565	Rb
CDK9	EPR31197	1:200	TE	Abcam	ab76320	Rb
Cyp2e1	na	1:500	NaCi	Atlas	HPA009128	Rb
F4/80	A3-1	1:100	PK	Abcam	ab6640	Rt
FPR1	Polyclonal	1:150	NaCi	ThermoFisher Scientific	PA5140980	Rb
HNF4α	H1415	1:200	TE	Perseus Proteomics	PP-H1415-00	Ms
Ly6G	1A8	1:1000 IHC 1:500 IF	TE or NaCi	Biolegend	127602	Rt
MBP	MT2-14.7.3	1:1000	Digest-All™ 3 pepsin	Mayo Clinic, Arizona	Mayo Clinic, Arizona	Rt
MCM-2	Polyclonal	1:200	TE	Cell Signalling	4007S	Rb
MPO	Polyclonal	1:50	TE	Abcam	Ab9535	Rb
Phalloidin Reagent	na iFluor 647	1:1000	TE of NaCi	Abcam	Ab176759	na

Abbreviation List: Gt; Goat, Hm; Human, Ms; Mouse Rb; Rabbit, Rt; Rat, TE; TrisEDTA (ph8), PK; Proteinase K, HNF4α

Table S6. Secondary antibodies used for immunohistochemistry and immunofluorescence

Conjugate	Target Species	Fluorophore	Dilution from Stock	Manufacturer	Cat. Number	Host
Fluorescent	Rt	488	1:200	Invitrogen	A21208	Dk
Fluorescent	Ms	488	1:200	Invitrogen	A21202	Dk
Fluorescent	Rt	555	1:200	Invitrogen	A48270	Dk
Fluorescent	Rb	555	1:200	Invitrogen	A31572	Dk
Fluorescent	Ms	555	1:200	Invitrogen	A32773	Dk
Flourescent	Gt	555	1:200	Invitrogen	A32816	Dk
Fluorescent	Rb	647	1:200	Invitrogen	A32795	Dk
Fluorescent	Rt	647	1:200	Invitrogen	A78947	Dk
Biotinylated	Rb	-	1:200	Vector	BA-1000	Gt
Biotinylated	Rt	-	1:200	Vector	BA-9400	Gt
Biotinylated	Ms	-	1:200	Vector		Gt

Abbreviation List: Dk; Donkey,Gt; Goat, Ms; Mouse Rb; Rabbit, Rt; Rat

Supplementary references

Hao, Y., Hao, S., Andersen-Nissen, E., et al. (2021). Integrated analysis of multimodal single-cell data. *Cell*, *184*(13), 3573-3587.e29. https://doi.org/10.1016/j.cell.2021.04.048

Kolodziejczyk, A. A., Federici, S., Zmora, N., et al (2020). Acute liver failure is regulated by MYC- and microbiome-dependent programs. *Nature Medicine*, *26*(12), 1899–1911. https://doi.org/10.1038/s41591-020-1102-2
