# Supplementary material for: Temporal dichotomy of neutrophil function in acute liver injury and repair
Source: JHEP Rep. 2025 Apr 11;7(7):101417. doi: 10.1016/j.jhepr.2025.101417 (PMC12213964; doi:10.1016/j.jhepr.2025.101417)
Supplement: [Multimedia component 2] [file mmc2.docx]

**Journal of Hepatology**

**CTAT methods**

Tables for a “Complete, Transparent, Accurate and Timely account” (CTAT) are now mandatory for all revised submissions. The aim is to enhance the reproducibility of methods.

- Only include the parts relevant to your study
- Refer to the CTAT in the main text as ‘Supplementary CTAT Table’
- Do not add subheadings
- Add as many rows as needed to include all information
- Only include one item per row

**If the CTAT form is not relevant to your study, please outline the reasons why:**

|  |
| --- |

- 1. **Antibodies**

| **Name** | **Citation** | **Supplier** | **Cat no.** | **Clone no.** |
| --- | --- | --- | --- | --- |
| Active Caspase 3 |  | BD Pharmingen | BD559565 | C92-605 |
| CDK9 |  | Abcam | ab76320 | EPR31197 |
| Cyp2e1 |  | Atlas | HPA009128 | na |
| F4/80 |  | Abcam | ab6640 | A3-1 |
| FPR1 |  | ThermoFisher Scientific | PA5140980 | Polyclonal |
| HNF4α |  | Perseus Proteomics | PP-H1415-00 | H1415 |
| Ly6G |  | Biolegend | 127602 | 1A8 |
| MBP |  | Mayo Clinic, Arizona | Mayo Clinic, Arizona | MT2-14.7.3 |
| MCM-2 |  | Cell Signalling | 4007S | Polyclonal |
| MPO |  | Abcam | Ab9535 | Polyclonal |
| Phalloidin Reagent |  | Abcam | Ab176759 | na  iFluor 647 |

- 1. **Cell lines**

| **Name** | **Citation** | **Supplier** | **Cat no.** | **Passage no.** | **Authentication test method** |
| --- | --- | --- | --- | --- | --- |
| **na** |  |  |  |  |  |

- 1. **Organisms**

| **Name** | **Citation** | **Supplier** | **Strain** | **Sex** | **Age** | **Overall n number** |
| --- | --- | --- | --- | --- | --- | --- |
| C57BL6J WT |  | Charles River | WT | M | 8-14wks | 145 |
| *Fpr1*^-/-^ C57BL6N and colony WT | (Gao et al., 1999) | Rossi lab Colony at the University of Edinburgh | Fpr1-/- C57BL6N and colony WT | M | 8-14wks | 120 |
| *hMcl-1* transgenic mice and colony WT | *(Zhou et al., 1998)* | Rossi lab Colony at the University of Edinburgh | *hMcl-1* transgenic mice and colony WT | M | 8-14wks | 30 |

- 1. **Sequence based reagents**

| **Name** | **Sequence** | **Supplier** |
| --- | --- | --- |
| **na** |  |  |

- 1. **Biological samples**

| **Description** | **Source** | **Identifier** |
| --- | --- | --- |
| **na** |  |  |

- 1. **Deposited data**

| **Name of repository** | **Identifier** | **Link** |
| --- | --- | --- |
| Analysis of AT7519 and acetaminophen in a mouse model of acute inflammation by LC-MS/MS | Edinburgh DataShare repository: mass spectrometry of mouse serum AT7519 and APAP | https://doi.org/10.7488/ds/7686 |
|  |  |  |
|  |  |  |

- 1. **Software**

| **Software name** | **Manufacturer** | **Version** |
| --- | --- | --- |
| FCSExpress software | DeNovo | 7 |
| Prism | Graph Pad | 9.4 |
| MESO QuickPlex SQ 120 | MSD | na |
| QuickPlex SQ 120 analyzer | MSD | na |
|  |  |  |
| Analyst® software | SCIEX | 1.7.1 |
| nSolver Analysis Software | nanoString | 4.0 module 2.0.134, |
| ROSALIND® | RoalindBio (https://rosalind.bio/) (San Diego, CA | Na |
| Retiga 200R camera and Image Pro premier software | Q-imaging | na |
| inForm | Perkin Elmer | 2.4 |
| Fiji ImageJ (ImageJ Software) | National Institute of Health | Version 1.54k |
| Columbus™ software | Perkin Elmer | na |

- 1. **Other (e.g. drugs, proteins, vectors etc.)**

| **AT7519** |  | Astex Pharmaceuticals |
| --- | --- | --- |
| **APAP** |  | Sigma Aldrich |
| **Sterile Saline** |  | PanReac Applichem |
| **collagenase V** | 0.8 mg/ml; | Sigma-Aldrich |
| **collagenase D** | 0.625 mg/ml; | Roche |
| **dispase** | 1 mg/ml; | Life Technologies |
| **DNase I** | 100 mg/ml; | Roche |
| **MSD® mouse proinflammatory panel 1 V-PLEX™ plate** | containing 10 multiplexed cytokines | MSD |
| **MSD lysis buffer** | 150 mM NaCl, 20 mM Tris, 1 mM EDTA, 1 mM EGTA, 1 % TritonX-100, 2x protease inhibitor cocktail, | Sigma Aldrich |
| **Protein Block** |  | Spring Bio |
| **Bloxall** |  | Vector |
| **VECTASTAIN Elite ABC reagent** |  | Vector |
| **DAB** |  | DAKO |
| **AllPrep® DNA/RNA FFPE kit** |  | Qiagen |
| **nCounter® Mouse Myeloid Innate Immunity V2 Panel plate** |  | nanoString |

- 1. **Please provide the details of the corresponding methods author for the manuscript:**

| Jennifer Cartwright: jennifer.cartwright@ed.ac.uk |
| --- |

**2.0 Please confirm for randomised controlled trials all versions of the clinical protocol are included in the submission. These will be published online as supplementary information.**

| No clinical protocols were completed  All animal experiments were blinded until results obtained |
| --- |
