# Supplementary material for: Temporal dichotomy of neutrophil function in acute liver injury and repair
Source: JHEP Rep. 2025 Apr 11;7(7):101417. doi: 10.1016/j.jhepr.2025.101417 (PMC12213964; doi:10.1016/j.jhepr.2025.101417)
Supplement: [Multimedia component 4] [file mmc4.pdf]

Temporal dichotomy of neutrophil function in acute liver injury and repair

Jennifer A. Cartwright[1,2,3,*], Philippe M.D. Potey[1,†], Eilidh Livingstone[2,†], Lara Campana[2,†], Philip J. Starkey Lewis[2], Magdalena E.M. Oremek[1], Naomi N. Gachanja[1], Giulia Rinaldi[1,†], Rhona E. Aird[2], Tak Yung Man[2], Anuruddika J. Fernando[1], Joanna P. Simpson[4], Natalie Z.M. Homer[4], Nicole Barth[1,†], Melisande Addison[2], Candice Ashmore-Harris[2], Maria Elena Candela[2], Alastair M. Kilpatrick[2], Matthieu Vermeren[5], Calum T. Robb[1,†], David A. Dorward[1,†], Christopher D. Lucas[1], Stuart J. Forbes[2], Adriano G. Rossi[1]

JHEP Reports **2025**. vol. 7 | 1–17

Check for updates

Background & aims: Acetaminophen (APAP)-induced acute liver injury (APAP-ALI) is the leading cause of acute liver failure-induced death, with host innate immune responses driving outcomes. Neutrophils are activated and increased in APAP-ALI and reported to contribute to liver damage. However, neutrophil dysfunction in patients with acute liver failure is associated with non-survival, and recent reports highlight their importance in hepatic repair. Neutrophil-targeted therapies for APAP-ALI are hampered by this controversy and a lack of time-dependent investigation.

Methods: Hepatic neutrophils were depleted at different times in a wild-type mouse model of APAP-ALI. *Fpr1*$^{-/-}$ mice, with reduced neutrophil activation, were also used. The impact of neutrophil depletion was interrogated during hepatic injury and repair after APAP-ALI, using serum biochemistry, liver and blood flow cytometry, liver histopathology, immunohistochemistry, ELISA, and NanoString analysis.

Results: Neutrophils contributed both to hepatic damage and repair after APAP-ALI. Early liver necrosis was reduced by neutrophil depletion (34% to 23%, p = 0.0018, n ≥10) and by reducing neutrophil functions (39% to 29%, p = 0.0279, n ≥11). By contrast, late neutrophil depletion resulted in markedly reduced liver repair (persistent necrosis 17% to 30%, p = 0.016, and higher serum alanine aminotransferase [1,221 to 3,725 IU/l, p = 0.0007, n ≥10]) and hepatocyte proliferation (decreased mini-chromosomal maintenance 2+ hepatocytes, 3% to 1%, p = 0.025, n = 10). Late neutrophil depletion reduced proliferation, growth factors, and angiogenesis transcripts (Mik6 fold change [FC] -6.322, p = 0.002; Socs2 FC -2.91, p = 0.01; vascular endothelial growth factor A FC -1.48, p = 0.01; n = 3). Similar transcript changes were identified when preventing formylated peptide receptor 1-mediated neutrophil activation, along with reduced extracellular matrix remodeling (Col12a1, FC -1.99, p = 0.0001; n ≥5). Finally, depleting neutrophils resulted in a hepatic proinflammatory monocyte/macrophage phenotype during repair stages, with increased proinflammatory-related transcripts and reduced reparative transcripts.

Conclusion: Recruited neutrophils contribute not only to hepatic damage early in APAP-ALI, but also to hepatic repair through a variety of pathways, including extracellular matrix remodeling, angiogenesis, hepatocyte proliferation, and promotion of an anti-inflammatory monocyte/macrophage phenotype.

© 2025 The Authors. Published by Elsevier B.V. on behalf of European Association for the Study of the Liver (EASL). This is an open access article under the CC BY license (http://creativecommons.org/licenses/by/4.0/).

Introduction

Acetaminophen (paracetamol; APAP)-induced acute liver injury (ALI), the leading cause of acute liver failure (ALF) in the Western world,[1–3] has no effective treatments for late presentations, and patients progressing to ALF have a worse prognosis compared with those with other causes of ALF.[4] Patients with APAP-ALI have pronounced increases in circulating activated neutrophils,[5,6] and hepatic necrosis is characterized by marked inflammation and early infiltration of neutrophils.[7] Patients with APAP-ALI progressing to ALF have reduced neutrophil functions[8,9] and such patients are at risk of fatal bacterial infections,[3] indicating a potential crucial role of neutrophils in recovery from APAP-ALI.

Neutrophils, the most abundant human circulating leukocyte, provide crucial host defenses against invading pathogens. They are enigmatic cells with myriad immunological processes, functional plasticity, and heterogeneity.[10,11] Numerous important neutrophil functions continue to emerge, such as during tumor metastasis, and in autoimmunity, chronic diseases,[12] and, increasingly, tissue restoration.[13] Although

* Corresponding author. Address: The Royal (Dick) School of Veterinary Studies, University of Edinburgh, Edinburgh, UK. Tel.: +44 7849 322511.
E-mail address: Jennifer.cartwright@ed.ac.uk (J.A. Cartwright).
† Current addresses: National Institute for Health and Care Research, Twickenham, UK (PMDP); Novo Nordisk Research Centre, Hepatic Target Discovery, Oxford, UK (EL); Resolution Therapeutics, Centre for Regenerative Medicine, University of Edinburgh, Edinburgh, UK (LC); Department of Physiology Development and Neuroscience, University of Cambridge, UK (GR); Institute of Genetics and Cancer, Edinburgh, UK (NB); Thermo Fisher Scientific, Paisley, UK (CTR); Department of Pathology, Royal Infirmary of Edinburgh, Edinburgh, UK (DAD).
https://doi.org/10.1016/j.jhepr.2025.101417

their uncontrolled activation can be detrimental,[14] neutrophils are also known to orchestrate inflammation resolution and subsequent tissue repair.[15,16]

In APAP-ALI, neutrophils are recruited in response to numerous signals, including tissue damage-associated molecular patterns (DAMPs), particularly mitochondrial formylated peptides,[7] which bind to formylated peptide receptor 1 (FPR1), resulting in receptor upregulation, and neutrophil activation[17,18] and migration.[19,20]

APAP-ALI is difficult to investigate in patients because of many factors; therefore, mouse model studies are frequently used as an alternative.[21] Cytopenias are a known negative risk factor for APAP-ALI[22] and significant neutrophil function abnormalities have been related to outcomes in patients with ALF.[9,23] The role of neutrophils in mouse models of APAP-ALI remains controversial, with several reports indicating that they potentiate hepatic tissue damage,[19,21,24–26] while others contest this, reporting their importance in hepatic repair.[27–30]

Neutrophils can be modulated to resolve inflammation, and neutrophil products, such as membrane-derived nanovesicles and extracellular vesicles, have been considered as treatments for inflammatory conditions.[31,32] However, the unresolved functions of neutrophils in APAP-ALI and their role in ALF and prevention of sepsis, impede discovery of potential neutrophil-targeted therapeutic strategies.

All previous studies of neutrophil function have assessed this in either injury or repair and none have evaluated their functions over time. To elucidate the neutrophil role over the course of APAP-ALI, we pharmacologically depleted neutrophils at different times in a wild-type (WT) mouse model, as well as using *Fpr1*$^{-/-}$ mice, with reduced neutrophil activation. We assessed blood and tissue neutrophil activity alongside hepatic damage and tissue regeneration markers during APAP-ALI. Through these techniques, we show that circulating and hepatic neutrophils are activated rapidly after APAP and remain so during repair. We reveal a time-dependent dual role of neutrophils in APAP-ALI, with early depletion resulting in decreased hepatic necrosis during injury, and late depletion resulting in reduced repair. These findings are reinforced in *Fpr1*$^{-/-}$ mice, demonstrating that neutrophils contribute to both hepatic tissue injury and repair through FPR1-mediated functions. Findings in both models highlight alterations in hepatic angiogenesis and extracellular matrix (ECM) remodeling during repair following neutrophil modulation, alongside an altered monocyte/macrophage phenotype. Our findings address the longstanding and continued controversy surrounding the role of neutrophils in APAP-ALI, and highlight the importance of considering neutrophil plasticity and time-dependent tissue context for therapeutic targeting.

Materials and methods

All methods are additionally detailed in[33] and the supplementary CTAT Table.

Mice

C57BL/6 male mice were purchased from Charles River (Edinburgh, UK). *Fpr1*$^{-/-}$ mice[34] and human myeloid cell leukemia factor 1 (*hMcl1*) transgenic mice[35] were propagated at the University of Edinburgh, both with WT colony controls. Mice housed in groups in ventilated cage systems were acclimatized for 1 week before experiments and synchronized to a 10–14 h dark/light cycle with access to food and water *ad libitum*. All experiments had local ethical approval, were conducted under UK Home Office legislation, and conformed to Animal Research: Reporting of In Vivo Experiments (ARRIVE) guidelines. Genotyping was carried out using PCR by TransnetYX.

APAP model

Male mice (8–14-weeks old) were fasted for 12 h before receiving a 350 mg/kg i.p. injection of either APAP in sterile saline or sterile saline (control). Standard chow and mash were returned to mice 20 min after injection and all mice were maintained in warming cabinets (28 °C). Vehicle (sterile saline) or 30 mg/kg AT7519 (Astex Pharmaceuticals, Cambrdge, UK), a selective CDKI[36] in sterile saline, were given i.p. at 4 h or 16 h post APAP. At predetermined times post APAP, mice were humanely culled according to local ethical guidelines and whole blood was collected. During APAP experiments, mice were monitored for six behavioral phenotypes of clinical severity (scoring 0–3); hunching, piloerection, neurological symptoms, responsiveness to touch, skin pallor, and breathing effort.

Nonparenchymal cell labeling and flow cytometry analysis

Isolation of the hepatic nonparenchymal cell (NPC) fraction was achieved as detailed elsewhere[37] with minor alterations. Briefly, mouse livers were perfused *in situ* with 10 ml 0.9% NaCl through the inferior vena cava. Livers were harvested and weighed, and 0.4 g of tissue was homogenized and digested in RPMI 1640 containing collagenase V (0.8 mg/ml; Sigma-Aldrich, Glasgow, UK), collagenase D (0.625 mg/ml; Roche, London, UK), dispase (1 mg/ml; Life Technologies, Cambridge, UK), collagenase D (1.6 mg/ml; Roche), and DNase I (100 mg/ml; Roche) for 25 min at 37 °C in a shaking incubator. Cold RPMI 1640 with 10% fetal calf serum (FCS) was added to 70 μm strained digests to deactivate enzymes. Immune cell-containing fractions were harvested by two wash centrifugations (300 g, 4 °C, 5 min), followed by red blood cell (RBC) lysis using 3 ml RBC lysis buffer (Sigma-Aldrich) for 3 min at room temperature (RT). Cells were counted, and stained with a Fixable Viability Dye eFlour™ 780 (Invitrogen 65-0865) for 30 min. Non-specific binding was blocked with 10% mouse serum (4 °C, 5 min), followed by incubation with primary antibodies (Table S1–S4), (40 min, 4 °C). Samples were then processed on a BD LSR Fortessa 4 laser and analyzed using FCSExpress software (DeNovo, Pasadena, USA). Absolute cell numbers were quantified per gram of liver through a proportion of counted liver digest NPCs, factoring for liver weight.

Plasma chemistry evaluation

Serum analysis was performed by the Specialist Assay Service at the MRC Centre for Reproductive Health (University of Edinburgh, Edinburgh, UK), using a commercial kit (Alpha Laboratories, Hampshire, UK) for alanine aminotransferase (ALT) and albumin, on the Cobas Fara centrifugal analyzer (Roche Diagnostics Ltd, UK). Aspartate aminotransferase (AST), alkaline phosphatase (ALP), and glutamate dehydrogenase (GLDH) were quantified using commercial kits (Alpha Laboratories) on the Mira analyzer (Roche Diagnostics, UK).

Multiplex Meso Scale Discovery ELISA assessments

Serum and liver cytokine concentrations were quantified using a Meso Scale Discovery (MSD) multiplex ELISA system. Liver was lysed with MSD lysis buffer (150 mM NaCl, 20 mM Tris, 1 mM EDTA, 1 mM ethylene glycol-bis(β-aminoethyl ether)-N,N,N′,N′-tetraacetic acid) (EGTA), 1% TritonX-100, 2x protease inhibitor cocktail, [Sigma Aldrich]) and homogenized in a Precelly's tissue homogenizing tube (P000918-LYSK0-A) (two cycles of 2,000 g, 30 s). The liver lysis supernatant and serum protein were quantified with a Pierce™ BCA protein microplate assay and measured using a MESO QuickPlex SQ 120 plate reader (562 nm absorbance). Samples were assessed on MSD® mouse proinflammatory panel 1 V-PLEX™ plates containing ten multiplexed cytokines (IFNγ, IL10, IL12p70, IL1β, IL2, IL4, IL5, IL6, CXCL1, and TNFα) using a QuickPlex SQ 120 analyzer (MSD).

Mouse serum liquid chromatography-mass spectrometry

AT7519 and a deuterated standard $^{2H}{}_{8}$-AT16043M (d8-AT7519) were gifted by Astex Pharmaceuticals. APAP was purchased from Apollo (Denton, UK) and $^{2H}{}_{4}$-APAP (d4-APAP) from Cerilliant® (Merck, Watford, UK). Following a previously described method,[38] samples were injected into a Waters Acquity UPLC BEH C18 column (2.1x100 mm, 1.7 µm; Waters, Wilmslow, UK) maintained at 45 °C. Mass analysis was performed on a QTrap 5500 triple quadrupole mass spectrometer (AB Sciex, Warrington, UK) in positive ion mode. Liquid chromatography (LC)-mass spectrometry (MS)/MS data were collected using Analyst® 1.7.1 software.

RNA isolation and NanoString

An AllPrep® DNA/RNA FFPE kit (Qiagen, Manchester, UK) was used for purification of RNA from formalin-fixed paraffin-embedded (FFPE) tissues for use with the nCounter® Mouse Myeloid Innate Immunity V2 Panel plate (NanoString, Washington, USA). Briefly, RNA was extracted from 10 µ-thick sections using RNeasy MinElute spin columns (Qiagen) according to the manufacturer's protocol. RNA was quantified using the Nanodrop Spectrophotometer (Thermo Fisher Scientific) and RNA quality was assessed using a LabChip® GX Touch/GXII Touch nucleic acid analyzer (PerkinElmer, Springfield, USA) (QMRI Biomolecular Core, University of Edinburgh). Samples were processed by the Host and Tumour Profiling Unit (University of Edinburgh, UK) following the manufacturer's guidelines, using the nCounter® Prep station and nCounter® Analysis system (NanoString).

NanoString data analysis

nSolver 4.0 Analysis Software (module 2.0.134, NanoString) was utilized for *Fpr1*$^{-/-}$ *vs.* WT analysis, using the advanced analysis 2.0 plug-in. Background thresholding was increased to 50 probe counts and comparisons were set between genotypes. After removal of low raw counts and QC flags, a strict requirement was set for a $\log_2$ fold change with the threshold of >1.25 or <-1.25 and p <0.05.

ROSALIND® (https://rosalind.bio/; San Diego, CA, USA), was used to analyze AT7519 *vs.* vehicle data. nCounter® reporter code count data were imported and files were annotated. Datasets were analyzed with ROSALIND HyperScale architecture. GeNorm selected normalization housekeeping probes were used from the NormqPCR R library.[39] Benjamini–Hochberg p value adjustments were performed and Partitioning Around Medoids was used for the final clustering of genes. This used the fpc R library[40] considering the direction and type of all signals on a pathway, and the position, role and type of every gene. Analysis comparing groups was completed with a threshold fold change of ≥ 1.25 or ≤ -1.25 and a significance set at p <0.05.

Immunohistochemistry

Liver tissue was harvested and fixed in 4% paraformaldehyde (PFA) followed by 70% ethanol before paraffin embedding. Heat-induced epitope retrieval of deparaffinized tissue sections was in 0.01 M sodium citrate buffer (pH 6), or TrisEDTA (pH 8) for 15 min, or Proteinase K for 10 min at 37 °C depending on the antibody. For major basic protein (MBP), Digest-All™ 3 pepsin was used for 10 min and the wash buffer was 0.05 M Tris-HCl, 0.15 M NaCl, 0.05% Tween 20, pH 7.6. For mini-chromosomal maintenance (MCM)-2, sections were permeabilized in PBS 0.1% Tween 20 (PBST) for 5 min, followed by wash steps including PBST. Sections visualized with 3,3′-diaminobenzidine (DAB) were sequentially blocked at RT with Bloxall (Vector, Newark, CA, USA), Avidin, and Biotin (Invitrogen, Cambridge, UK) for 15 min each, followed by Protein Block (Spring Bio). Sections for immunofluorescence were blocked for 30 min with Protein Block. DAB and immunofluorescent sections were incubated overnight at 4 °C with primary antibodies (Table S5 and supplementary CTAT Table). For DAB stains, sections were incubated (1 h at RT) with a biotinylated secondary antibody (Table S6), followed by 30 min avidin-based peroxidase reagent R.T.U. VECTASTAIN Elite ABC reagent (Vector) and DAB (DAKO,Glostrup, Denmark) and Harris' hematoxylin counterstain. Immunofluorescence labeling was completed with secondary conjugated antibodies (Invitrogen 1:200) and DAPI (1:1,000).

Microscopy and imaging analysis

Fluorescent and bright field images were acquired on a Nikon Eclipse e600 microscope with a Retiga 200R camera (Q-imaging, Image Pro premier software), DMi8 (Leica Microsystems, Milton Keynes, UK), Zeiss Axioskop microscope, or EVOS M7000 Imaging System (Thermo Fisher Scientific). Bright field images were also acquired on a Vectra® Polaris™ multi spectral slide scanner (PerkinElmer), with fluorescent images collected on an Operetta CLS High Content Analysis System (PerkinElmer). DAB-positive cell quantification was completed with inForm 2.4 (PerkinElmer) and inForm spectral unmixing tissue segmentation was utilized to quantify necrosis. Numbers and percentage of immunofluorescent positive cells were analyzed using Columbus™ software (PerkinElmer). Hepatic cytochrome P450 2E1 (Cyp2e1) quantification was analyzed using Fiji ImageJ (ImageJ Software, National Institute of Health, Bethesda, MD, USA: http://rsb.info.nih.gov/ij/)). FPR1-labeled sections were imaged with a ZIESS observer 7 and adapted for publication with ImageJ.

Isolation of mouse bone marrow neutrophils

Mouse long bone marrow was flushed into a 50 ml conical tube with Hank's buffered salt solution (HBSS)-prep (Ca–Mg-free

HBSS, 20 mM Na-HEPES, 0.5% FCS, 1% penicillin and streptomycin) using a 25-g needle. Bone marrow was disaggregated and cells were pelleted by centrifugation (400 **g**, 5 min). The pellet was resuspended in 5 ml 0.2% NaCl for 45 s to lyse RBCs and osmolarity was restored with 5 ml of 4 °C 1.6% NaCl. Cell strained (40 μm) suspensions were centrifuge (400 **g**, 5 min) and re-suspended in 5 ml HBSS-prep, then layered on 5 ml of 62% Percoll in a 15-ml falcon tube. This was centrifuged at 1,000 **g** for 30 min (acceleration 5, 0 brake). Pelleted cells and immature granulocytes were resuspended, counted, and then centrifuged and resuspended in HBSS-prep to $10x10^6$/ml.

In vitro neutrophil activation assay

After separation, granulocytes were diluted to $20x10^6$/ml in $HBSS^{+/+}$ and 180 μl was added to a 2-ml Eppendorf/reaction tube with 20 μl of activation reagent (formylated peptide, platelet activating factor [PAF], or control PBS). This was agitated for 30 min at 37 °C. For shape change analysis, cells were fixed with 4% PFA (15 min). For assessment of activation markers, cells were transferred to fluorescence-activated cell sorting (FACS) tubes with conjugated antibodies (Ly6G, CD11b, and CD62L, all at 1:100) and incubated at RT for 30 min before washing and fixing. Cells were then analyzed with a BD LSR Fortessa 4 laser, or BD Accuri C6.

Study design

Statistical power analysis, to identify mouse sample size estimation for AT7519 treatment experiments, was performed based on *in vivo* data from a published study[41] where n = 6, which compared untreated and cyclin-dependent kinase inhibitor (CDKI)-treated mice with acute pulmonary inflammation using a significance of p <0.05. Depending on the translatable measured variable, the effect size (ES) in this study was 1.16–3.2, considered to be large to very large using Cohen's criteria.[42] With an alpha = 0.05 and power = 0.80, the projected sample size needed, with a median ES of 1.5, was n = 9 for a between-group comparison. A similar sample size estimation of n = 8 for the comparison of WT and $FPR1^{-/-}$ mice was identified using published data with a model of acute pulmonary inflammation at p <0.05.[43] Unpublished necrosis comparison data from APAP-treated mice with and without interventions (Forbes *et al.*, unpublished data, 2025), gave an ES of 1.96. Statistical power analysis for a one-way ANOVA gave a required n = 9. All tests were performed using commercially available software, G*Power.[44] Therefore, the proposed sample size was set as a minimum of nine for APAP-ALI studies for all genotypes and interventions. This was determined to be adequate for the main objective of this study, allowing for expected attrition and controlling for possible mediating and moderating factors and subgroup analysis.

Data analysis and statistics

Unless otherwise stated, statistical analysis was performed in Prism 9.4 (GraphPad software). All data are presented as individual scatter plots and show each experimental data point (*e.g.* individual mice) unless otherwise stated. Any data with a small n (<6) were treated as nonparametric. Gaussian distribution was otherwise assessed by a Shapiro–Wilk normality test. When normally distributed, the mean is expressed on all graphs, whereas medians are shown for nonparametric data. To test two groups, an unpaired 2-way t test with or without Welch's correction for different group variance, or a Mann-Whitney (MW) U test or Kolmogorov–Smirnov (KS) test for cumulative distribution was performed on parametric and nonparametric datasets, respectively. To test two or more parametric groups, a one-way ANOVA, two-way ANOVA (with Dunnett's multiple comparison test), or mixed-effects model (with Sidak's multiple comparison's test) was performed. To test two or more nonparametric groups, a Kruskal–Wallis test (with Dunn's multiple comparison test) was performed. p <0.05 was considered statistically significant.

Results

Depleting neutrophils in APAP-ALI, reduces hepatic injury and repair

To interrogate the controversial role of neutrophils during APAP-ALI over time, neutrophils were depleted during early (4 h, before peak necrosis) or late (16 h, after peak necrosis) injury in a mouse model of APAP-ALI, using a single i.p. injection of AT7519, a CDKI shown to deplete neutrophils.[45,46] Pharmacological depletion was used given the known limitations of anti-Ly6G or anti-Gr-1,[47] which was in agreement with our findings that neutrophils were ineffectively depleted by intravenous Ly6G after established APAP-ALI. Assessments were completed during both injury and repair phases after establishing these timings in the WT mouse model (Fig. 1A; Fig. S1A–D). AT7519, given either during early injury or at peak hepatic neutrophil recruitment as assessed by immunohistochemistry (Fig. S1E and F) and flow cytometry (Figs S1G,H and S2), dramatically reduced hepatic neutrophil numbers (Fig. 1B–E). Early hepatic neutrophil depletion was associated with a significant early reduction in hepatic damage, with reduced mouse clinical severity score and hepatic necrosis. When depletion was performed late, at peak neutrophil infiltration (16 h) with assessment during hepatic repair, mice showed more weight loss and higher clinical severity. Serum biomarkers of hepatic injury (ALT, AST, and GLDH) were also higher along with persistent hepatic necrosis (Fig. 1E–M). This depletion dichotomy illustrated the time-dependent role of neutrophils in APAP-ALI.

AT7519-mediated neutrophil depletion is neutrophil specific

The inflammation resolution mediated by early AT7519 neutrophil depletion shown here is commensurate with published studies of AT7519 improving return of tissue homeostasis and animal recovery.[45,46,48] However, AT7519-mediated neutrophil depletion has not been previously associated with reduced tissue repair. Therefore, further assessments investigated the neutrophil specificity of this reduced repair phenotype. AT7519 30 mg/kg had no effect on hepatic injury biomarkers in APAP control-treated (healthy) mice and caused no hepatic necrosis or increased hepatic cleaved caspase 3 (CC3) expression. There was also no reduction in hepatic proliferation (Fig. S3A–G). A lower AT7519 dose of 10 mg/kg was insufficient to deplete hepatic neutrophils in APAP-ALI (Fig. S3H) and there was also no alteration in hepatic repair

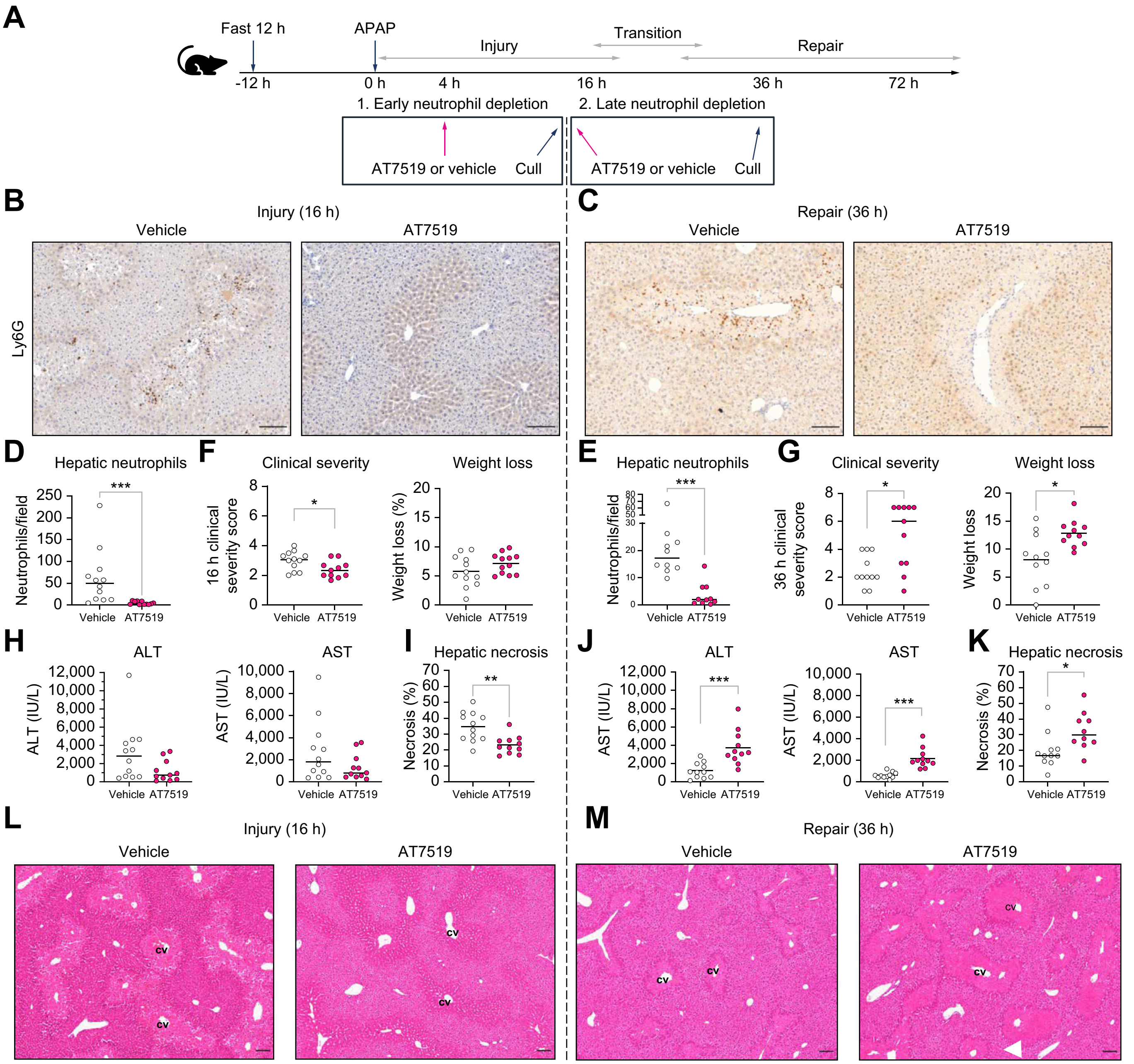

Fig. 1. AT7519-mediated depletion of neutrophils reduces hepatic injury and repair. (A) Model schematic. (B,C) Representative Ly6G-labeled hepatic sections, displaying AT7519-reduced neutrophils, analyzed with KS (D) $p = 0.0001$ and (E) $p = 0.0006$. (F) Early neutrophil depletion improved clinical severity (*t* test, $p = 0.0254$). (G) Clinical severity (MW, $p = 0.012$) and weight loss (*t* test, WC, $p = 0.014$) worsened during repair. (H) ALT and AST during injury. (J) Elevated ALT (*t* test $p = 0.0007$) and AST (*t* test, WC, $p = 0.0001$) during repair. (I) Early neutrophil depletion reduced necrosis (*t* test, $p = 0.0018$). (K) Necrosis increased during repair (MW, $p = 0.016$). (L,M) Representative H&E hepatic sections showing necrosis around CVs. N ≥10 in all cases; scale bars: 100 μm. ALT, alanine aminotransferase; AST, aspartate aminotransferase; CV, central veins; KS, Kolmogorov–Smirnov; WC, Welch's correction; MW, Mann-Whitney. In all instances: **p* <0.05, ***p* <0.005, ****p* <0.0005, *****p* <0.0001.

without neutrophil depletion (Fig. S3I–M). Hepatic Cyp2e1 expression, the main enzyme that metabolizes APAP into its toxic metabolite *N*-acetyl-p-benzoquinoneimine, was unaltered after AT7519 in healthy or APAP-ALI mice (Fig. S3N). Mass spectrometry analysis of serum APAP and AT7519 identified no correlation between AT7519 and APAP concentrations, although AT7519 was higher in animals with APAP toxicity (Fig. S4A–E), indicating reduced metabolism of this drug following APAP-ALI. There was no correlation of serum AT7519 concentration with measures of hepatic damage, hepatic necrosis, and hepatic CC3, or with hepatic proliferation (Fig. S4F–I).

Hepatic and circulating neutrophils are activated following APAP-ALI

To further investigate the dichotomous time-dependent role of neutrophils in APAP-ALI, the WT neutrophil response was

additionally assessed. Hepatic neutrophil recruitment occurred early in our mouse model and, along with activation, peaked at 16 h post APAP-ALI. Neutrophils were activated following APAP-ALI, with higher CD11b expression and increased CD62L shedding following APAP-ALI (Fig. 2A–G), with this activation continuing for longer than previously observed.[49] We identified persistently elevated hepatic numbers during repair (Fig. S1E), along with lower circulating neutrophil CD62L (Fig. 2C). Hepatic neutrophils also had lower expression of CC3 following APAP-ALI (Fig. 2H–J), suggesting reduced neutrophil apoptosis resulting from increased neutrophil activation and prolonged survival following APAP-ALI, as seen in other inflammatory conditions.[50,51]

AT7519 depletes hepatic neutrophils through CC3-induced apoptosis

APAP-ALI neutrophil survival cues were overcome by AT7519, as shown by marked blood and hepatic neutrophil reduction (Fig. S5A–D). AT7519 is a selective CDK9 inhibitor that down-regulates the neutrophil-required survival protein MCL1, which, in turn, results in CC3-mediated apoptosis.[41,52] Hepatic CDK9 expression was not affected by AT7519 during APAP-ALI (Fig. S5E), but AT7519 increased the percentage hepatic neutrophil CC3 expression (Fig. S5F and G), a result previously not shown *in vivo*.

Overexpressing MCL1 does not increase neutrophils in APAP-ALI

Overexpressing MCL1 increases tissue granulocyte numbers by increasing their survival.[41] Mice expressing *hMcl1*, which decreases neutrophil apoptosis, were used to assess the impact of increased neutrophil numbers on APAP-ALI. Interestingly, there was no difference in hepatic neutrophil number in *hMcl1* mice compared with WT during APAP-ALI (Fig. S5H and I), indicating the MCL1 neutrophil survival pathway is already saturated. Without a difference in neutrophil number, there were no alterations in the hepatic damage phenotype, including no changes in weight loss, serum markers of hepatic damage, or hepatic necrosis (Fig. S5J–L).

Preventing FPR1 ligation reduces hepatic neutrophil recruitment and activity

To further evaluate the novel time-dependent role of neutrophils, neutrophil activation was reduced using a mouse genetic knock out of FPR1 (*Fpr1*$^{-/-}$), a key driver of neutrophil activation and migration.[53,54] During APAP-ALI, DAMPs, ligands for

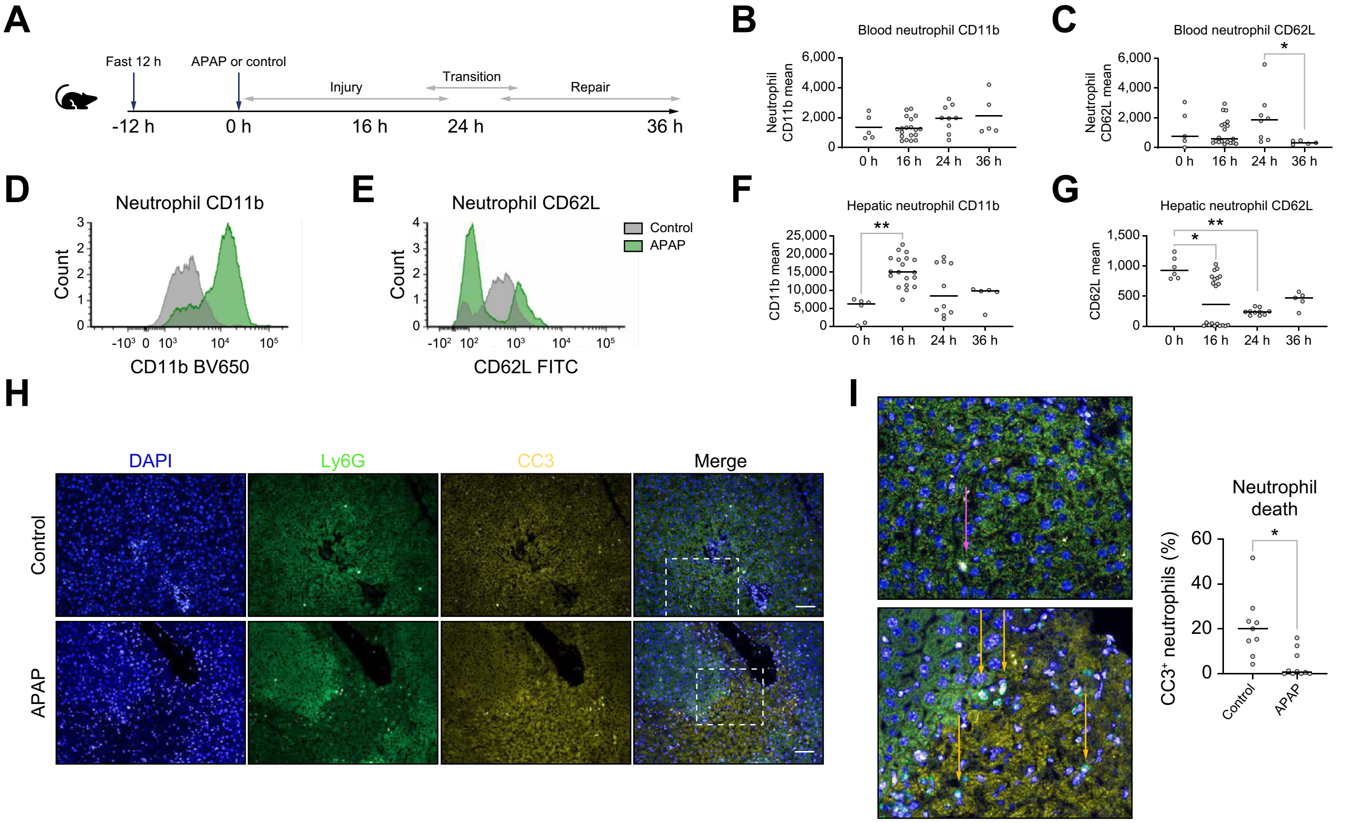

Fig. 2. Circulating and hepatic neutrophils are activated following APAP with prolonged survival. (A) Model schematic. (B) Blood neutrophil CD11b expression (KW, p = 0.1899). (C) Decreased blood neutrophil CD62L expression, following APAP (KW, p = 0.0456, 24 h *vs.* 36 h, p = 0.0344). (D,E) Representative hepatic neutrophil flow cytometry. (F) Increased neutrophil CD11b following APAP (KW, p = 0.00008; 0 *vs.* 16, p = 0.0015) and (G) decreased CD62L expression (KW, p = 0.00088; 0 h *vs.* 16 h, p = 0.0126; 0h *vs.* 24 h, p = 0.0086. (H) Representative Ly6G and CC3 hepatic sections at 36 h post APAP administration and in controls. (I) Magnifications showing CC3+ (pink arrows) and CC3- neutrophils (orange arrows). (J) Decreased percentage of CC3+ neutrophils (KS, p = 0.0193). Scale bars: 50 µm; n ≥5. APAP, acetaminophen (paracetamol); KS, Kolmogorov-Smirnov; KW, Kruskal–Wallis. In all instances: *p <0.05, **p <0.005.

FPR1, are released, resulting in neutrophil activation and hepatic recruitment.[19] *Fpr1*$^{-/-}$ mouse neutrophils were not stimulated by formylated peptides *in vitro*, showed no increase in CD11b expression, less shedding of CD62L and a lack of shape change activation compared with WT neutrophils (Fig. S6). Following APAP-ALI *in vivo*, there were fewer circulating *Fpr1*$^{-/-}$ neutrophils and hepatic recruitment was delayed, with lower hepatic numbers during injury compared with WT mice (Fig. 3A,B; Fig. S7A–E). Neutrophil hepatic recruitment was delayed but not prevented, likely as a result of system redundancy and other factors, such as cytokine and DNA binding to neutrophil receptors.[76–79] As well as being fewer in number, circulating and hepatic *Fpr1*$^{-/-}$ neutrophil activation was reduced during APAP-ALI, evidenced by lower surface myeloperoxidase (MPO) and decreased CD62L shedding (Fig. 3C–J; Fig. S7D).

Reducing neutrophil activation during APAP-ALI reduces hepatic injury and repair

Fpr1$^{-/-}$-reduced neutrophil activation was associated with decreased hepatic damage early in APAP-ALI. At 16 h post APAP-ALI, weight loss and clinical severity were lower, along with serum biomarkers of hepatic injury (ALT and GLDH), and hepatic necrosis (Fig. 4A–D). Hepatic necrosis and clinical severity remained decreased 24 h post APAP-ALI (Fig. 4C–E), a time of transition from injury resolution to repair in the WT APAP-ALI model (Fig. S1A–E).

Importantly, there were no differences in hepatic parameters in APAP control-treated (healthy) *Fpr1*$^{-/-}$ and WT mice, including liver:body weight, and hepatic serum markers (ALT, AST, ALP, and GLDH) (Fig. S8A and B). There was also no significant difference in hepatic Cyp2e1 or in baseline neutrophil numbers (Fig. S8D and E).

Hepatic repair was also affected in mice that lacked FPR1-mediated neutrophil activation and recruitment. There was a lack of necrosis area improvement between 24 h and 48 h in *Fpr1*$^{-/-}$ mice compared with a 40% reduction in WT mice (Fig. 4B–D). Therefore, both AT7519-mediated neutrophil depletion and preventing FPR1 neutrophil activation were associated with reduced hepatic repair in APAP-ALI. Neutrophil-damaging effects are well documented,[12,51] but no studies have yet assessed the effects of neutrophil modulation during both injury and repair. There are several mechanisms by which neutrophils have been implicated in repair,[13,15,55] although this information is limited in APAP-ALI.

Late neutrophil depletion reduces hepatic proliferation, growth factors, and angiogenesis

All previous studies using neutrophil depletion in APAP-ALI were either initiated before tissue injury or during early injury (≤6 h), even those assessing repair time points.[21] Inducing neutrophil depletion after the onset of injury allows the time-isolated role of neutrophils in repair to be investigated with greater confidence.

Reduced hepatic repair following late neutrophil depletion was further illustrated by elevated hepatic transcripts associated with cell cycle arrest and cell death: (Id1 fold change [FC] 2.139, p = 0.0002; Bax FC 1.35, p = 0.03) and downregulated cellular proliferation transcripts (Mcm5 FC -1.6, p = 0.043; Mik6 FC -6.322, p = 0.002) (Fig. 5A–D). This altered transcript expression was complemented by altered protein expression, with increased hepatic CC3 and reduced hepatocyte MCM2 expression, a cellular pre-replication marker (Fig. 5E–G). In addition, following neutrophil depletion, there were decreased transcripts of the hepatic growth factors Socs2 (FC -2.91, p = 0.01), Il21r (FC -1.83, p = 0.002), and Ifnar2 (FC -1.58, p = 0.004), along with Igf1 (FC -1.53, p = 0.0016). Transcripts of angiogenic factors, including vascular endothelial growth factor (VEGF) (vegfa FC -1.48, p = 0.01) and IL6 (il6ra FC -1.54, p = 0.03), associated with tissue repair after APAP-ALI,[56] were also decreased (Fig. 5H), indicating that neutrophils are involved in hepatic angiogenesis, as seen in other conditions.[16]

Reduced neutrophil activation is associated with reduced ECM remodeling during repair initiation

At 24 h post APAP-ALI, a transitional time from injury to repair, livers from *Fpr1*$^{-/-}$ mice, with fewer and less-activated neutrophils, also had several altered gene transcripts compared with WT controls (Fig. 6A,B). The main reductions were seen in angiogenesis, complement signaling, growth factor signaling, and ECM remodeling (Fig. 6C–F). Some of highest FC parameters were growth factor-related transcripts Fosb (FC -1.91, p = 0.012) and Il1r2 (FC -1.93, p = 0.0008), which were reduced along with ECM remodeling-related transcripts (Col12a1, FC -1.99 p = 0.0001; Adamts9, FC -1.44, p = 0.0002; Serpine1 -1.43, p = 0.015). Neutrophils contribute to ECM remodeling-promoting repair in other conditions,[57,58] but this has not been identified in APAP-ALI. Sphk1 (FC -1.59, p = 0.0007), a transcript involved in VEGF signaling,[59] was similarly reduced (Fig. 6G), demonstrating that analogous repair pathways are affected by both reducing neutrophil activation and depletion in APAP-ALI.

Reducing neutrophil number and activity alter immune signaling and monocyte phenotype

Neutrophils orchestrate inflammation and subsequent tissue repair through a variety of pathways, including actively communicating with other immune cells, particularly to coordinate recruitment during the initial response to tissue injury.[13,16,60,61]

Preventing neutrophil FPR1-mediated recruitment reduces monocyte activation during injury

To investigate the impact of reduced neutrophil activation on local inflammation, hepatic NPC populations were assessed by flow cytometry. At 16 h post APAP-ALI, circulating CD45+ cells were not affected by preventing FPR1 neutrophil activation, but hepatic numbers were reduced, as a result, in large part, of reduced neutrophils (Fig. S7A–D). When reducing neutrophil activity, blood and hepatic-infiltrating monocytes were unchanged in number, but hepatic monocytes were less inflammatory, as indicated by higher percentage Ly6C^{lo}[38] (Fig. S9A–F). *Fpr1*$^{-/-}$ mice had higher hepatic f4/80+ macrophages (Fig. S9G), which might be secondary to lower necrosis, given that hepatic macrophage depletion occurs during early APAP-ALI,[62] and that this difference was resolved at 24 h post APAP-ALI (Fig. S9H). *FPR1* mRNA is highly expressed in neutrophils, specifically hepatic neutrophils following APAP-ALI, supported by analysis of available single cell sequencing data.[63] We qualified this further and identified protein expression within hepatic Ly6G+ cells following APAP-ALI (Fig. S10). However,

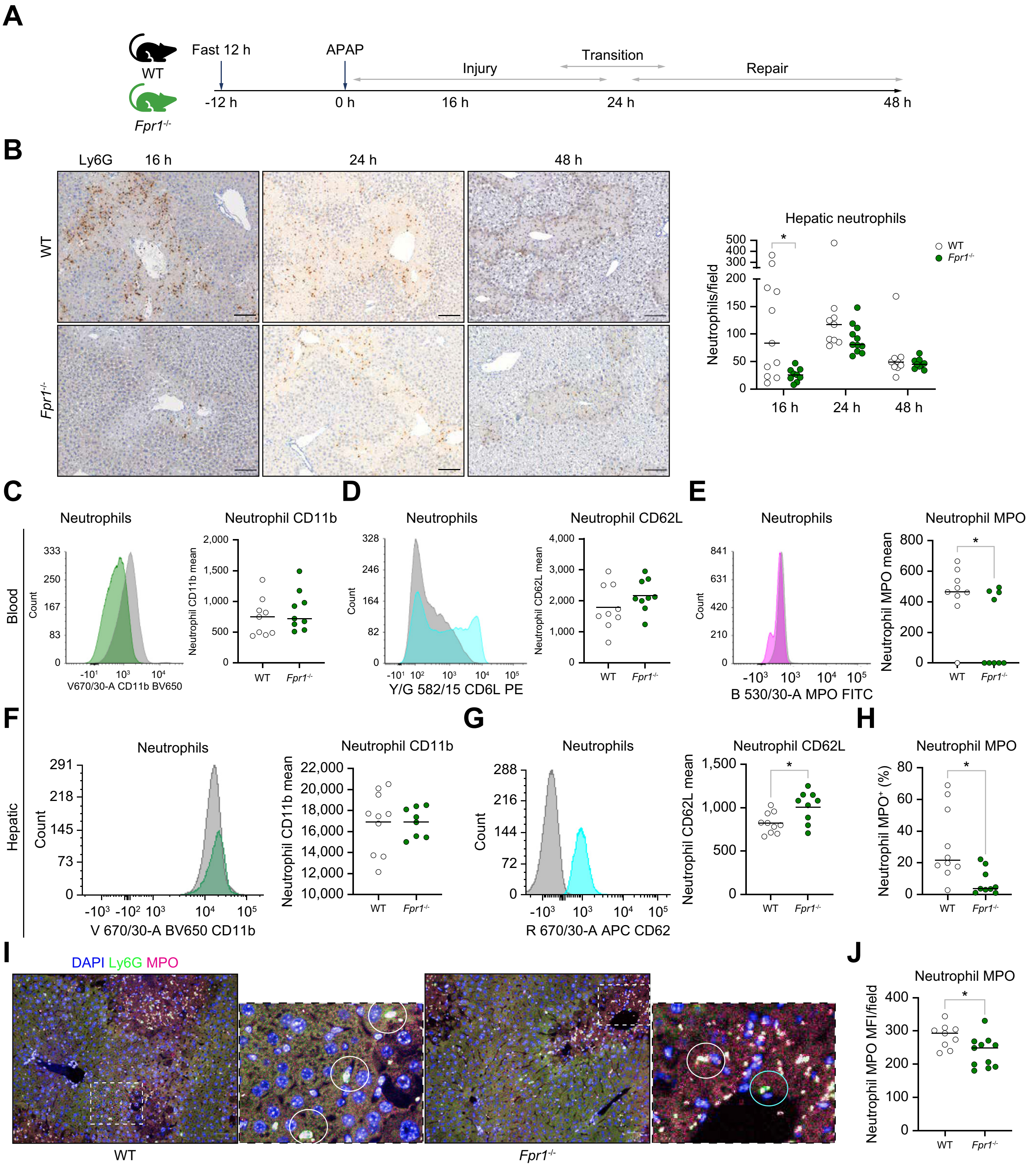

Fig. 3. *Fpr1*$^{-/-}$ neutrophils are less activated with delayed hepatic recruitment. (A) Model schematic. (B) Representative Ly6G-labeled hepatic sections, showing reduced neutrophil recruitment (KS, p = 0.036). (C–E) Representative 16 h blood neutrophil flow cytometry histograms of WT (gray) and *Fpr1*$^{-/-}$ (colored) activation markers and quantification, with reduced *Fpr1*$^{-/-}$ neutrophil surface MPO (*t* test, WC, p = 0.041). (F–H) Hepatic neutrophil activation flow cytometry: (F) CD11b and (G) reduced CD62L shedding (*t* test, p = 0.0134), and (H) decreased surface MPO (*t* test, p = 0.0258). (I) Representative 24 h Ly6G, and MPO-labeled hepatic sections, showing neutrophils with (white circles) and without (blue circle) surface MPO. (J) Decreased *Fpr1*$^{-/-}$ neutrophil MPO (*t* test, p = 0.0214). Scale bars: 100 µm (IHC), 50 µm (IF). FPR1, formylated peptide receptor 1; IF, immunofluorescence; IHC, immunohistochemistry; KS, Kolmogorov–Smirnov; MPO, myeloperoxidase; WC, Welch's correction. In all instances: *p <0.05.

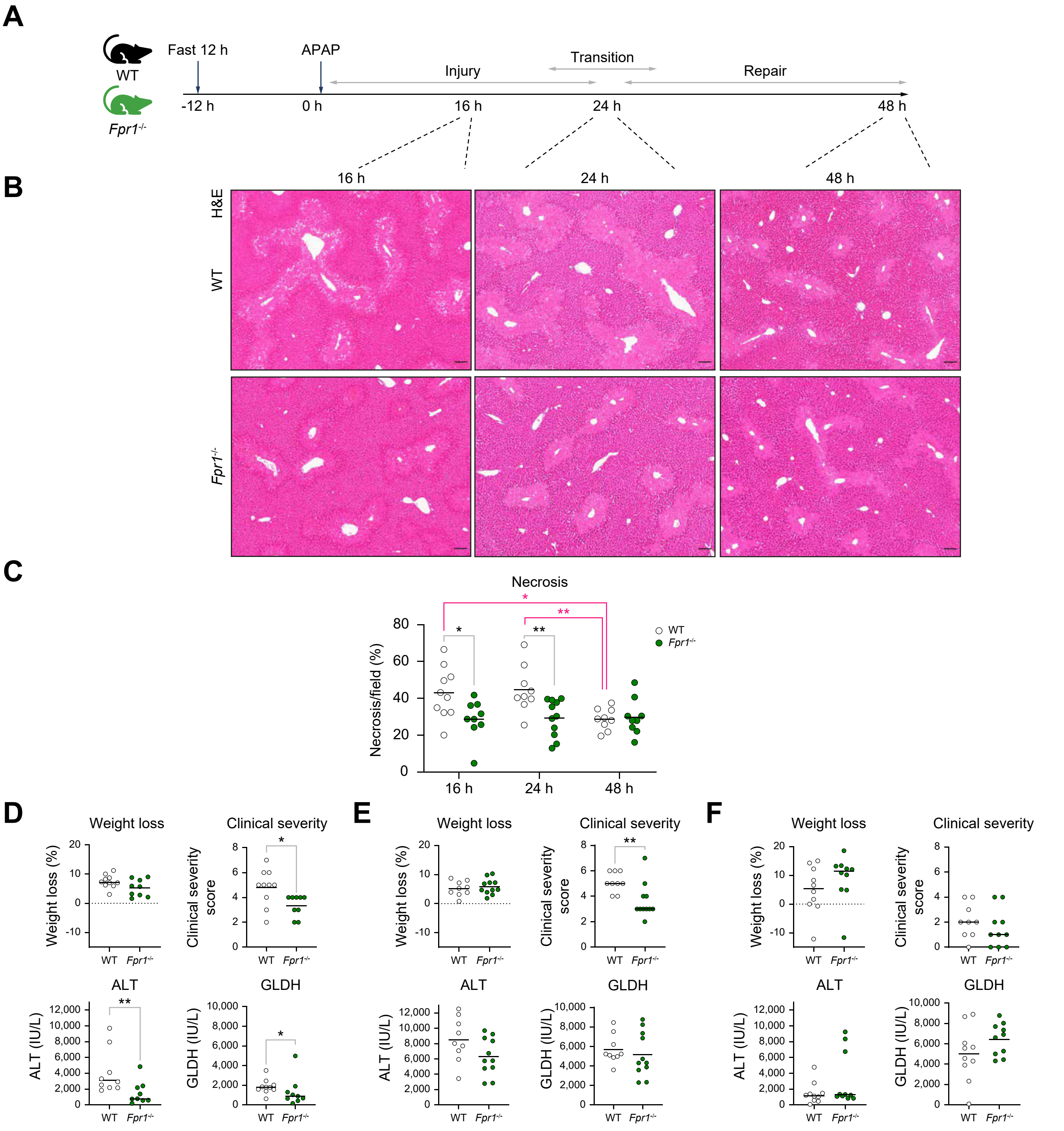

Fig. 4. ***Fpr1*$^{-/-}$ activated neutrophils contribute to both hepatic injury and repair.** (A) Model schematic. (B) Representative H&E hepatic sections showing necrosis. (C) Reduced necrosis in *Fpr1*$^{-/-}$ mice (at 16 h; MW, p = 0.0279, and 24 h; t test, p = 0.0072) (black asterisks). Resolved WT hepatic necrosis at 16–48 h (ANOVA and Tukey's test; p = 0.0164) and 24–48 h (ANOVA and Tukey's test; p = 0.0078) (pink asterisks), not present in *Fpr1*$^{-/-}$ mice. (D) Lower clinical severity (t test, p = 0.018), ALT (MW, p = 0.0061), and GLDH (KS, p = 0.0258) in *Fpr1*$^{-/-}$ mice during injury. (E) Lower 24-h clinical severity (MW, p = 0.0052). (F) Weight loss (MW, p = 0.2475) and GLDH (t test, p = 0.1594) at 48 h, during repair; n ≥9. Scale bars: 100 µm. ALT, alanine aminotransferase; FPR1, formylated peptide receptor 1; GLDH, glutamate dehydrogenase; KS, Kolmogorov–Smirnov; MW, Mann-Whitney; WT, wild type. In all instances: *p <0.05, **p <0.005.

both hepatic monocytes and macrophages expressed lower transcript levels (Fig. S10) and, thus, some direct effects on these cells cannot be excluded, despite not identifying protein expression.

Depleting neutrophils promotes a proinflammatory monocyte phenotype during repair

Monocyte-derived macrophages can facilitate tissue repair[62] and neutrophils have been shown to direct a reparative

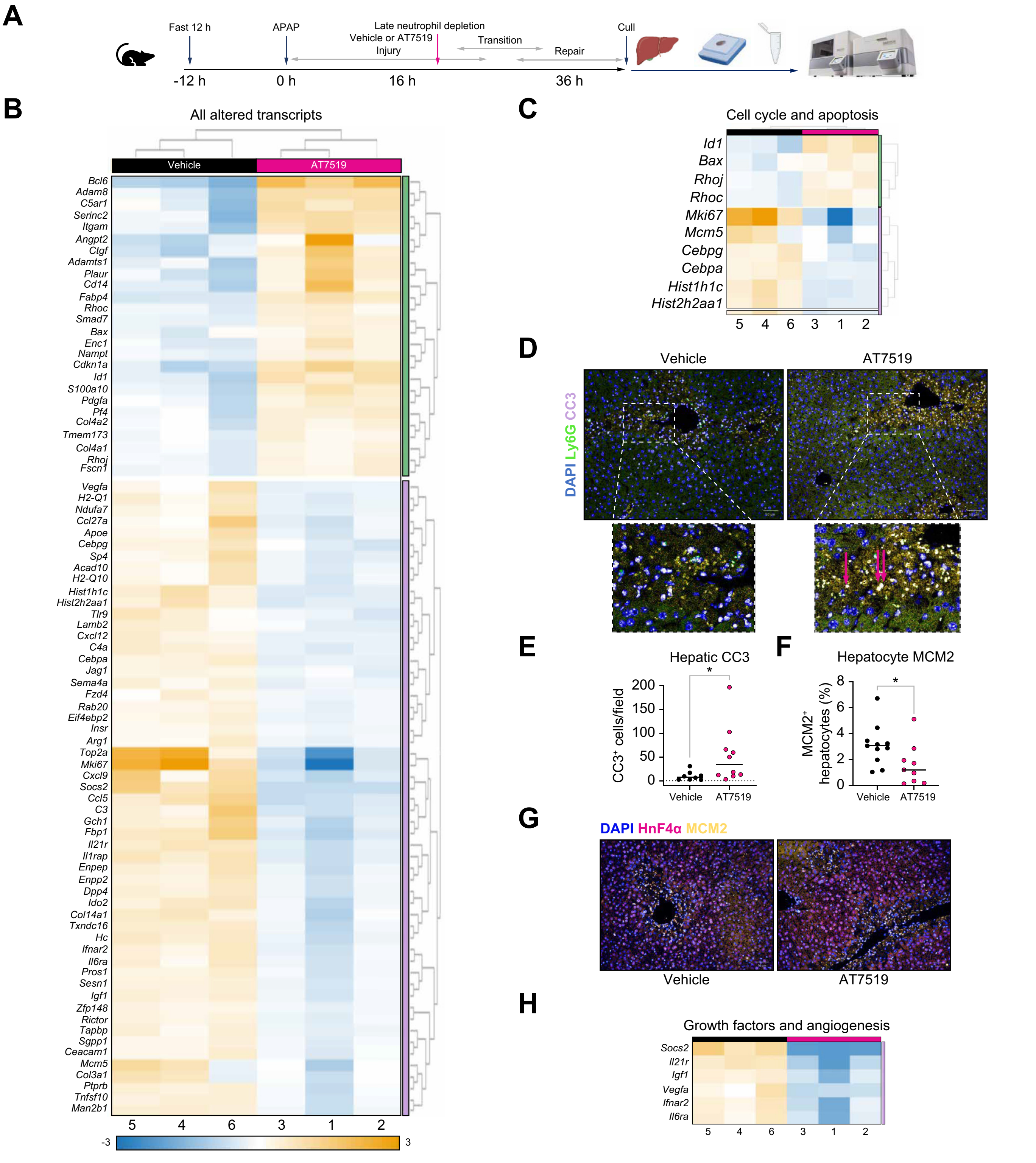

Fig. 5. Depleting hepatic neutrophils reduces hepatic proliferation, growth factors and angiogenesis. (A) Experimental schematic. (B) ROSALIND heatmap of 81 transcripts altered between vehicle and AT7519 neutrophil-depleted livers (FC ≥1.25 or ≤-1.25). (C) Cell cycle-related genes showing upregulated cell death transcripts and downregulated proliferation transcripts. (D) Representative CC3 and Ly6G-labeled hepatic sections and magnifications showing increased CC3 following neutrophil depletion (pink arrows), quantified in (E) (MW, p = 0.015). (F) Decreased percentage of MCM2+ hepatocytes (MW, p = 0.025). (G) Representative MCM2-labeled hepatic sections; n = 10. (H) Reduced hepatic growth factor and angiogenesis-related transcripts after neutrophil depletion; n = 3. Scale bars: 50 µm. FC, fold change; MCM2, minichromosomal maintenance 2; MW, Mann-Whitney. In all instances: *p <0.05.

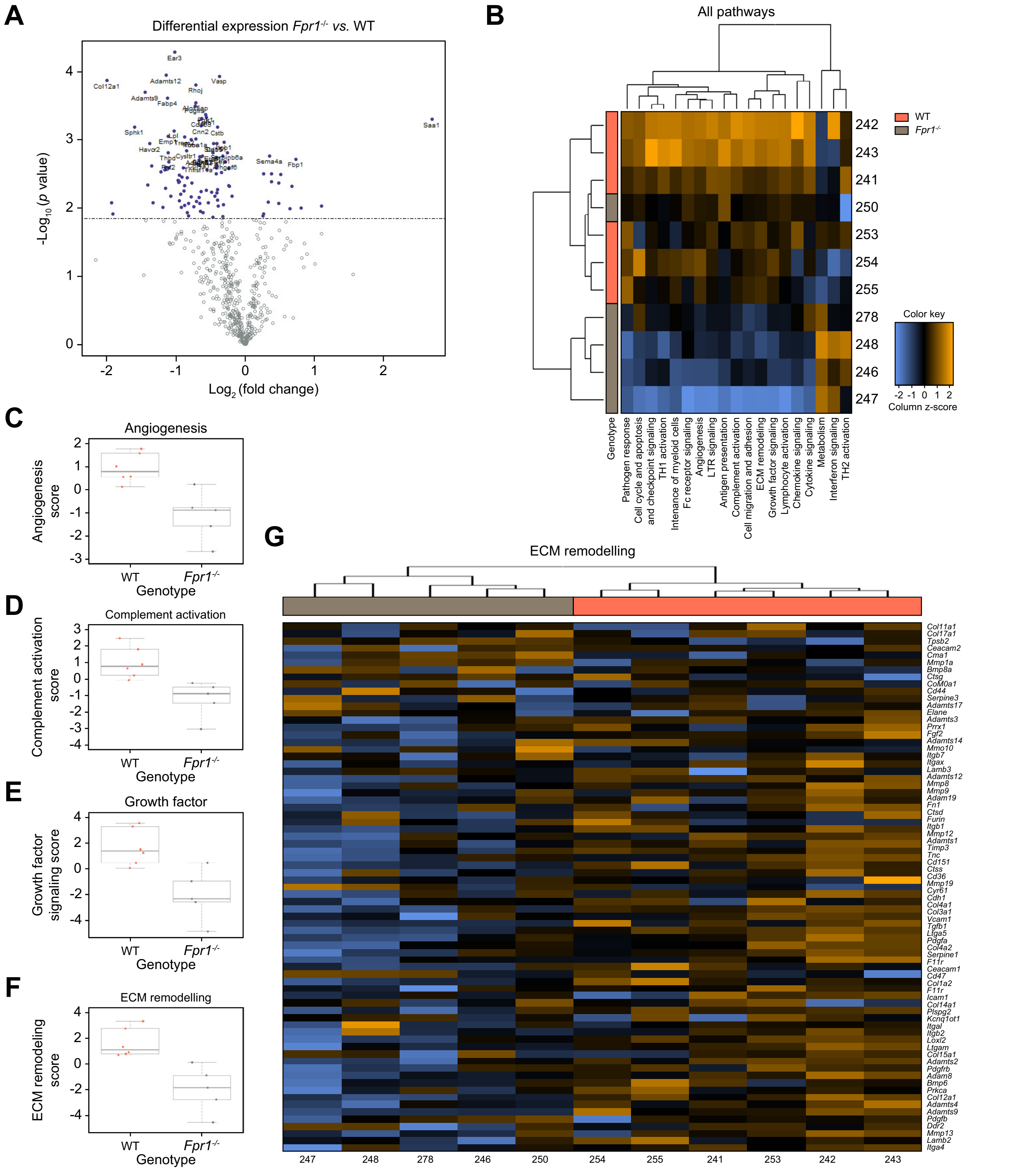

Fig. 6. Preventing FPR1 neutrophil activity reduces ECM remodeling. Graphical representations (nSolver) of hepatic NanoString mouse myeloid panel analysis from WT and *Fpr1*$^{-/-}$ mice 24 h after APAP administration. (A) Volcano plot highlighting *p* <0.05 results between the two groups (purple data points). (B) Heat map of NanoString nSolver pathway analysis. (C–F) nSolver pathway scores from PCA of each covariate: (C) angiogenesis, (D) complement activation, (E) growth factor signaling, and (F) ECM remodeling. (G) Heat map of ECM-related probes: WT (orange), *Fpr1*$^{-/-}$ (gray), and mRNA FC column z score (blue, low; orange, high). Right-hand side shows individual mouse IDs; n ≥5/group. APAP, acetaminophen (paracetamol); ECM, extracellular matrix; FPR1, formylated peptide receptor 1; PCA, principal component analysis; WT, wild type.

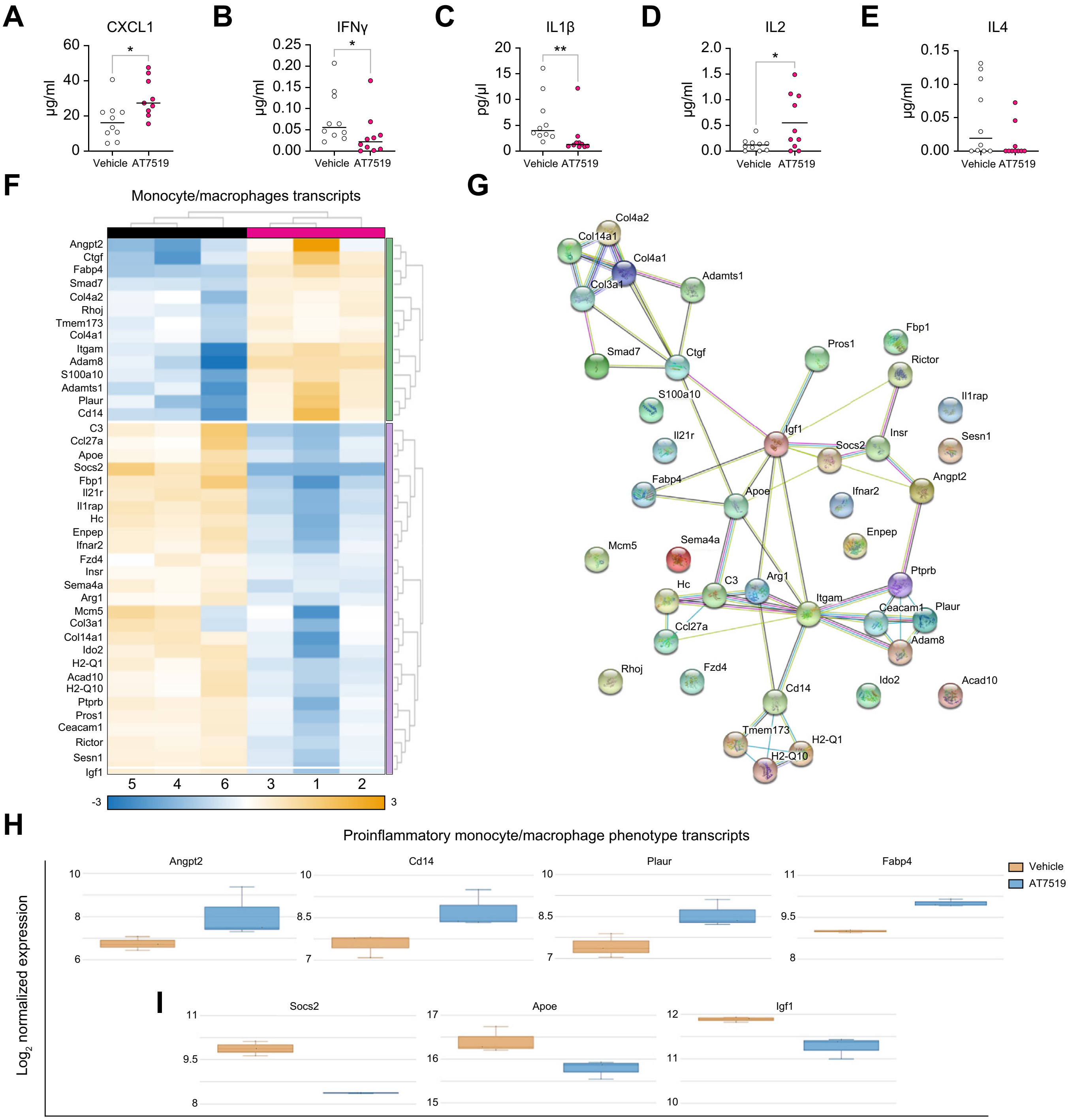

Fig. 7. Depleting neutrophils results in a proinflammatory monocyte phenotype during repair. Results from 36-h APAP-ALI mice with AT7519-neutrophil depletion or vehicle control. (A–E) MSD® multiplexed ELISA hepatic tissue cytokine concentrations. Neutrophil depletion (A) increased KCGRO/CXCL1 (MW, p = 0.01), and decreased (B) IFNγ (MW, p = 0.03), (C) IL1β (KS, p = 0.003), (D) IL2 (WC, p = 0.034), and (E) IL4 (MW, p = 0.062); n = 10 in all instances. (F) ROSALIND heatmap of altered hepatic monocyte/macrophage-related transcripts. (G) NanoString protein known and predicted interactions (nodes represent proteins, edges represent interactions). (H) Upregulated proinflammatory-related transcripts: Angpt2, Cd14, Plaur, and Fabp4; n = 3. (I) Downregulated anti-inflammatory-related transcripts: Socs2, Apoe, and Igf1; n = 3. APAP-ALI, acetaminophen (paracetamol)-induced acute liver injury; CXCL1, C-X-C motif chemokine ligand 1; KS, Kolmogorov–Smirnov; MSD, Meso Scale Discovery; MW, Mann-Witney. In all instances: *p <0.05, **p <0.005.

monocyte phenotype *in vitro*, in other models, and, most recently, in APAP-ALI.[64] We assessed hepatic inflammatory cytokines and monocyte/macrophage phenotypes to investigate how late hepatic neutrophil depletion affected monocyte/macrophage populations and local inflammatory signals (Fig. 7). Depletion resulted in increased C-X-C motif chemokine ligand 1 (CXCL1), a macrophage/monocyte chemoattractant for neutrophils.[65] There was also higher IL2, which is associated with

reduced liver function and recovery in patients with ALI.[66] IFNγ, a cytokine produced by neutrophils,[15] was lower, along with IL1β (Fig. 7A–E), a neutrophil elastase-dependent monocyte-produced cytokine.[67] AT7519 does not deplete monocytes[68] and we confirmed *in vivo* selectivity with no reduction in blood and hepatic monocytes, hepatic eosinophils, and macrophages (Fig. S11). Following neutrophil depletion, 41 altered monocyte and macrophage-related gene transcripts were detected with NanoString myeloid analysis (Fig. 7F). The corresponding translated proteins showed several interconnections related to an inflammatory phenotype assessed using STRING (v11.0b) (Fig. 7G). Several increased transcripts (Angpt2 [FC 3.23, p = 0.008], Cd14 [FC 2.43, p = 0.009], and Plaur [FC 2.27, p = 0.04]) are associated with a proinflammatory monocyte and macrophage phenotype,[69,70] and monocyte/macrophage anti-inflammatory and reparative-associated transcripts (Socs2, Igf1, and Apeo [FC -1.56, p = 0.03])[71,72] were decreased. Macrophage-produced Fabp4 (FC 2.03, p = 0.003), which recruits neutrophils,[73] was also increased following neutrophil depletion (Fig. 7F–I).

Discussion

Neutrophil functions and their impact on local hepatic tissue and cellular communications are dependent on the timing of assessment in APAP-ALI. Our results show that they contribute to both hepatic damage in early stages and repair in late stages of this injury, clarifying previous, apparently contradictory, studies.[21,26]

Our finding of rapid hepatic neutrophil recruitment after APAP-ALI is consistent with other research[21] and, similar to this report, we did not identify a second wave. There is also no second recruitment wave shown in limited publications detailing human circulating neutrophils over time.[9,49] We evidenced persistently increased hepatic numbers and continued activation, consistent with one mouse study documenting elevated neutrophil CD11b at 48 h post injury[49] and some available late human circulating neutrophil data.[9,49] Our WT data highlight some neutrophil differences over time that could be consistent with subsets of these cells. Neutrophil subsets have been defined by individual time point single cell analysis in ALF[63] and in an APAP autoprotection study.[74] Neutrophil heterogeneity is clear in various contexts, but distinct subsets remain controversial.[75–77] We were unable to qualify whether there were discrete time-dependent neutrophil subsets in APAP-ALI or whether recruited neutrophils change *in situ* over time; thus, this requires further investigation, as well as defining its relevance for patients, such as, for example, whether discrete subsets can be targeted.

During injury stages of APAP-ALI, both depleting neutrophils and preventing FPR1-mediated neutrophil activation resulted in diminished hepatic injury. FPR1 ligation is a potent neutrophil activator[18,20,54,78] and preventing this reduced cell shape change, early chemotaxis, and tissue migration. Preventing FPR1 ligation also reduced degranulation, including MPO, which damages tissue[79] and contributes to non-alcohol-related steatohepatitis,[80] but has not previously been associated with APAP-ALI. Hepatic monocytes were also less activated during injury stages, which might be the result of *in situ* neutrophil–monocyte communication or recruitment of an altered monocyte population, given that neutrophils have an essential role in monocyte recruitment and activation, and driving monocyte phenotypes.[61,81] To our knowledge, a neutrophil role for monocyte/macrophage cooperation during APAP-ALI damage stages has not previously been demonstrated.

Inducing neutrophil apoptosis early promoted inflammation resolution and reduced tissue damage, as seen in other models.[45,46,48] This was most likely as result of a lack of proinflammatory neutrophil actions, although might have also been secondary to increasing efferocytosis,[41] given that this promotes an anti-inflammatory macrophage phenotype,[82] which is important in APAP-ALI.[83]

Based on injury time point results alone, reducing neutrophil recruitment, activation, and subsequent hepatic damage would appear a potentially tractable therapeutic target in APAP-ALI, particularly with the horizon of more available specific FPR1 inhibitors and clinical trials in other conditions.[84,85] Indeed, a positive effect has been seen during early APAP-ALI with pharmacological inhibition,[20] and in other conditions.[17,18,43,53] Patients with APAP-ALI have high circulating numbers of activated neutrophils,[5,6] but the positive effect of reducing early neutrophil activity could have detrimental effects for tissue repair or altered sepsis risk during patient recovery.[8,9] Given that *N*-acetylcysteine (NAC) is the standard of care in human medicine,[3] the impact of reducing secondary inflammation with specific FPR1 inhibitors would first need to be assessed alongside NAC, including interrogation of the subsequent repair. Information from prospective longitudinal neutrophil functional studies in humans with APAP-ALI, alongside results of clinical trials in patients with inflammatory bowel disease (EudraCT Number: 2021-000035-31.23), would be important information to incorporate to propose an optimal therapeutic time point for patients. It is also more crucial to identify late presentation treatment options, given that these are currently limited, for example focusing on harnessing or recovering neutrophil reparative and protective functions.

Hepatic neutrophils were increased and remained activated during repair, for longer than previously identified.[45,66–68] A tissue requirement for neutrophils during APAP-ALI repair after their depletion is highlighted here by increased hepatic CXCL1 and Fabp4, which are both are secreted by macrophages to drive neutrophil recruitment.[65,73,86] Hepatocytes also produce CXCL1 to recruit neutrophils, secondary to macrophage TNF signaling.[87] These results are consistent with findings reported by Chuahan *et al.*,[88] which ascertained that hepatic macrophage TNF secretion is important for neutrophil recruitment and subsequent hepatic repair in APAP-ALI. Interestingly, TNF also increases expression of FPR1 on human neutrophils.[88]

FPR1-mediated neutrophil actions are required for APAP-ALI repair, demonstrated by the lack of necrosis improvement at 48 h post injury with reduced neutrophil activity and reduced ECM-related transcripts. Neutrophil-mediated wound healing through ECM remodeling has been demonstrated in skin and peritoneum[57,58] and FPR neutrophil activation has been shown to accelerate healing.[89] Evidencing altered ECM-related transcripts both following neutrophil depletion, and when preventing FRPR1-mediated neutrophil functions, signifies their contribution to ECM remodeling in APAP-ALI and additional mechanistic investigations are warranted.

FPR1 expression is predominantly neutrophilic,[85] which increases with activation[18] and is a key regulator of hepatic recruitment during injury, as shown here and in other models.[53,90] To our knowledge, hepatic cellular protein

expression has not been interrogated and not specifically in APAP-ALI. We showed highest FPR1 expression and localization on hepatic neutrophils, with no parenchymal labeling. Human Protein Atlas datasets show a predominant expression on neutrophils (Tissue Cell Type – FPR1 – The Human Protein Atlas) and our analysis of a mouse APAP-ALF single cell sequencing dataset[63] confirmed highest fold neutrophil expression, but also lower positivity in hepatic monocytes. We recognize that FPR1 is not limited to neutrophils, but, as in other inflamed tissues, the impact on other immune cells and, therefore, their phenotype in the context of APAP-ALI is considered to be small. Given that we also identified a striking effect on monocyte/macrophage phenotype with AT7519-mediated neutrophil depletion, we consider our comparable *Fpr1*$^{-/-}$ results to be neutrophil mediated. Other FPRs were not investigated in our study, because the genetic modulation was FPR1 specific. FPR2 is reported to improve inflammation resolution and is recognized on neutrophils;[91,92] furthermore, mitochondrial formyl peptides can bind FPR2 with similar affinity,[93] and, therefore, binding might have contributed to restored hepatic neutrophil accumulation at 24 and 48 h post APAP. Neutrophil FPR2-mediated functions could be of interest in future studies of APAP-ALI.

Neutrophil-mediated angiogenesis is also important for tissue repair,[15,16] and we identified reductions in angiogenesis transcripts with both methods of neutrophil modulation. Hepatic Sphk1, which regulates angiogenesis through reactive oxygen species (ROS)[59] and is shown to be proinflammatory during early APAP-ALI,[94] is reduced without FPR1-activated neutrophils. VEGF produced by neutrophils, and macrophages following neutrophil communication,[16,95] was reduced without neutrophils, and is important for hepatic regeneration.[56]

We also revealed both damaging and reparative neutrophil functions in APAP-ALI, resolving apparent contradictory studies. Similar controversies once existed for monocytes and macrophages in APAP-ALI, but subpopulations are now accepted, along with divergent time-dependent roles of infiltrating monocyte-derived macrophages in APAP-ALI,[96,97] and their reparative functions have since been harnessed to improve APAP-ALI repair.[98] Elucidating and harnessing neutrophil repair functions to treat patients with APAP-ALI, particularly those found to have abnormal neutrophil functions,[9] is an important future direction.

Late neutrophil depletion was also associated with proinflammatory monocyte/macrophage phenotype transcripts, indicating at least part of the neutrophil reparative function occurs through their positive communications with monocytes and macrophages. These findings complement those of Yang *et al.*,[28] who identified a lack of monocyte/macrophage conversion to a pro-reparative Ly6C^{lo}CX3CR1^{hi} phenotype without neutrophil ROS.

Given our results, the timing of neutrophil interventions and assessments is vital. The rapid, robust, and selective pharmacological depletion of hepatic neutrophils revealed in this study, even at peak inflammation, facilitated interrogation of their impact at different times. Importantly, without the effect of any proceeding modulations, such as those seen with nonspecific depletions[29,99] or before injury modulations.[21,30] AT7519 does not deplete other innate immune cells and does not reduce macrophage numbers,[41,68,100] although reduced proinflammatory macrophage cytokine expression has been reported,[68] which could have contributed to the reduced injury results.

We established that multiple reparative hepatic pathways are affected by reducing neutrophils or their functions; however, there are potentially further neutrophil roles that we have not yet explored. These include phagocytosis of necrotic cellular debris,[10] miRNA production,[101,102] and infection control, particularly given the reduced neutrophil functions of patients with APAP-ALI-induced ALF[8,9] and the higher risk of fatal bacterial infections.[3]

There is limited information about neutrophil activity and function in patients with APAP-ALI. Unfortunately, given the severity of patient illness, and often coagulation complications, hepatic samples are frequently limited to post-transplanted livers, significantly biasing analysis to non-recovery groups. Therefore, circulating neutrophils are the next proxy of measurement in humans. Patients with APAP-ALI have increased circulating neutrophil numbers similar to our mouse model, which normalize by Day 4 of recovery, although no difference between surviving and non-surviving cohorts has been found.[6] Circulating neutrophil CD64, the Fcγ receptor and a marker of neutrophil activation, is elevated in patients with APAP-ALI,[23] but given that this also increases with sepsis, interpretation of functional significance is challenging. Williams *et al.* reported results from three patients recovering from APAP-ALI, detailing elevations in circulating neutrophil ROS, CD11b, and phagocytic capacity during recovery.[74] Cytopenia is a negative risk factor in APAP-ALI[22] and reduced neutrophil activity and phagocytic function were negatively corelated with survival in a small cohort of patients with APAP-ALI.[9] The sparsity of information regarding neutrophil kinetics, maturity, granularity, transcriptional activity, and function in APAP-ALI likely highlights the difficulties associated with capturing patient samples and rapidly analyzing these terminally differentiated cells.

Depicting this duality and time-dependent function of neutrophils in APAP-ALI indicates that, rather than previous studies being contradictory, both lines of investigation are true; neutrophils contribute to both hepatic injury and later resolution and repair. Further investigations to determine pivotal neutrophil functions and specifically reparative subsets, and those promoting known reparative macrophage phenotypes[103] in APAP-ALI are important avenues of continued investigation. Large prospective longitudinal studies with rapid on-site analysis are required to interrogate circulating neutrophil phenotypes and functions in patients. Such studies could establish further neutrophil prognostic biomarkers and potentially define patients requiring intervention, such reducing early neutrophil-damaging functions or rescue of late repair and phagocytic functions.

We do know that circulating neutrophils differ significantly from tissue neutrophils;[104] thus, continued investigations in the mouse model, which provides information otherwise inaccessible in humans,[105] are still required. For example, as mentioned, such work could involve trialing more specific FPR1 inhibitors alongside NAC, as well as further defining neutrophil monocyte cooperation.

Further investigation of this now known time-dependent role in APAP-ALI could not only facilitate development of much needed novel late presentation treatments for this condition, but could also facilitate treatments in other conditions.

Affiliations

[1]Centre for Inflammation Research, Institute for Regeneration and Repair, University of Edinburgh, Edinburgh, UK; [2]Centre for Regenerative Medicine, Institute for Regeneration and Repair, University of Edinburgh, Edinburgh, UK; [3]The Royal (Dick) School of Veterinary Studies and the Roslin Institute, University of Edinburgh, Edinburgh, UK; [4]Mass Spectrometry Core, Edinburgh Clinical Research Facility, Centre for Cardiovascular Sciences, Queen's Medical Research Institute, University of Edinburgh, Edinburgh, UK; [5]IRR Imaging Facility, Institute for Regeneration and Repair, University of Edinburgh, Edinburgh, UK

Abbreviations

ALF, acute liver failure; ALI, acute liver injury; ALP alkaline phosphatase; ALT, alanine aminotransferase; APAP-ALI, paracetamol-induced acute liver injury; APAP, acetaminophen (paracetamol); AST, aspartate aminotransferase; CC3, cleaved caspase 3; CDKI, cyclin-dependent kinase inhibitor; CV, central vein; CXCL, C-X-C motif chemokine ligand; Cyp2e1, cytochrome P450 2E1; DAB, 3,3′-diaminobenzidine; DAMP, damage-associated molecular pattern; ECM, extracellular matrix; EGTA, ethylene glycol-bis(β-aminoethyl ether)-N,N,N′,N′-tetraacetic acid); ES, effect size; FACS, fluorescence-activated cell sorting; FC, fold change; FCS, fetal calf serum; FFPE, formalin-fixed paraffin-embedded; FPR1, formylated peptide receptor 1; GLDH, glutamate dehydrogenase; HBSS, Hank's buffered salt solution; KS, Kolmogorov–Smirnov; LC, liquid chromatography; MBP, major basic protein; MCL1, myeloid cell leukemia factor 1; MCM, minichromosomal maintenance; MPO, myeloperoxidase; MS, mass spectrometry; MSD, Meso Scale Discovery; MW, Mann-Whitney; NAC, N-acetylcysteine; NPC, nonparenchymal cells; PAF, platelet-activating factor; PBST, PBS 0.1% Tween 20; PCA, principal component analysis; PFA, paraformaldehyde; RBC, red blood cell; ROS, reactive oxygen species; RT, room temperature; RT, room temperature; VEGF, vascular endothelial growth factor; WC, Welch's correction; WT, wild type.

Financial support

This study received the following financial support: Wellcome Trust [108906/Z/15/Z] (JAC), Medical Research Council (MRC) UK grant MR/K013386/1 (AR), MRC Autologous Macrophage Therapy for liver cirrhosis (DPFS grant) MR/M007588/1 (RA), MRC Defining The Regenerative Capacity Of Ductular Cells From Non-transplantable Human Liver grant MR/P016839/1 (TYK), MRC Macrophage Therapy for Acute Liver Failure (MAIL) grant MR/T044802/1 (MA, MC), MRC UKRMP Exploiting In Silico Modelling to Address the Translational Bottleneck in Regenerative Medicine Safety grant MR/T015489/1 (CAH), and MRC Research Grant MR/X019314/1 (CDL).

Conflicts of interest

There was no competing interests at the time of the experiments; however, LC, PSL, and SJF are shareholders of Resolution Therapeutics Ltd. a macrophage cell therapy developer. SJF is a scientific adviser for, and LC is an employee of, Resolution Therapeutics. AMK is a consultant for Resolution Therapeutics.

Please refer to the accompanying ICMJE disclosure forms for further details.

Authors' contributions

Conceptualization: JAC, AGR, SJF. Methodology: JAC, AGR, SJF, LC, PSL (statistics; JAC, AGR). Investigation: JAC, PMDP, EL, NG, MO, GR, JPS, NZH, RA, TYM, MA, CAH, MC, AJF, CTR, AMK, MV. Visualization: JAC, EL, NZH, PSL, LC, CDL, DAD, AGF, SJF. Funding acquisition: JAC, AGR, SJF. Project administration: JAC. Supervision: AGR, SJF. Writing – original draft: JAC. Writing – review & editing: JAC, PMDP, LC, PSL, AGR, SJF. All authors have read and agreed to the published version of the manuscript.

Data availability statement

All data, code, and materials used in the analysis are available upon request. The NanoString datasets generated and/or analyzed during the current study are available in the Edinburgh DataShare repository (https://doi.org/10.7488/ds/7835 and https://doi.org/10.7488/ds/7836), and mass spectrometry data are available at https://doi.org/10.7488/ds/7686. Additional datasets are also available from the corresponding author on reasonable request. Requests for materials should be sent to the corresponding author.

Acknowledgements

The authors thank the Central Bioresearch Services, University of Edinburgh, for expertise with animal experimentation and sample collection. They thank Astex Pharmaceuticals for the donation of AT7519 and d8-AT7519. The authors acknowledge the IRR flow cytometry and cell sorting facility and the IRR Imaging facility. The authors acknowledge the Mass Spectrometry Core Edinburgh Clinical Research Facility with data collected on the AB SCIEX QTRAP 5500 instrument (RRID:SCR_020517). The authors acknowledge support from NanoString, particularly Bryan Serrels for data analysis assistance, and thank Alison Munro (Host and Tumour Profiling Unit) for NanoString plate processing.

Supplementary data

Supplementary data to this article can be found online at https://doi.org/10.1016/j.jhepr.2025.101417.

References

Author names in bold designate shared co-first authorship

[1] Ostapowicz G, Fontana RJ, Schiodt FV, et al. Results of a prospective study of acute liver failure at 17 tertiary care centers in the United States. Ann Intern Med 2002;137:947–954.

[2] Reuben A, Tillman H, Fontana RJ, et al. Outcomes in adults with acute liver failure between 1998 and 2013: an observational cohort study. Ann Intern Med 2016;164:724–732.

[3] Stravitz RT, Fontana RJ, Karvellas C, et al. Future directions in acute liver failure. Hepatology 2023;78:1266–1289.

[4] Karvellas CJ, Leventhal TM, Rakela JL, et al. Outcomes of patients with acute liver failure listed for liver transplantation: a multicenter prospective cohort analysis. Liver Transplant 2023;29:318–330.

[5] Agrawal T, Maiwall R, Rajan V, et al. Higher circulating natural killer cells and lower lactate levels at admission predict spontaneous survival in non-acetaminophen induced acute liver failure. Clin Immunol 2021;231:108829.

[6] **Moore JK**, **MacKinnon AC**, Man TY, et al. Patients with the worst outcomes after paracetamol (acetaminophen)-induced liver failure have an early monocytopenia. Aliment Pharmacol Ther 2017;45:443–454.

[7] McGill MR, Shapre MR, Williams CD, et al. The mechanism underlying acetaminophen-induced hepatotoxicity in humans and mice involves mitochondrial damage and nuclear DNA fragmentation. J Clin Invest 2012;122:1574–1583.

[8] Clapperton M, Rolando N, Sandoval L, et al. Neutrophil superoxide and hydrogen peroxide production in patients with acute liver failure. Eur J Clin Invest 1997;27:164–168.

[9] Taylor NJ, Nishtala A, Manakkat Vijay GK, et al. Circulating neutrophil dysfunction in acute liver failure. Hepatology 2013;57:1142–1152.

[10] Ng LG, Ostuni R, Hidalgo A. Heterogeneity of neutrophils. Nat Rev Immunol 2019;19:255–265.

[11] **Xie X**, **Shi Q**, Wu P, et al. Single-cell transcriptome profiling reveals neutrophil heterogeneity in homeostasis and infection. Nat Immunol 2020;21:1119–1133.

[12] Soehnlein O, Steffens S, Hidalgo A, Weber C. Neutrophils as protagonists and targets in chronic inflammation. Nat Rev Immunol 2017;17:248–261.

[13] **De Oliveira S**, **Rosowski EE**, Huttenlocher A. Neutrophil migration in infection and wound repair: going forward in reverse. Nat Rev Immunol 2016;16:378–391.

[14] Butin-Israeli V, Bui TM, Wiesolek HL, et al. Neutrophil-induced genomic instability impedes resolution of inflammation and wound healing. J Clin Invest 2019;129:712–726.

[15] Rizo-Téllez SA, Filep JG. Beyond host defense and tissue injury: the emerging role of neutrophils in tissue repair. Am J Physiology-Cell Physiol 2024;326:C661–C683.

[16] Oliveira-Costa KM, Menezes GB, Paula Neto HA. Neutrophil accumulation within tissues: a damage x healing dichotomy. Biomed Pharmacother 2022;145:112422.

[17] **Leslie J**, **Millar BJM**, Del Carpio Pons A, et al. FPR-1 is an important regulator of neutrophil recruitment and a tissue-specific driver of pulmonary fibrosis. JCI Insight 2020;5:e125937.

[18] Dorward DA, Lucas CD, Chapman GB, et al. The role of formylated peptides and formyl peptide receptor 1 in governing neutrophil function during acute inflammation. Am J Pathol 2015;185:1172–1184.

[19] **Marques PE**, **Oliveira AG**, Pereira RV, et al. Hepatic DNA deposition drives drug-induced liver injury and inflammation in mice. Hepatology 2015;61:348–360.
[20] Marques PE, Amaral SS, Pires DA, et al. Chemokines and mitochondrial products activate neutrophils to amplify organ injury during mouse acute liver failure. Hepatology 2012;56:1971–1982.
[21] Guo H, Chen S, Xie M, et al. The complex roles of neutrophils in APAP-induced liver injury. Cell Prolif 2021;54:e13040.
[22] Mehrpour O, Saeedi F, Hadianfar A, et al. Prognostic factors of acetaminophen exposure in the United States: an analysis of 39,000 patients. Hum Exp Toxicol 2021;40:S814–S825.
[23] Abeles RD, Taylor NJ, Vijay GKM, et al. Neutrophil CD64 expression is elevated in acetaminophen-induced acute liver failure. Am J Respir Crit Care Med 2012;186:1058–1059.
[24] Liu Z, Han D, Gunawan B, Kaplowitz N. Neutrophil depletion protects against murine acetaminophen hepatotoxicity. Hepatology 2006;43:1220–1230.
[25] Ishida Y, Kondo T, Kimura A, et al. Opposite roles of neutrophils and macrophages in the pathogenesis of acetaminophen-induced acute liver injury. Eur J Immunol 2006;36:1028–1038.
[26] **Wu H**, **Guo C**, Liu Z, et al. Neutrophils exacerbate acetaminophen-induced liver injury by producing cytotoxic interferon-γ. Int Immunopharmacol 2023;123:110734.
[27] Chauhan A, Sheriff L, Hussain MT, et al. The platelet receptor CLEC-2 blocks neutrophil mediated hepatic recovery in acetaminophen induced acute liver failure. Nat Commun 2020;11:1–12.
[28] Yang W, Tao Y, Wu Y, et al. Neutrophils promote the development of reparative macrophages mediated by ROS to orchestrate liver repair. Nat Commun 2019;10:1076.
[29] Cover C, Liu J, Farhood A, et al. Pathophysiological role of the acute inflammatory response during acetaminophen hepatotoxicity. Toxicol Appl Pharmacol 2006;216:98–107.
[30] Jaeschke H, Liu J. Neutrophil depletion protects against murine acetaminophen hepatotoxicity: another perspective. Hepatology 2007;45:1588–1589.
[31] Németh T, Sperandio M, Mócsai A. Neutrophils as emerging therapeutic targets. Nat Rev Drug Discov 2020;19:253–275.
[32] Chu D, Dong X, Shi X, et al. Neutrophil-based drug delivery systems. Adv Mater 2018;30:e1706245.
[33] Cartwright JA. Defining the role of neutrophils in paracetamol-induced liver injury and regeneration. University of Edinburgh; 2023.
[34] Gao JL, Lee EJ, Murphy PM. Impaired antibacterial host defense in mice lacking the N-formylpeptide receptor. J Exp Med 1999;189:657–662.
[35] Zhou P, Qian L, Bieszczad CK, et al. Mcl-1 in transgenic mice promotes survival in a spectrum of hematopoietic cell types and immortalization in the myeloid lineage. Blood 1998;92:3226–3239.
[36] Squires MS, Feltell RE, Wallis NG, et al. Biological characterization of AT7519, a small-molecule inhibitor of cyclin-dependent kinases, in human tumor cell lines. Mol Cancer Ther 2009;8:324–332.
[37] Campana L, Starkey Lewis PJ, Pellicoro A, et al. The STAT3–IL-10–IL-6 pathway is a novel regulator of macrophage efferocytosis and phenotypic conversion in sterile liver injury. J Immunol 2018;200:1169–1187.
[38] Cartwright JA, Simpson JP, Homer NZM, Rossi AG. Analysis of AT7519 as a pro-resolution compound in an acetaminophen-induced mouse model of acute inflammation by UPLC-MS/MS. J Inflamm 2023;20:20.
[39] Perkins JR, Dawes JM, McMahon SB, et al. ReadqPCR and NormqPCR: R packages for the reading, quality checking and normalisation of RT-qPCR quantification cycle (Cq) data. BMC Genomics 2012;13:296.
[40] Hennig C. Fpc: flexible procedures for clustering. https://cran.r-project.org/web/packages/fpc/index.html. [Accessed 8 April 2025].
[41] Lucas C, Dorward DA, Tait MA, et al. Downregulation of Mcl-1 has anti-inflammatory pro-resolution effects and enhances bacterial clearance from the lung. Mucosal Immunol 2014;7:857–868.
[42] Cohen J. Statistical power analysis for the behavioral sciences. 2nd ed). Hillsdale: Lawrence Erlbaum; 1988.
[43] Dorward DA, Lucas CD, Doherty MK, et al. Novel role for endogenous mitochondrial formylated peptide-driven formyl peptide receptor 1 signalling in acute respiratory distress syndrome. Thorax 2017;72:928–936.
[44] Faul F, Erdfelder E, Lang A-G, et al. G*Power: a flexible statistical power analysis program for the social, behavioral, and biomedical sciences. Behav Res Methods 2007;39:175–191.
[45] Kaveh A, Bruton FA, Oremek MEM, et al. Selective Cdk9 inhibition resolves neutrophilic inflammation and enhances cardiac regeneration in larval zebrafish. Development 2022;149:dev199636.
[46] Cartwright JA, Lucas CD, Rossi AG. Inflammation resolution and the induction of granulocyte apoptosis by cyclin-dependent kinase inhibitor drugs. Front Pharmacol 2019;10:1–18.
[47] Pollenus E, Malengier-devlies B, Vandermosten L, et al. Limitations of neutrophil depletion by anti-Ly6G antibodies in two heterogenic immunological models. Immunol Lett 2019;212:30–36.
[48] Dorward DA, Felton JM, Robb CT, et al. The cyclin-dependent kinase inhibitor AT7519 accelerates neutrophil apoptosis in sepsis-related acute respiratory distress syndrome. Thorax 2017;72:182–185.
[49] Williams CD, Lynn M, Sharpe MR, et al. Neutrophil activation during acetaminophen hepatotoxicity and repair in mice and humans. Toxicol Appl Pharmacol 2014;275:122–133.
[50] Moriceau S, Lenoir G, Witko-Sarsat V. In cystic fibrosis homozygotes and heterozygotes, neutrophil apoptosis is delayed and modulated by diamide or roscovitine: evidence for an innate neutrophil disturbance. J Innate Immun 2010;2:260–266.
[51] Kazzi M El, Rayner BS, Chami B, et al. Neutrophil-mediated cardiac damage after acute myocardial infarction: significance of defining a new target cell type for developing cardioprotective drugs. Antioxid Redox Signal 2020;33:689–712.
[52] Dzhagalov I, St. John A, He YW. The antiapoptotic protein Mcl-1 is essential for the survival of neutrophils but not macrophages. Blood 2007;109:1620–1626.
[53] Honda M, Takeichi T, Hashimoto S, et al. Intravital imaging of neutrophil recruitment reveals the efficacy of FPR1 blockade in hepatic ischemia-reperfusion injury. J Immunol 2017;198:1718–1728.
[54] Lind S, Dahlgren C, Holmdahl R, et al. Functional selective FPR1 signaling in favor of an activation of the neutrophil superoxide generating NOX2 complex. J Leukoc Biol 2021:1105–1120.
[55] Mcnamee N, Bowers BE, Campbell EL, et al. Transmigrating neutrophils shape the mucosal microenvironment through localized oxygen depletion to influence resolution of inflammation. Immunity 2013;40:66–77.
[56] Donahower BC, McCullough SS, Hennings L, et al. Human recombinant vascular endothelial growth factor reduces necrosis and enhances hepatocyte regeneration in a mouse model of acetaminophen toxicity. J Pharmacol Exp Ther 2010;334:33–43.
[57] Bastian OW, Koenderman L, Alblas J, et al. Neutrophils contribute to fracture healing by synthesizing fibronectin+ extracellular matrix rapidly after injury. Clin Immunol 2016;164:78–84.
[58] **Fischer A**, **Wannemacher J**, Christ S, et al. Neutrophils direct preexisting matrix to initiate repair in damaged tissues. Immunology 2022;23:518–531.
[59] Lv MH, Li S, Jiang YJ, et al. The Sphkl/SIP pathway regulates angiogenesis via NOS/NO synthesis following cerebral ischemia-reperfusion. CNS Neurosci Ther 2020;26:538–548.
[60] Wang J. Neutrophils in tissue injury and repair. Cell Tissue Res 2018;371:531–539.
[61] Walters N, Zhang J, Rima XY, et al. Analyzing inter-leukocyte communication and migration in vitro: neutrophils play an essential role in monocyte activation during swarming. Front Immunol 2021;12:1–14.
[62] **Zigmond E**, **Samia-Grinberg S**, Pasmanik-Chor M, et al. Infiltrating monocyte-derived macrophages and resident Kupffer cells display different ontogeny and functions in acute liver injury. J Immunol 2014;193:344–353.
[63] Kolodziejczyk AA, Federici S, Zmora N, et al. Acute liver failure is regulated by MYC- and microbiome-dependent programs. Nat Med 2020;26:1899–1911.
[64] Yang W, Tao Y, Wu Y, et al. Neutrophils promote the development of reparative macrophages mediated by ROS to orchestrate liver repair. Nat Commun 2019:1–14.
[65] De Filippo K, Dudeck A, Hasenberg M, et al. Mast cell and macrophage chemokines CXCL1/CXCL2 control the early stage of neutrophil recruitment during tissue inflammation. Blood 2013;121:4930–4937.
[66] Sun Y, Gu J, Liu R, et al. IL-2/IL-6 ratio correlates with liver function and recovery in acute liver injury patients. Apmis 2019;127:468–474.
[67] Alfaidi M, Wilson H, Daigneault M, et al. Neutrophil elastase promotes interleukin-1β secretion from human coronary endothelium. J Biol Chem 2015;290:24067–24078.
[68] **McHugh BJ**, **Stephen J**, Robb CT, et al. Inhibition of cyclin-dependent kinase 9 downregulates cytokine production without detrimentally affecting human monocyte-derived macrophage viability. Front Cel Dev Biol 2022;10:905315.
[69] **Krausz S**, **Garcia S**, Ambarus CA, et al. Angiopoietin-2 promotes inflammatory activation of human macrophages and is essential for murine experimental arthritis. Ann Rheum Dis 2012;71:1402–1410.

[70] **Ogawa Y**, **Imajo K**, Yoneda M, et al. Soluble CD14 levels reflect liver inflammation in patients with nonalcoholic steatohepatitis. PLoS ONE 2013;8:e65211.
[71] **Li S**, **Han S**, **Jin K**, et al. Socs2 suppresses inflammation and apoptosis during nash progression through limiting NF-κB activation in macrophages. Int J Biol Sci 2021;17:4165–4175.
[72] Li J, Pan Y, Li D, et al. Urokinase-type plasminogen activator receptor is required for impairing toll-like receptor 7 signaling on macrophage efferocytosis in lupus. Mol Immunol 2020;127:38–45.
[73] Liang X, Gupta K, Quintero JR, et al. Macrophage FABP4 is required for neutrophil recruitment and bacterial clearance in *Pseudomonas aeruginosa* pneumonia. FASEB J 2019;33:3562–3574.
[74] **Yu L**, **Yan J**, Zhan Y, et al. Single-cell RNA sequencing reveals the dynamics of hepatic non-parenchymal cells in autoprotection against acetaminophen-induced hepatotoxicity. J Pharm Anal 2023;13:926–941.
[75] Deniset JF, Kubes P. Neutrophil heterogeneity: bona fide subsets or polarization states? J Leukoc Biol 2018:1–10.
[76] **Huang C**, **Fan X**, Shen Y, et al. Neutrophil subsets in noncancer liver diseases: cellular crosstalk and therapeutic targets. Eur J Immunol 2023;53:e2250324.
[77] Christoffersson G, Vågesjö E, Vandooren J, et al. VEGF-A recruits a proangiogenic MMP-9-delivering neutrophil subset that induces angiogenesis in transplanted hypoxic tissue. Blood 2012;120:4653–4662.
[78] Zhang Q, Raoof M, Chen Y, et al. Circulating mitochondrial DAMPs cause inflammatory responses to injury. Nature 2010;464:104–107.
[79] **Tiyerili V**, **Camara B**, Becher MU, et al. Neutrophil-derived myeloperoxidase promotes atherogenesis and neointima formation in mice. Int J Cardiol 2016;204:29–36.
[80] Rensen SS, Slaats Y, Nijhuis J, et al. Increased hepatic myeloperoxidase activity in obese subjects with nonalcoholic steatohepatitis. Am J Pathol 2009;175:1473–1482.
[81] Soehnlein O, Zernecke A, Eriksson EE, et al. Neutrophil secretion products pave the way for inflammatory monocytes. Blood 2008;112:1461–1471.
[82] **Angsana J**, **Chen J**, Liu L, et al. Efferocytosis as a regulator of macrophage chemokine receptor expression and polarization. Eur J Immunol 2016;46:1592–1599.
[83] Triantafyllou E, Pop OT, Possamai LA, et al. MerTK expressing hepatic macrophages promote the resolution of inflammation in acute liver failure. Gut 2018;67:333–347.
[84] Murphy CK, Dixit B, Oleson FB, et al. Development of ADS051, an oral, gut-restricted, small molecule neutrophil modulator for the treatment of neutrophil-mediated inflammatory diseases. FEBS Open Bio 2023;13:1434–1446.
[85] McAllister MJ, Hall R, Whelan RJ, et al. Formylated peptide receptor-1 (FPR1) mediated gut inflammation as a therapeutic target in inflammatory bowel disease. Crohns Colitis 2024;360(6):otae003.
[86] **Scapini P**, **Morini M**, Tecchio C, et al. CXCL1/macrophage inflammatory protein-2-induced angiogenesis in vivo is mediated by neutrophil-derived vascular endothelial growth factor-A. J Immunol 2004;172:5034–5040.
[87] **Su L**, **Li N**, Tang H, et al. Kupffer cell-derived TNF-α promotes hepatocytes to produce CXCL1 and mobilize neutrophils in response to necrotic cells. Cell Death Dis 2018;9:323.
[88] O'Flaherty JT, Rossi AG, Redman JF, et al. Tumor necrosis factor-alpha regulates expression of receptors for formyl-methionyl-leucyl-phenylalanine, leukotriene B4, and platelet-activating factor. Dissociation from priming in human polymorphonuclear neutrophils. J Immunol 1991;147:3842–3847.
[89] Liu M, Chen K, Yoshimura T, et al. Formylpeptide receptors mediate rapid neutrophil mobilization to accelerate wound healing. PLoS ONE 2014;9:e90613.
[90] Mcdonald B, Pittman K, Menezes GB, et al. Intravascular danger signals guide neutrophils to sites of sterile inflammation. Science 2011;330:362–366. 1979.
[91] Ye RD, Boulay F, Wang JM, et al. International union of basic and clinical pharmacology. LXXIII. Nomenclature for the formyl peptide receptor (FPR) family. Pharmacol Rev 2009;61(2):119–161.
[92] Lee C, Han J, Jung Y. Formyl peptide receptor 2 is an emerging modulator of inflammation in the liver. Exp Mol Med 2023;55:325–332.
[93] Rabiet MJ, Huet E, Boulay F. Human mitochondria-derived N-formylated peptides are novel agonists equally active on FPR and FPRL1, while *Listeria monocytogenes*-derived peptides preferentially activate FPR. Eur J Immunol 2005;35:2486–2495.
[94] **Li L**, **Wang H**, Zhang J, et al. SPHK1 deficiency protects mice from acetaminophen-induced ER stress and mitochondrial permeability transition. Cell Death Differ 2020;27:1924–1937.
[95] Tecchio C, Cassatella MA. Neutrophil-derived cytokines involved in physiological and pathological angiogenesis. Chem Immunol Allergy 2014;99:123–137.
[96] Holt MP, Cheng L, Ju C. Identification and characterization of infiltrating macrophages in acetaminophen-induced liver injury. J Leukoc Biol 2008;84:1410–1421.
[97] You Q, Holt M, Yin H, et al. Role of hepatic resident and infiltrating macrophages in liver repair after acute injury. Biochem Pharmacol 2013;86:836–843.
[98] Lewis PS, Campana L, Aleksieva N, et al. Alternatively activated macrophages promote resolution of necrosis following acute liver injury. J Hepatol 2020;73:1–12.
[99] Williams CD, Jaeschke H. Role of innate and adaptive immunity during drug-induced liver injury. Toxicol Res (Camb) 2012;1:161–170.
[100] Hoodless LJ, Lucas CD, Duffin R, et al. Genetic and pharmacological inhibition of CDK9 drives neutrophil apoptosis to resolve inflammation in zebrafish in vivo. Sci Rep 2016;5:36980.
[101] **Calvente CJ**, **Tameda M**, Johnson CD, et al. Neutrophils contribute to spontaneous resolution of liver inflammation and fibrosis via microRNA-223. J Clin Invest 2019;129:4091.
[102] He YY, Feng DD, Li MM, et al. Hepatic mtDNA-TLR9-microRNA-223 forms a negative feedback loop to limit neutrophil over-activation and acetaminophen hepatotoxicity. Hepatology 2017;66:220–234.
[103] Candela ME, Addison M, Aird R, et al. Cryopreserved human alternatively activated macrophages promote resolution of acetaminophen-induced liver injury in mouse. NPJ Regen Med 2025;10:5.
[104] Palomino-Segura M, Sicilia J, Ballesteros I, et al. Strategies of neutrophil diversification. Nat Immunol 2023;24:575–584.
[105] Jaeschke H, Adelusi OB, Akakpo JY, et al. Recommendations for the use of the acetaminophen hepatotoxicity model for mechanistic studies and how to avoid common pitfalls. Acta Pharm Sin B 2021;11:3740–3755.

Keywords: Hepatic; Paracetamol; Acetaminophen; Formylated-peptide-receptor 1; Monocyte; Macrophage; Inflammation; Extracellular matrix remodeling.
Received 16 July 2024; received in revised form 14 March 2025; accepted 28 March 2025; Available online 11 April 2025

Journal of Hepatology, Volume *7*

Supplemental information

Temporal dichotomy of neutrophil function in acute liver injury and repair

Jennifer A. Cartwright, Philippe M.D. Potey, Eilidh Livingstone, Lara Campana, Philip J. Starkey Lewis, Magdalena E.M. Oremek, Naomi N. Gachanja, Giulia Rinaldi, Rhona E. Aird, Tak Yung Man, Anuruddika J. Fernando, Joanna P. Simpson, Natalie Z.M. Homer, Nicole Barth, Melisande Addison, Candice Ashmore-Harris, Maria Elena Candela, Alastair M. Kilpatrick, Matthieu Vermeren, Calum T. Robb, David A. Dorward, Christopher D. Lucas, Stuart J. Forbes, and Adriano G. Rossi

Temporal dichotomy of neutrophil function in acute liver injury and repair

Jennifer A. Cartwright, Philippe M.D. Potey, Eilidh Livingstone, Lara Campana, Philip J. Starkey Lewis, Magdalena E.M. Oremek, Naomi N. Gachanja, Giulia Rinaldi, Rhona E. Aird, Tak Yung Man, Anuruddika J. Fernando, Joanna P. Simpson, Natalie Z.M. Homer, Nicole Barth, Melisande Addison, Candice Ashmore-Harris, Maria Elena Candela, Alastair M. Kilpatrick, Matthieu Vermeren, Calum T. Robb, David A. Dorward, Christopher D. Lucas, Stuart J. Forbes, Adriano G. Rossi

Table of contents

Supplementary figures……………………………………………………………….…......2

Supplementary methods……………………………………………………………......18

Supplementary tables………………………………………………..…………………18

Supplementary references…………………………….……………………………….23

Supplementary figures

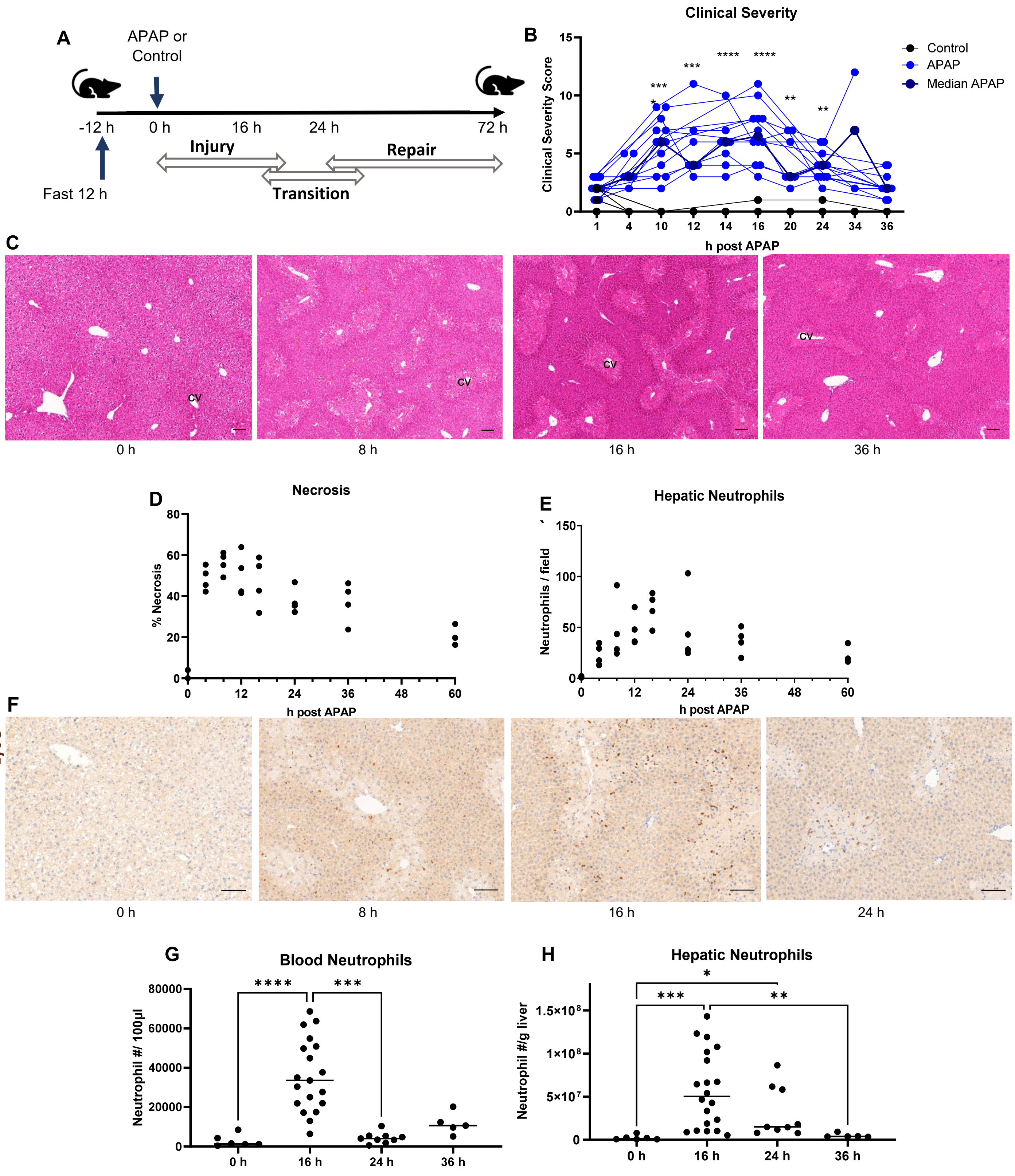

Fig. S1. Hepatic neutrophil accumulation peaks at 16 h in the WT mouse model. (A), Model schematic. **(B),** Total clinical severity score. **(C),** Representative HnE hepatic sections showing necrotic areas around central veins (cv). (**D),** Quantified average % hepatic necrosis. **I,** Average number of neutrophils/hepatic field, quantified from **(F),** Ly6G+ DAB labelled sections, representative images shown. **(G),** Blood neutrophils

significantly different over time, KW $p<0.0001$, Dunn's, 0 v 16, $p=0.0001$, 16 v 24, $p=0.0001$. **(H),** Hepatic neutrophils significantly different over time, KW $p<0.0001$, highest at 16 h post APAP Dunn's, 0 v 16, $p=0.0001$, 0 v 24, $p=0.0256$, 16 v 36, $p=0.0096$. Scale bars 100 µm. KW Kruskal Wallis. n ≥4/group.

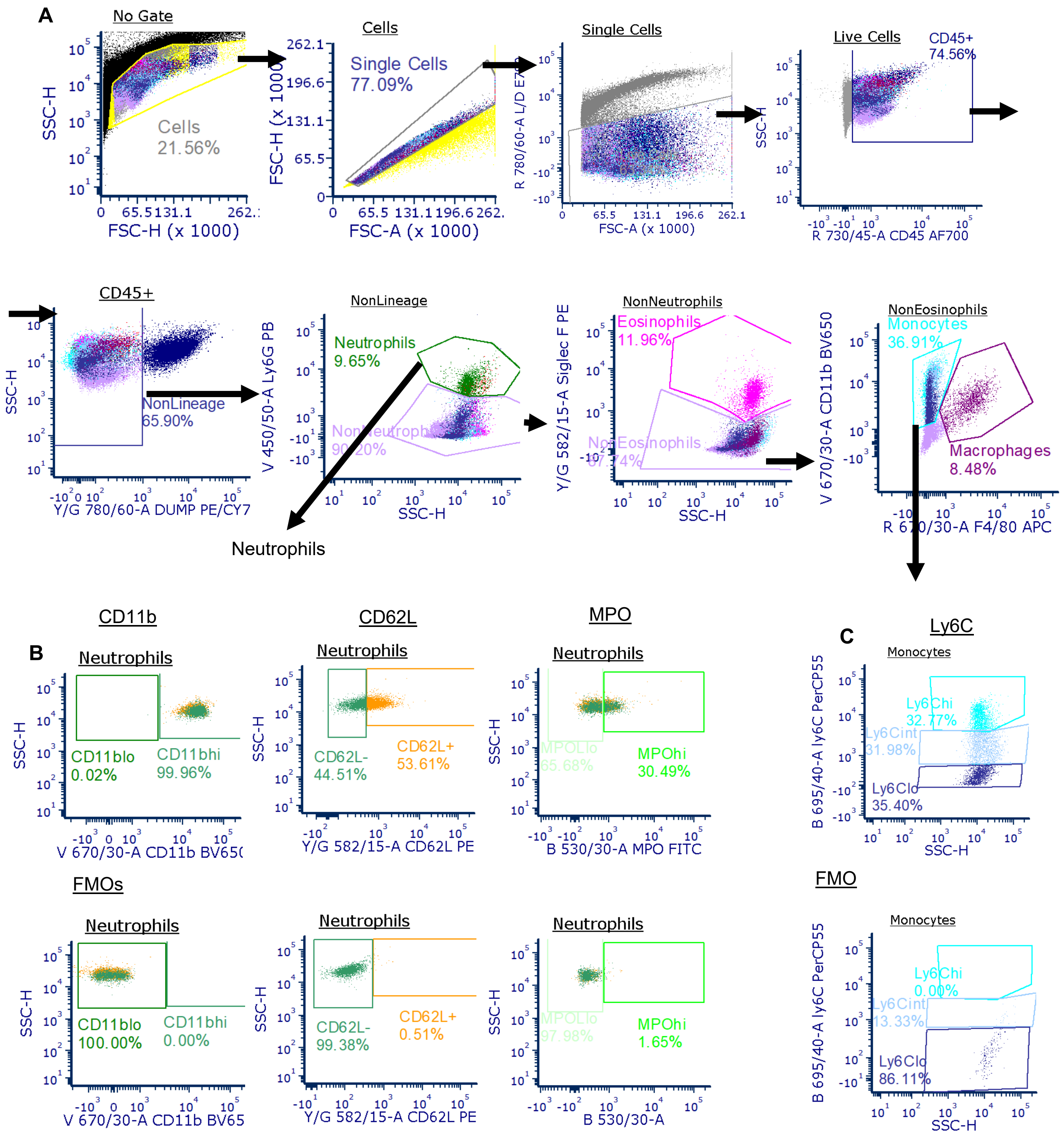

Fig. S2. Hepatic flow cytometry gating strategy for innate immune cells. (A), Representative flow plots of hepatic NPCs demonstrating gating strategy. Each plot is labelled with the parent gate and showing cell populations and gates of interest. Each dot is colored with final gate of interest., e.g. neutrophils are green and Ly6C^{hi} monocytes are cyan. (**B**), Example neutrophil activation markers and corresponding fluorescence minus one control (FMO) below. **(C),** Monocyte Ly6C expression gating and corresponding FMO below.

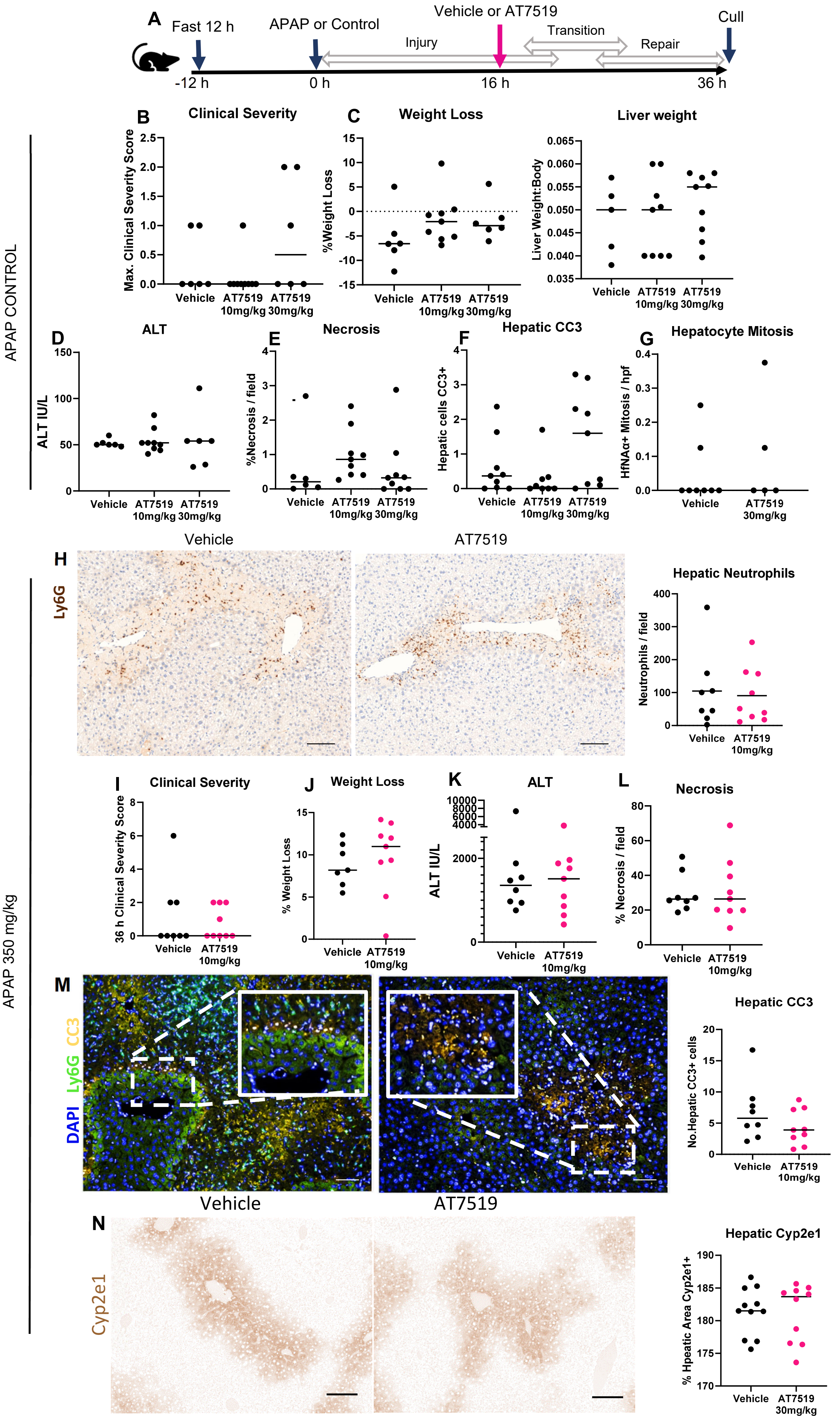
A
Fast 12 h
APAP or Control
Vehicle or AT7519
Injury
Transition
Repair
Cull
-12 h
0 h
16 h
36 h
APAP CONTROL
B
Clinical Severity
Max. Clinical Severity Score
C
Weight Loss
%Weight Loss
Liver weight
Liver Weight:Body
Vehicle
AT7519 10mg/kg
AT7519 30mg/kg
D
ALT
ALT IU/L
E
Necrosis
%Necrosis / field
F
Hepatic CC3
Hepatic cells CC3+
G
Hepatocyte Mitosis
HfNAα+ Mitosis / hpf
APAP 350 mg/kg
H
Vehicle
AT7519
Ly6G
Hepatic Neutrophils
Neutrophils / field
Vehilce
I
Clinical Severity
36 h Clinical Severity Score
J
Weight Loss
% Weight Loss
K
ALT
ALT IU/L
L
Necrosis
% Necrosis / field
M
DAPI Ly6G CC3
Hepatic CC3
No.Hepatic CC3+ cells
N
Cyp2e1
Hepatic Cyp2e1
% Hpeatic Area Cyp2e1+

Fig. S3. AT7519 does not affect healthy livers, and neutrophil depletion is dose dependent

(A), Model schematic. **(B-G),** APAP control treated mice, with no difference in **B,** clinical severity, **C,** % weight loss and liver: body weight following 10 mg/kg or 30 mg/kg AT7519. **D,** Serum ALT, **E,** % hepatic necrosis, **F,** % of hepatic cells positive for CC3 and **G,** Hepatocyte (DAPI+HNF4α+) mitotic events / field also unaltered. **(H-M),** APAP treated mice received vehicle or AT7519 10 mg/kg **H,** Neutrophil labelled hepatic sections and quantification showed no reduction. **I,** 36 h clinical severity, **J,** % weight loss, **K,** Serum ALT, and **L,** % hepatic necrosis unaltered without neutrophil depletion. **M,** Representative hepatic CC3 labelled sections and quantification showing no increase in hepatic CC3. **N,** Representative Cyp2e1 labelled hepatic sections from APAP treated mice with vehicle or 30 mg/kg AT7519, and quantification. IHC scale bars 100 μm. IF scale bars 50 μm. (n≥6).

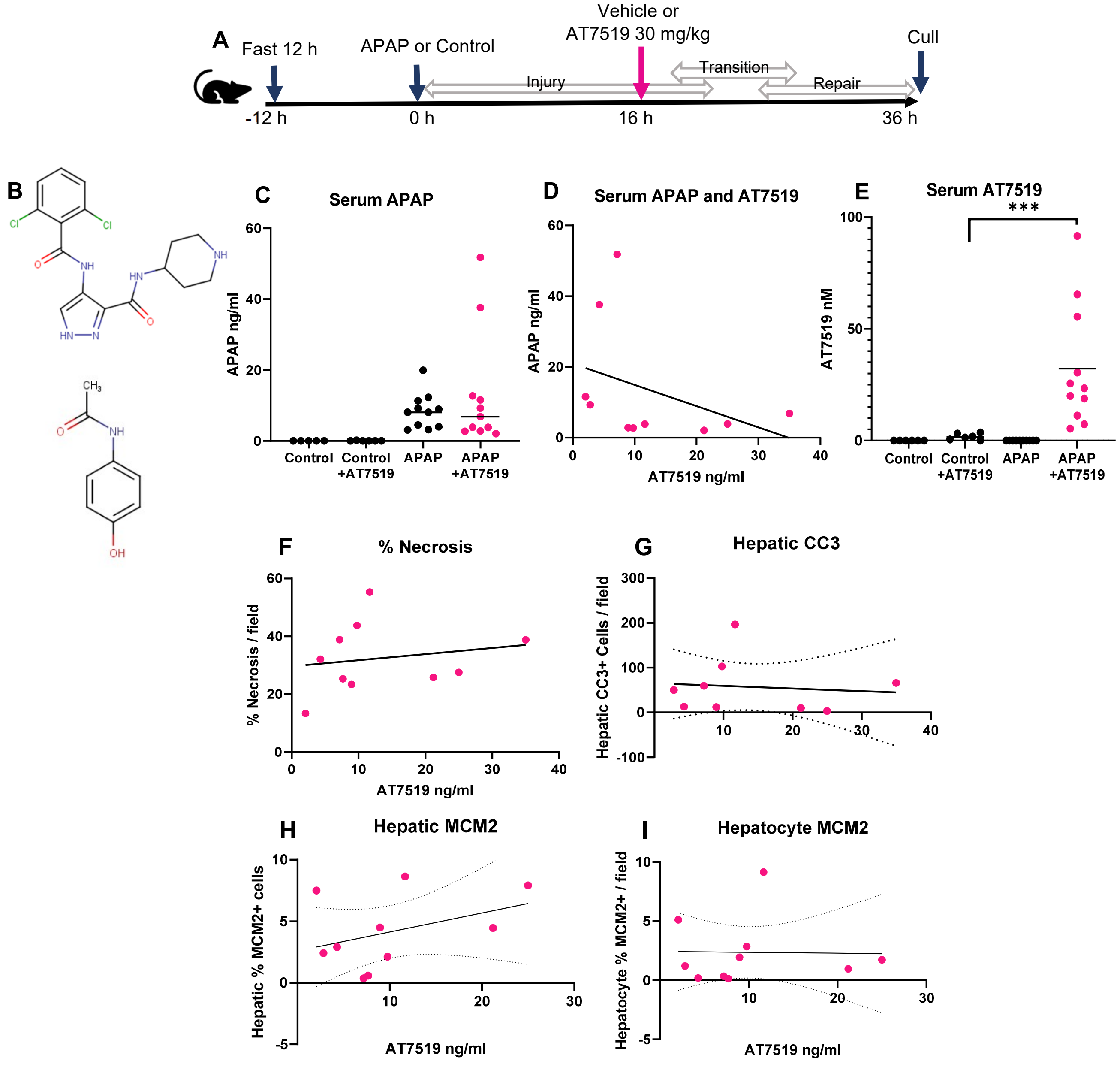

Fig. S4. Serum AT7519 concentrations do not correlate with injury or repair

(A), Model schematic. **(B),** Structure of AT7519 and APAP. **(C),** Serum APAP concentrations not increased with AT7519. **(D),** No correlation of serum AT7519 and APAP concentrations, R^2=0.007. **(E),** Serum AT7519 concentration increased following APAP-ALI, KS, p=0.0002. **(F-H),** Linear regression analysis of mouse serum AT7519 concentrations showed no correlation with; **F,** Average % necrosis R^2=0.2721, **G,** Average number of hepatic CC3+ cells R^2=0.1349, and **H,** Hepatic proliferation % MCM2 in all cells R^2=0.1508, and

I, % hepatocyte MCM2 expression R^2=0.00004. (n≥9/APAP groups). MCM2 minichromosome maintenance complex component 2, CC3 cleaved caspase 3. KS, Kolmogorov-Smirnov.

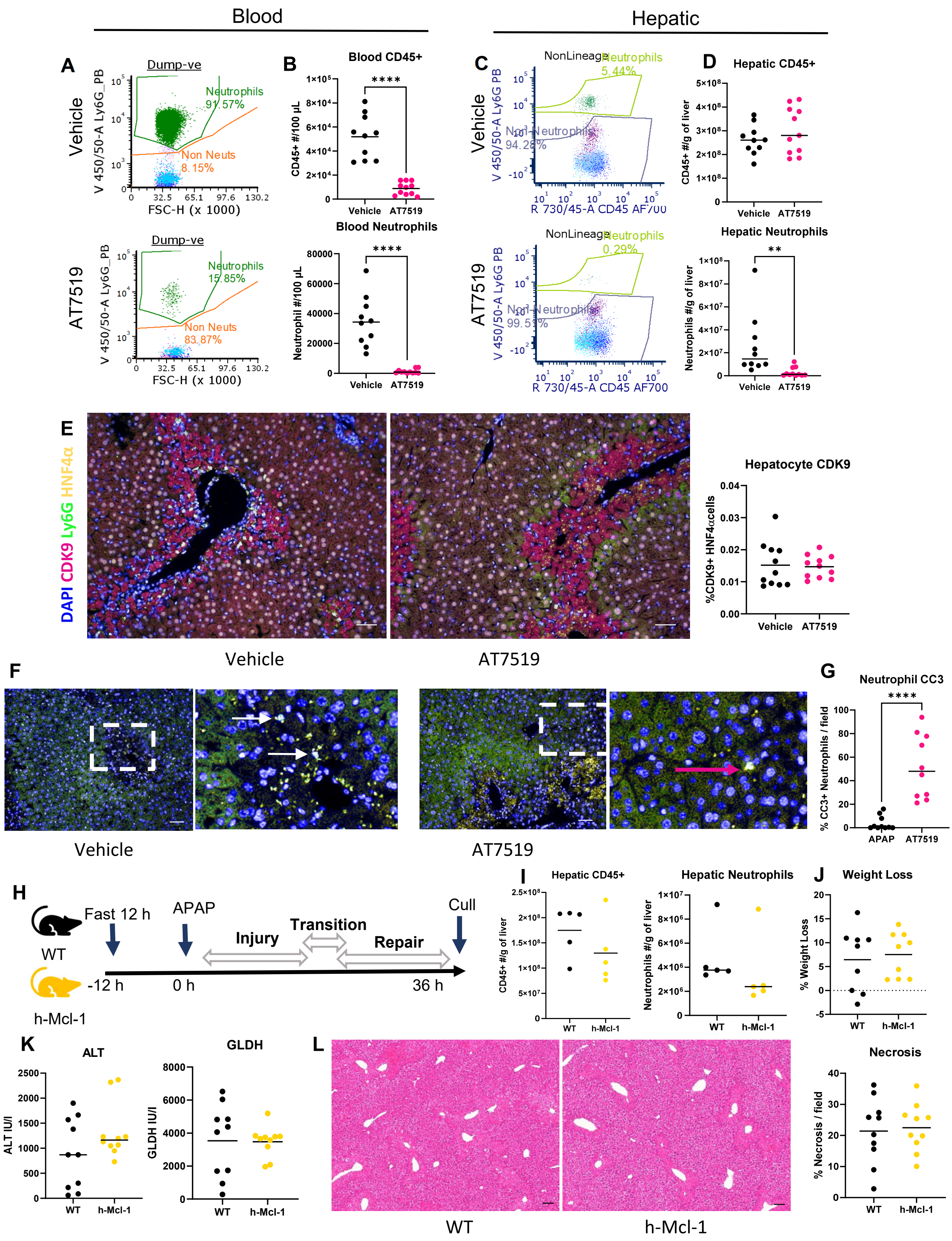

Fig. S5. AT7519 depletes hepatic neutrophils through CC3 and neutrophil survival is saturated

(A-G), 30 mg/kg AT7519 Neutrophil depletion ($n \geq 10$), assessed 16 h. **A,** Representative blood flow plots, **B,** AT7519 reduced circulating CD45+ cells, t-test, WC, $P<0.0001$, and neutrophils (CD45+Lin-Ly6G+) KS, $P<0.0001$. **C,** Representative hepatic flow plots **D,** CD45+ cells unaltered but reduced neutrophils KS, $p=0.0021$. **E,** Representative HNF4α, Ly6G and CDK9 labelled hepatic sections, and CDK9 quantification. **F,** Representative Ly6G and CC3 labelled hepatic sections. Magnified panels show increased CC3+ neutrophils (pink arrow) after AT7519. **G,** Percentage neutrophils CC3+, KS, $p<0.0001$. (**H),** Schematic, 350 mg/kg APAP treated WT and h-Mcl-1 mice, assessed 36 h. **(I),** Hepatic NPC flow cytometry; CD45+ cells and neutrophils not increased. **(J),** % weight loss, and **(K),** unaltered ALT and GLDH **(L),** Representative hepatic sections showing unaltered hepatic necrosis. KS, Kolmogorov-Smirnov. IHC scale bars 100 µm. IF scale bars 50 µm.

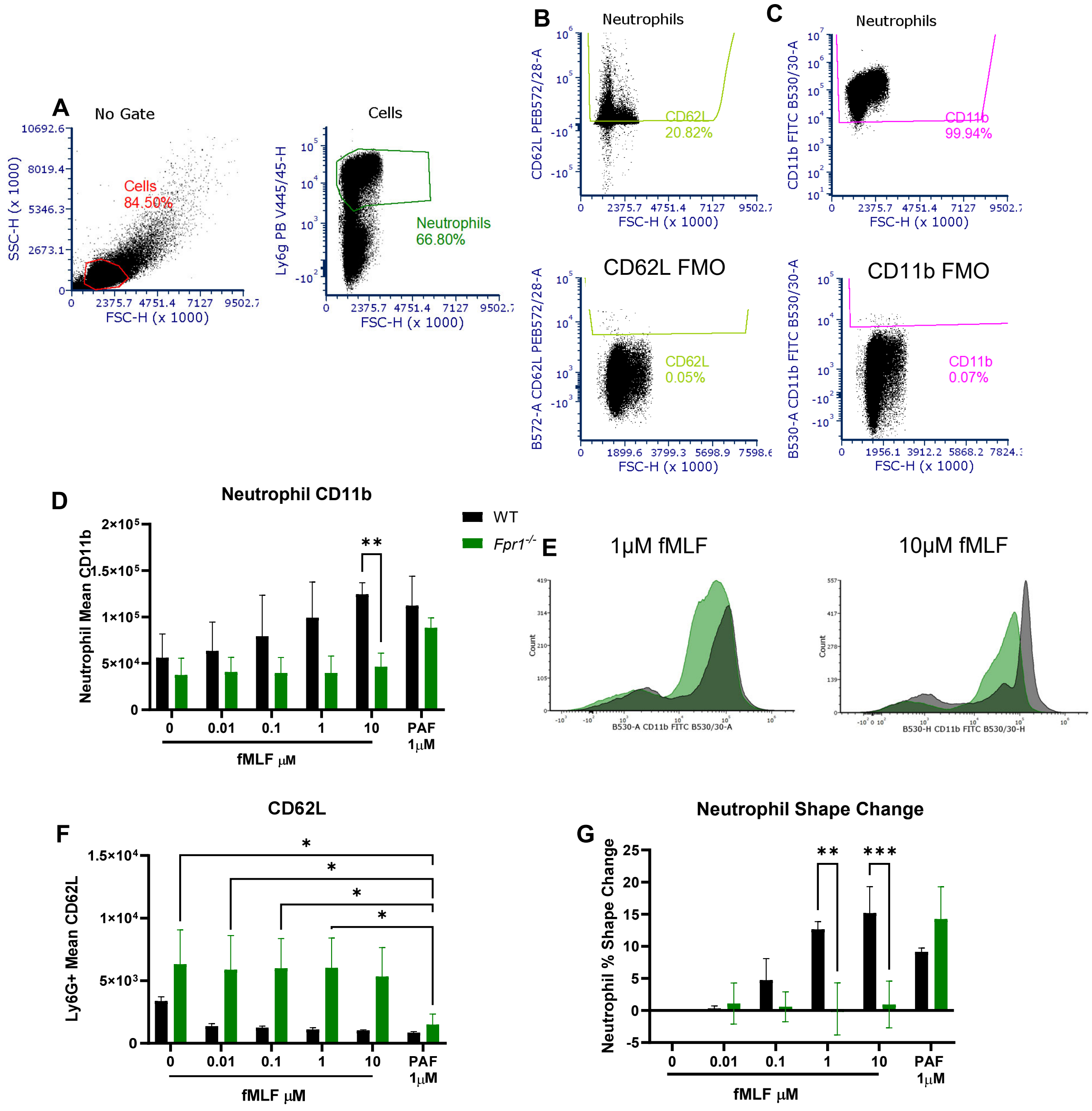

Fig. S6. *Fpr1*$^{-/-}$ neutrophils are not activated by formylated peptides

(A), Bone marrow derived mouse neutrophil flow cytometry gating. **(B),** CD62L+ and FMO. (**C**)**,** CD11b+ and FMO. **(D),** FMLF did not increase CD11b expression compared to WT, ANOVA, p=0.0335, Ší'ák's, p=0.0022 (10 μm FMLF). **(E),** Representative CD11b expression histogram (grey WT, green *Fpr1*$^{-/-}$). **(F),** *Fpr1*$^{-/-}$ neutrophils did not shed CD62L compared to positive control PAF, unlike WT neutrophils (0.01 Mm FMLF vs. PAF 1 μM, p= 0.0348, 0.1 Mm FMLF vs. PAF 1 μM, p= 0.0288, 1 Mm FMLF vs. PAF 1 μM, p= 0.0268, ANOVA with Tukeys). **(G)** *Fpr1*$^{-/-}$ neutrophils lower % shape change (FSC-H) than WT ANOVA, WT

vs KO p=0.0011, Ší'ák's, p=0.0014 (1 μm FMLF), p=0.0004 (10 μm FMLF). (n =3). PAF platelet activating factoR, FMLF N-Formylmethionyl-leucyl-phenylalanine.

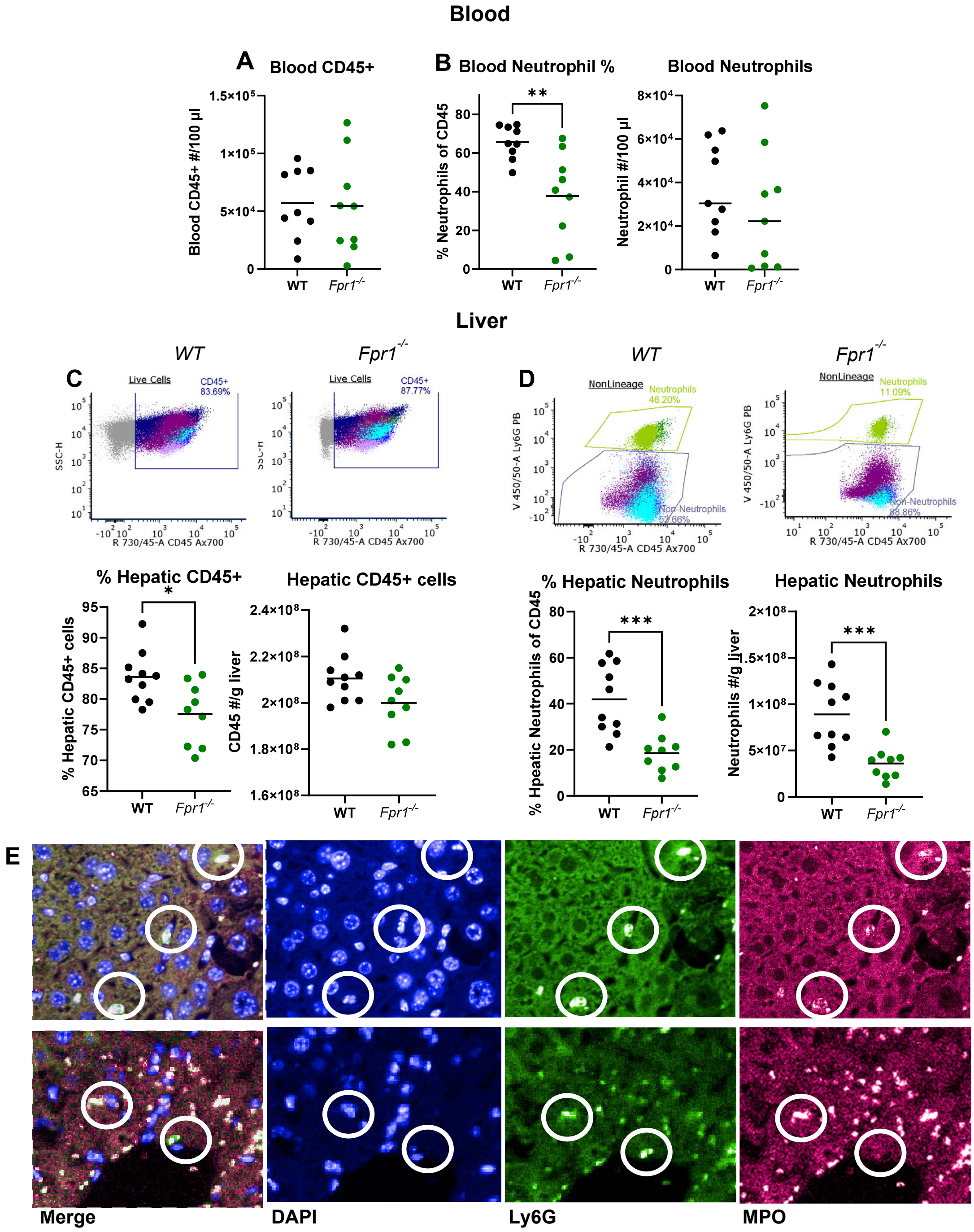

Fig. S7. Hepatic *Fpr1*$^{-/-}$ neutrophils are fewer and less activated following APAP-ALI

(A-D), Blood and hepatic NPC flow cytometry WT and *Fpr1*$^{-/-}$ mice 16 h after APAP. **A,** Circulating CD45+ unaltered. **B,** Circulating % neutrophils of CD45+ reduced (t-test, WC, p=0.006). **C,** Representative flow plots, hepatic CD45+% reduced significantly (t-test, p=0.01). **D,** Representative flow plots of hepatic neutrophils and quantification. Hepatic neutrophil % of CD45+ and absolute number reduced in *Fpr1*$^{-/-}$ mice (t-test,

p=0.0006). (**E**), Representative magnified hepatic sections individual channels from Figure 3, WT and *Fpr1*$^{-/-}$ mice 24 h after 350 mg/kg APAP i.p. Each data point represents an individual mouse (n≥9). WC Welch's correction. IF scale bars 50 µm.

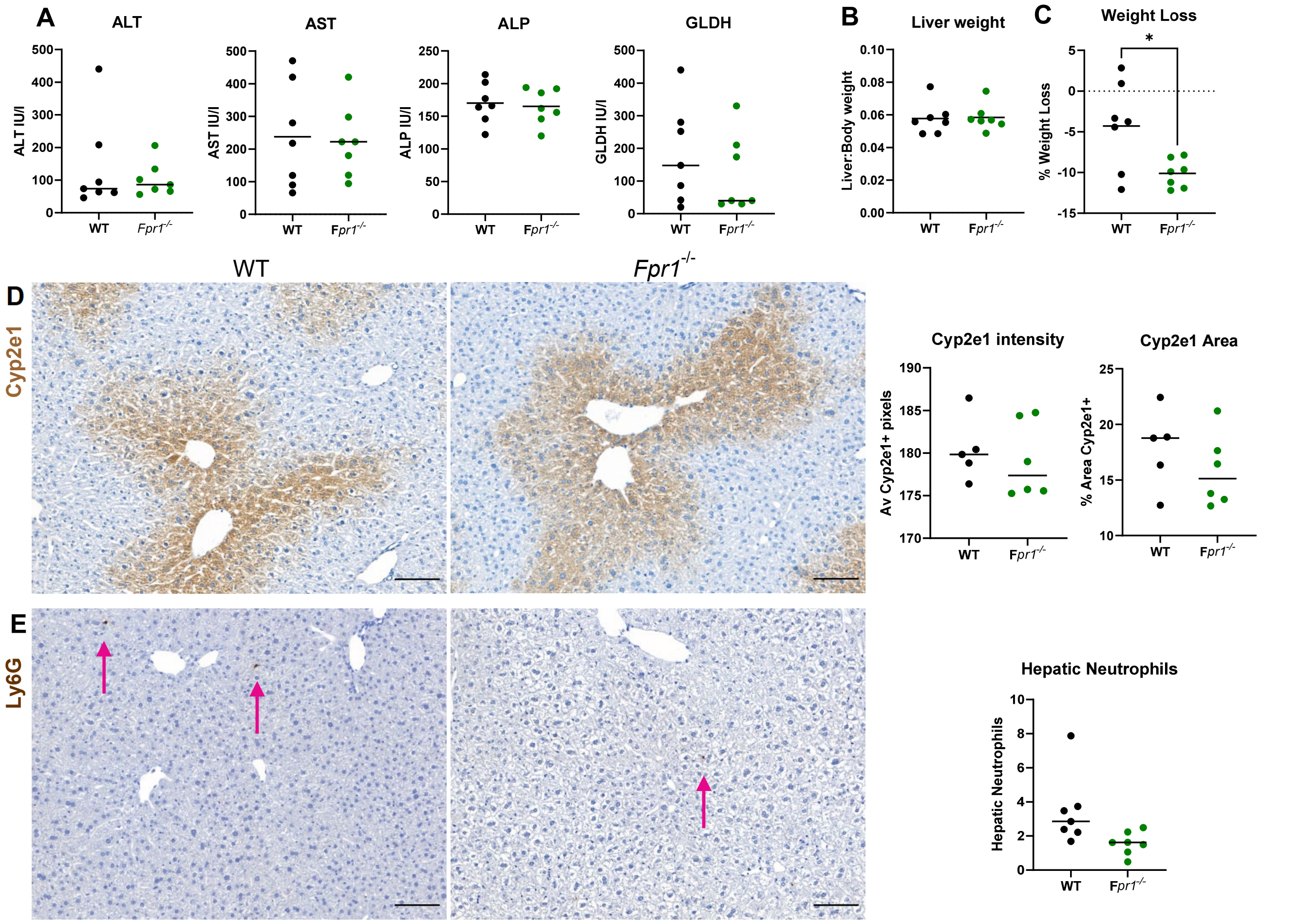

Fig. S8. Healthy *Fpr1*$^{-/-}$ mice hepatic parameters do not differ from WT

Results from WT and *Fpr1*$^{-/-}$ mice treated with APAP control. **(A),** Serum hepatic damage markers, ALT, AST, ALP and GLDH not different in *Fpr1*$^{-/-}$ mice. **(B),** Liver to body weight ratio not different to WT. **(C),** % weight loss was reduced in *Fpr1*$^{-/-}$ mice (t-test, WC, p=0.0291). **(D),** Representative Cyp2e1 labelled hepatic sections and quantification not in *Fpr1*$^{-/-}$ micl**(E),** Representative Ly6G labelled hepatic sections and neutrophil quantification showed no significant difference in *Fpr1*$^{-/-}$ mice. Each data point represents an individual mouse (n≥5). WC Welch's correction. IHC scale bars 100 µm.

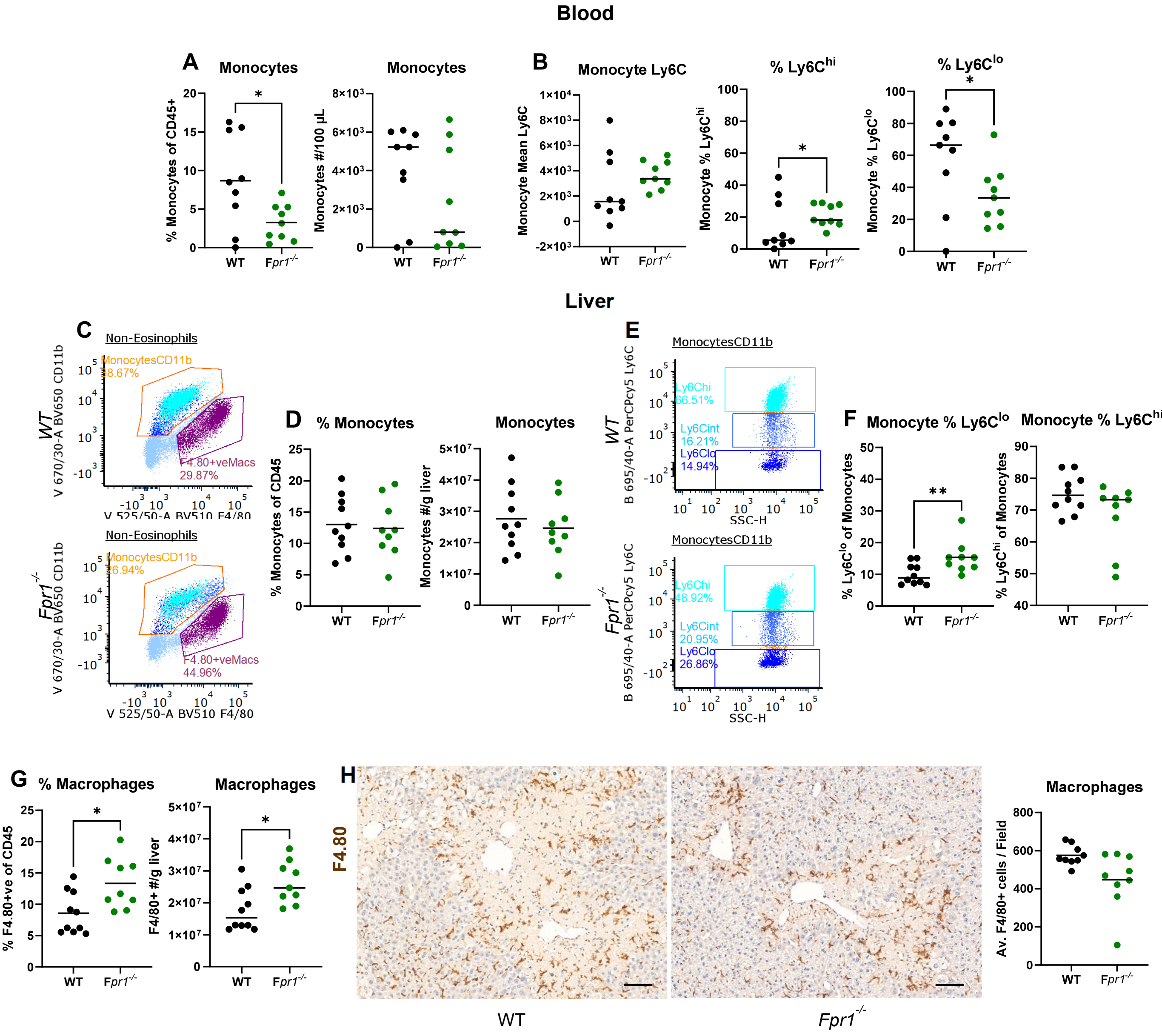

Fig. S9. Preventing FPR1-mediated neutrophil activation reduces monocyte activation

(A), Circulating monocyte % (t-test, WC, p=0.0312), reduced 16 h post APAP in *Fpr1*$^{-/-}$ mice. **(B),** Circulating monocyte higher % Ly6C^{hi} (KS, p=0.0336) and lower % Ly6C^{lo} (KS, p=0.0336) without FPR1 neutrophil activation. **(C),** Representative NPC flow plots of monocyte and macrophage populations. **(D),** Hepatic monocytes unaltered in *Fpr1*$^{-/-}$ Ie. **(E),** Representative plots of hepatic monocyte Ly6C. **(F),** Hepatic monocytes were less inflammatory with increased % Ly6C^{lo} (MW, p=0.0057). **(G),** *Fpr1*$^{-/-}$ mouse hepatic

F4/80+ macrophages % (t-test, $p=0.012$) and number (t-test, $p=0.0132$). (**H),** Representative F4/80-stained hepatic sections at 24 h post APAP show macrophage numbers stabilization. ($n \geq 9$). Representative flow plots are labelled with the parent gate and show cells/gates of interest. IHC scale bars 100 μm.

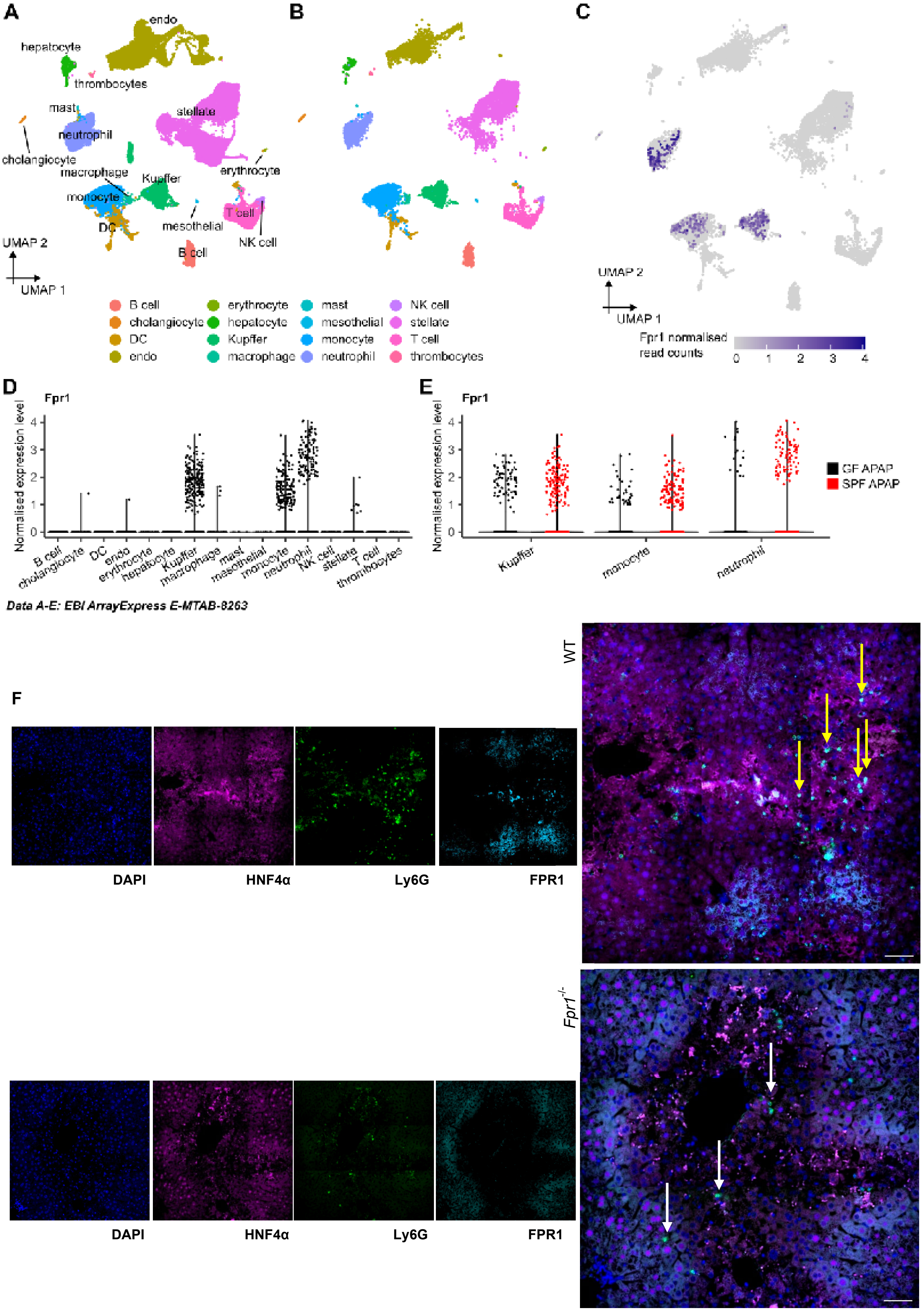
A
B
C
endo
hepatocyte
thrombocytes
mast
neutrophil
stellate
cholangiocyte
macrophage
Kupffer
erythrocyte
monocyte
T cell
DC
mesothelial
NK cell
B cell
UMAP 2
UMAP 1
B cell
cholangiocyte
DC
endo
erythrocyte
hepatocyte
Kupffer
macrophage
mast
mesothelial
monocyte
neutrophil
NK cell
stellate
T cell
thrombocytes
Fpr1 normalised read counts
0 1 2 3 4
D
Fpr1
Normalised expression level
E
Kupffer
monocyte
neutrophil
GF APAP
SPF APAP
Data A-E: EBI ArrayExpress E-MTAB-8263
F
WT
DAPI
HNF4α
Ly6G
FPR1
Fpr1-/-

Fig. S10. FPR1 is highly expressed on hepatic neutrophils following AALI

(A) UMAP of cell types in mouse liver scRNA-seq data (EBI ArrayExpress E-MTAB-8263). **(B)** UMAP of cells in APAP-treated specific pathogen-free (SPF) and germ free (GF) mice. **(C)** Fpr1 expression in SPF-APAP and GF-APAP conditions. **(D)** Violin plot of Fpr1 expression across cellypes. **(E)** Violin plot of Fpr1 expression in innate immune cells, split by experimental condition. **(F)** Representative HNF4α, Ly6G and FPR1 labelled hepatic sections, showing WT Ly6G+FPR1+ cells (yellow arrows) and Ly6G+FPR1- cells in *Fpr1*$^{-/-}$ mice (white arrows). Scale bars 50 μm

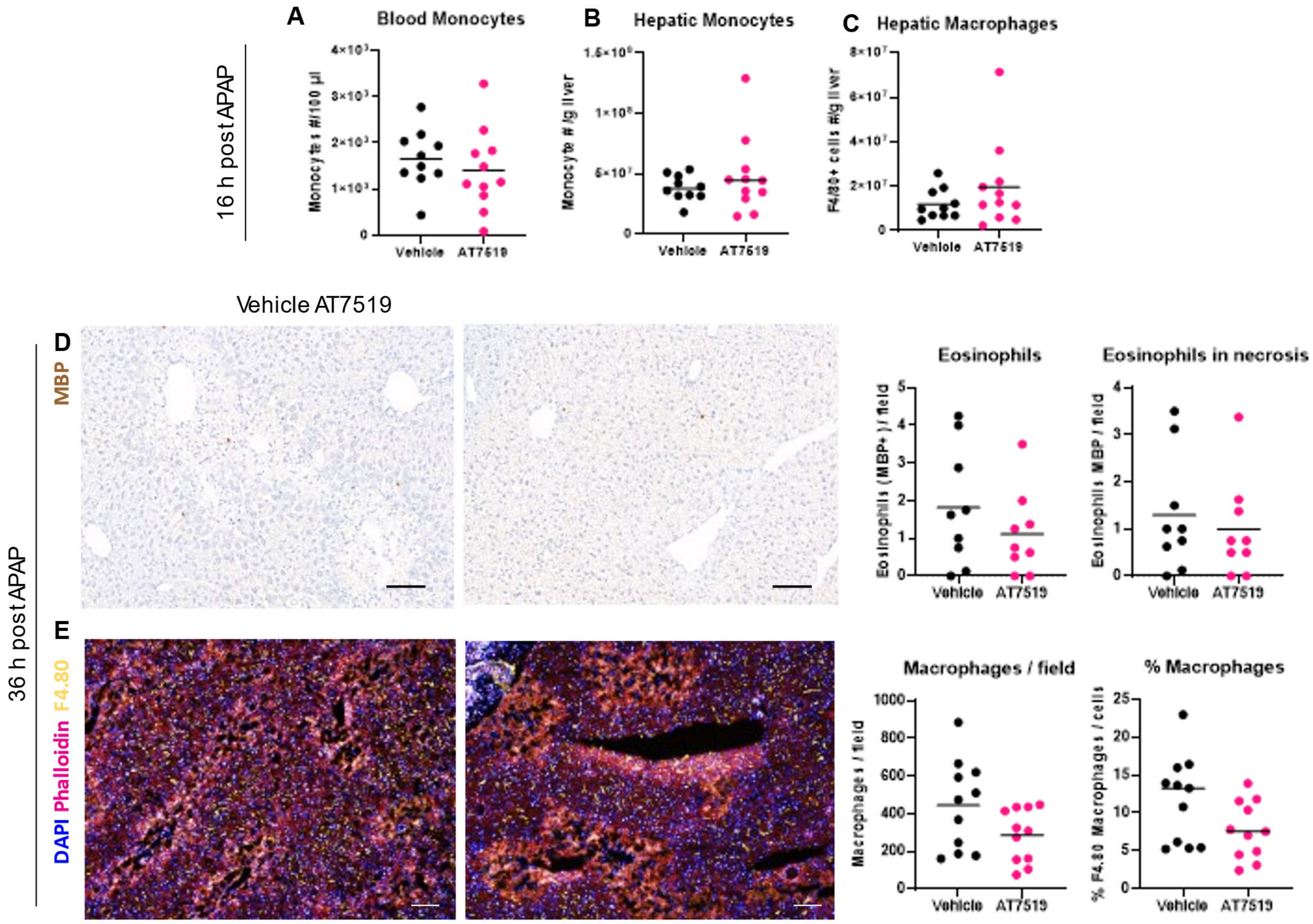

Fig. S11. AT7519-mediated neutrophil depletion is neutrophil specific

(A-C), Blood and Hepatic NPC flow cytometry 16 h after APAP, showed early 30 mg/kg AT7519 did not reduce **A,** blood monocytes, **B,** hepatic monocytes and **C,** hepatic macrophages. **(D-E),** Hepatic eosinophils and macrophages 36 h after 350 mg/kg APAP i.p. after 30 mg/kg AT7519 or vehicle at 16 h. **D,** Representative hepatic sections labelled with eosinophil major basic protein (MBP) (DAB) and quantification showed no reduction of all hepatic eosinophils or those within necrosis. **E,** Representative phalloidin and F4/80 labelled hepatic sections, demonstrated no reduction of F4/80+ macrophages / field or % positive cells. (n≥9). IHC scale bars 100 μm. IF scale bars 50 μm.

Supplementary methods

Single-cell RNA-seq analysis

We downloaded publicly-available pre-processed scRNA-seq data of mouse liver cells (n=56,527 cells) from EBI ArrayExpress (E-MTAB-8263) (Kolodziejczyk et al., 2020). Data were imported into R and subject to standard normalization and data scaling using the Seurat Bioconductor package (v4.4.0)(Hao et al., 2021). Dimensionality reduction was computed with PCA and visualised using UMAP (with the first 50 principal components, as in the original study). Data were subset to cells from paracetamol (APAP)-treated, specific pathogen-free (SPF) and germ free (GF) mice (total n=15,061 cells) for downstream analysis. Gene expression was visualised using Seurat functions.

Supplementary tables

Table S1. Flow cytometry panel for the hepatic innate immune cells

<table>
<tr><th>Marker</th><th>Clone</th><th>Fluorophore</th><th>Laser/Channel</th><th>Manufact</th><th>Man. Conc. mg/ml</th><th>Dilution</th></tr>
<tr><td>CD45</td><td>30-F11</td><td>AF700</td><td>R730/45</td><td>Biolegend</td><td>0.5</td><td>1:100</td></tr>
<tr><td>CD11b</td><td>M1/70</td><td>BV650</td><td>V675/30</td><td>eBio</td><td>0.2</td><td>1:100</td></tr>
<tr><td>Ly6G</td><td>1A8</td><td>PB</td><td>V445/45</td><td>BD Pharm</td><td>0.5</td><td>1:100</td></tr>
<tr><td>CD62L</td><td>MEL-14</td><td>FITC</td><td>B530/30</td><td>Biolegend</td><td>0.5</td><td>1:200</td></tr>
<tr><td>Siglec F</td><td>E50-2440</td><td>PE</td><td>YG582/15</td><td>Biolegend</td><td>0.2</td><td>1:400</td></tr>
<tr><td>F4/80</td><td>BM8</td><td>APC</td><td>R675/30</td><td>Biolegend</td><td>0.2</td><td>1:100</td></tr>
<tr><td>Ly6C</td><td>HK1.4</td><td>PerCP/Cy5.5</td><td>B675/30</td><td>Biolegend</td><td>0.2</td><td>1:100</td></tr>
<tr><td>CD3</td><td>145-2C11</td><td rowspan="3">PE/Cy7</td><td rowspan="3">YG780/60</td><td>Biolegend</td><td rowspan="3">0.2</td><td rowspan="3">1:200</td></tr>
<tr><td>CD19</td><td>6D5</td><td>Biolegend</td></tr>
<tr><td>NK1.1</td><td>PK136</td><td>Biolegend</td></tr>
<tr><td>L/D</td><td></td><td>E780</td><td>R780/60</td><td>eBio</td><td>-</td><td>1:1000</td></tr>
</table>

Table S2. Flow cytometry panel for hepatic innate immunity and neutrophil activation

Marker	Clone	Fluorophore	Laser/Channel	Manufact	Man. Conc. mg/ml	Dilution
CD45	30-F11	AF700	R730/45	Biolegend	0.5	1:100
CD11b	M1/70	BV650	V675/30	eBio	0.2	1:100
Ly6G	1A8	PB	V445/45	BD Pharm	0.5	1:100
MPO	2D4	FITC	B530/30	Abcam ab90812	0.1	1:100
CD62L	MEL-14	APC	R675/30	Biolegend 104412	0.2	1:100
Siglec F	E50-2440	PE	YG582/15	BD Pharm	0.2	1:400
F4/80	BM8	BV510	V525/50	Biolegend	0.2	1:100
Ly6C	HK1.4	PerCP/Cy5.5	B675/30	Biolegend	0.2	1:100
CD3	145-2C11	PE/Cy7	Lineage Dump channel YG780/60	Biolegend	0.2	1:200
CD19	6D5			Biolegend		1:200
NK1.1	PK136			Biolegend		1:200
L/D		E780	R780/60	eBio		1:1000

Table S3. Flow cytometry panel for blood innate immunity

Marker	Clone	Fluorophore	Laser/Channel	Manufact	Man. Conc. mg/ml	Dilution
CD45	30-F11	AF700	R730/45	Biolegend	0.5	1:100
CD11b	M1/70	BV650	V675/30	eBio	0.2	1:100
Ly6G	1A8	PB	V445/45	BD Pharm	0.5	1:100
CD62L	MEL-14	FITC	B530/30	Biolegend	0.5	1:200
Siglec F	E50-2440	PE	YG582/15	Biolegend	0.2	1:400
F4/80	BM8	APC	R675/30	Biolegend	0.2	1:100
Ly6C	HK1.4	PerCP/Cy5.5	B675/30	Biolegend	0.2	1:100
CD3	145-2C11	PE/Cy7	YG780/60	Biolegend	0.2	1:200
CD19	6D5			Biolegend		
NK1.1	PK136			Biolegend		
L/D		E780	R780/60	eBio		1:1000

Table S4. Flow cytometry panel for blood innate immunity and neutrophil activation

Marker	Clone	Fluorophore	Laser/Channel	Manufact	Man. Conc. mg/ml	Dilution
CD45	30-F11	AF700	R730/45	Biolegend	0.5	1:100
CD11b	M1/70	BV650	V675/30	eBio	0.2	1:100
Ly6G	1A8	PB	V445/45	BD Pharm	0.5	1:100
MPO	2D4	FITC	B530/30	Abcam ab90812	0.1	1:100
CD62L	MEL-14	APC	R675/30	Biolegend 104412	0.2	1:100
Siglec F	E50-2440	PE	YG582/15	BD Pharm	0.2	1:400
CD115	AF598	BV510	R675/30	BD Pharm	0.2	1:100
Ly6C	HK1.4	PerCP/Cy5.5	B675/30	Biolegend	0.2	1:100
CD3	145-2C11			Biolegend		1:200
CD19	6D5	PE/Cy7	Lineage Dump channel YG780/60	Biolegend	0.2	1:200
NK1.1	PK136			Biolegend		1:200
L/D		E780	R780/60	eBio		1:1000

Table S5. Primary antibodies used for immunohistochemistry and immunofluorescence

Antibody	Clone	Dilution from Stock	Antigen retrieval	Manufacturer	Cat. Number	Host
Active Caspase 3	C92-605	1:200	TE	BD Pharmingen	BD559565	Rb
CDK9	EPR31197	1:200	TE	Abcam	ab76320	Rb
Cyp2e1	na	1:500	NaCi	Atlas	HPA009128	Rb
F4/80	A3-1	1:100	PK	Abcam	ab6640	Rt
FPR1	Polyclonal	1:150	NaCi	ThermoFisher Scientific	PA5140980	Rb
HNF4α	H1415	1:200	TE	Perseus Proteomics	PP-H1415-00	Ms
Ly6G	1A8	1:1000 IHC 1:500 IF	TE or NaCi	Biolegend	127602	Rt
MBP	MT2-14.7.3	1:1000	Digest-All™ 3 pepsin	Mayo Clinic, Arizona	Mayo Clinic, Arizona	Rt
MCM-2	Polyclonal	1:200	TE	Cell Signalling	4007S	Rb
MPO	Polyclonal	1:50	TE	Abcam	Ab9535	Rb
Phalloidin Reagent	na iFluor 647	1:1000	TE of NaCi	Abcam	Ab176759	na

Abbreviation List: Gt; Goat, Hm; Human, Ms; Mouse Rb; Rabbit, Rt; Rat, TE; TrisEDTA (ph8), PK; Proteinase K, HNF4α

Table S6. Secondary antibodies used for immunohistochemistry and immunofluorescence

Conjugate	Target Species	Fluorophore	Dilution from Stock	Manufacturer	Cat. Number	Host
Fluorescent	Rt	488	1:200	Invitrogen	A21208	Dk
Fluorescent	Ms	488	1:200	Invitrogen	A21202	Dk
Fluorescent	Rt	555	1:200	Invitrogen	A48270	Dk
Fluorescent	Rb	555	1:200	Invitrogen	A31572	Dk
Fluorescent	Ms	555	1:200	Invitrogen	A32773	Dk
Flourescent	Gt	555	1:200	Invitrogen	A32816	Dk
Fluorescent	Rb	647	1:200	Invitrogen	A32795	Dk
Fluorescent	Rt	647	1:200	Invitrogen	A78947	Dk
Biotinylated	Rb	-	1:200	Vector	BA-1000	Gt
Biotinylated	Rt	-	1:200	Vector	BA-9400	Gt
Biotinylated	Ms	-	1:200	Vector		Gt

Abbreviation List: Dk; Donkey,Gt; Goat, Ms; Mouse Rb; Rabbit, Rt; Rat

Supplementary references

Hao, Y., Hao, S., Andersen-Nissen, E., et al. (2021). Integrated analysis of multimodal single-cell data. *Cell*, *184*(13), 3573-3587.e29. https://doi.org/10.1016/j.cell.2021.04.048

Kolodziejczyk, A. A., Federici, S., Zmora, N., et al (2020). Acute liver failure is regulated by MYC- and microbiome-dependent programs. *Nature Medicine*, *26*(12), 1899–1911. https://doi.org/10.1038/s41591-020-1102-2
